# Supplementary material for: Estimating BMI distributions by age and sex for local authorities in England: a small area estimation study
Source: BMJ Open. 2022 Jun 21;12(6):e060892. doi: 10.1136/bmjopen-2022-060892 (PMC9226908; doi:10.1136/bmjopen-2022-060892)
Supplement: Supplementary data [file bmjopen-2022-060892supp001.pdf]

Appendix tables

Appendix table 1: Variable mapping showing matched variables in HSE 2018 and the Census microdata and any adaptations to variables necessary to make them equivalent.

Characteristic	Census 2011 microdata Variable name	Census value	Census label	New value	New Label
**Age**	**AGEC**	1	0-4 year olds	(Missing)	
		2	5-9 year olds	(Missing)	
		3	10-15 year olds	(Missing)	
		4	16-18 year olds	17.5	16-18 year olds
		5	19-24 year olds	22	19-24 year olds
		6	25-29 year olds	27.5	25-29 year olds
		7	30-34 year olds	32.5	30-34 year olds
		8	35-39 year olds	37.5	35-39 year olds
		9	40-44 year olds	42.5	40-44 year olds
		10	45- 49 year olds	47.5	45- 49 year olds
		11	50- 54 year olds	52.5	50- 54 year olds
		12	55-59 year olds	57.5	55-59 year olds
		13	60-64 year olds	62.5	60-64 year olds
		14	65-69 year olds	67.5	65-69 year olds
		15	70-74 year olds	72.5	70-74 year olds
		16	75-79 year olds	77.5	75-79 year olds
		17	80-84 year olds	82.5	80-84 year olds
		18	85-89 year olds	87.5	85-89 year olds
		19	90+	F=93.0 M=92.4(1)	90+ year olds
**Sex**	**SEX**	1	male	0	male
		2	female	1	female
**Ethnicity**	**AGGETHPUK113**	1	White	1	White
		2	Mixed/multiple ethnic groups	4	Mixed/multiple
		3	Asian/Asian British: Indian	3	Asian
		4	Asian/Asian British: Pakistani	3	Asian
		5	Asian/Asian British: Bangladeshi	3	Asian
		6	Asian/Asian British: Chinese	3	Asian
		7	Asian/Asian British: Other Asian	3	Asian
		8	Black/African/Caribbean/Black British: African	2	Black

		9	Black/African/Caribbean/Black British: Black Caribbean	2	Black
		10	Black/African/Caribbean/Black British: Other Black	2	Black
		11	Other ethnic group	5	Any other group
**Unemployed**	**ECOPUK1**	1	Econ. Active (excl. Students), Employee, Part-time	0	Not unemployed
		2	Econ. Active (excl. Students), Employee, Full-time	0	Not unemployed
		3	Econ. Active (excl. Students), Self-employed with employees, Part-time	0	Not unemployed
		4	Econ. Active (excl. Students), Self-employed with employees, Full-time	0	Not unemployed
		5	Econ. Active (excl. Students), Self-employed no employees, Part-time	0	Not unemployed
		6	Econ. Active (excl. Students), Self-employed no employees, Full-time	0	Not unemployed
		7	Econ. Active (excl. Students), Unemployed(2)	1	Unemployed
		8	Economically Active Full-time Students, In Employment	0	Not unemployed
		9	Economically Active Full-time Students, Unemployed(2)	1	Unemployed
		10	Economically Inactive, Retired	0	Not unemployed
		11	Economically Inactive, Student	0	Not unemployed
		12	Economically Inactive, Looking after home/family	0	Not unemployed
		13	Economically Inactive, Permanently sick/disabled	0	Not unemployed
		14	Economically Inactive, Other	0	Not unemployed
**Student**	**ECOPUK1**	1	Econ. Active (excl. Students), Employee, Part-time	0	Not a student
		2	Econ. Active (excl. Students), Employee, Full-time	0	Not a student
		3	Econ. Active (excl. Students), Self-employed with employees, Part-time	0	Not a student
		4	Econ. Active (excl. Students), Self-employed with employees, Full-time	0	Not a student

		5	Econ. Active (excl. Students), Self-employed no employees, Part-time	0	Not a student
		6	Econ. Active (excl. Students), Self-employed no employees, Full-time	0	Not a student
		7	Econ. Active (excl. Students), Unemployed(2)	0	Not a student
		8	Economically Active Full-time Students, In Employment	1	Student
		9	Economically Active Full-time Students, Unemployed(2)	1	Student
		10	Economically Inactive, Retired	0	Not a student
		11	Economically Inactive, Student	1	Student
		12	Economically Inactive, Looking after home/family	0	Not a student
		13	Economically Inactive, Permanently sick/disabled	0	Not a student
		14	Economically Inactive, Other	0	Not a student
**Has a degree or equivalent**	**hlqupuk11**	10	No academic or professional qualifications	0	
		11	Level 1 (0-4 GCSE, O level, or equivalents)	0	
		12	Level 2 (5+ GCSE, O level, 1 A level, or equivalents)	0	
		13	Apprenticeship	0	
		14	Level 3 (2+ A levels, or equivalents)	0	
		15	Level 4+ (degree, postgrad, professional quals)	1	Has a degree or equivalent
		16	Other (vocational/foreign/outside UK quals)	0	
		-9	Not applicable	(Missing)	
**Self-reported general health**	**HEALTH**	1	Very good	1	Very good
		2	Good	2	Good
		3	Fair	3	Fair
		4	Bad	4	Bad
		5	Very bad	5	Very bad
**Deprivation**	**IMDQUINTE**	1	Least deprived	1	Least deprived
		2	Second least	2	Second least
		3	Intermediate	3	Intermediate
		4	Second most	4	Second most
		5	Most deprived	5	Most deprived
		-8	unknown	(Missing)	

	**HSE 2017**				
	**Variable name**	**HSE value**	**HSE label**	**New value**	**New Label**
**Age**	**Age16g10**	1	0-1 year olds	(Missing)	
		2	2-4 year olds	(Missing)	
		3	5-7 year olds	(Missing)	
		4	8-10 year olds	(Missing)	
		5	11-12 year olds	(Missing)	
		6	13-15 year olds	(Missing)	
		7	16-19 year olds	18	16-19 year olds
		8	20-24 year olds	22.5	20-24 year olds
		9	25-29 year olds	27.5	25-29 year olds
		10	30-34 year olds	32.5	30-34 year olds
		11	35-39 year olds	37.5	35-39 year olds
		12	40-44 year olds	42.5	40-44 year olds
		13	45- 49 year olds	47.5	45- 49 year olds
		14	50- 54 year olds	52.5	50- 54 year olds
		15	55-59 year olds	57.5	55-59 year olds
		16	60-64 year olds	62.5	60-64 year olds
		17	65-69 year olds	67.5	65-69 year olds
		18	70-74 year olds	72.5	70-74 year olds
		19	75-79 year olds	77.5	75-79 year olds
		20	80-84 year olds	82.5	80-84 year olds
		21	85-89 year olds	87.5	85-89 year olds
		22	90+	F=93.0, M=92.4(1)	90+ year olds
**Sex**	**Sex**	1	Male	0	male
		2	Female	1	female
		3	Refused	(Missing)	
		4	Don't know	(Missing)	
		5	Not applicable	(Missing)	
**Ethnicity**	**Origin2**	1	White	1	White
		2	Black	2	Black
		3	Asian	3	Asian
		4	Mixed/multiple ethnic background	4	Mixed/multiple
		5	Any other ethnic group	5	Any other group
		6	Refusal	(Missing)	

		7	Don't know	(Missing)	
**Unemployed**	**Activb2**	1	Going to school or college full-time	0	Not unemployed
		2	In paid employment or self-employed	0	Not unemployed
		3	Doing unpaid work for family business	0	Not unemployed
		4	Waiting to take up paid work already obtained	0	Not unemployed
		5	Looking for paid work/ Govt. training scheme	1	Unemployed
		6	Intending to look for work, but temporary sickness	0	Not unemployed
		7	Permanently unable to work, long-term sickness	0	Not unemployed
		8	Retired from paid work	0	Not unemployed
		9	Looking after home or family	0	Not unemployed
		95	Doing something else (specify)	0	Not unemployed
		-9	No answer/refused	(Missing)	
		-8	Don't know	(Missing)	
		-1	Not applicable	(Missing)	
**Student**	**Activb2**	1	Going to school or college full-time	1	Student
		2	In paid employment or self-employed	0	Not a student
		3	Doing unpaid work for family business	0	Not a student
		4	Waiting to take up paid work already obtained	0	Not a student
		5	Looking for paid work/ Govt. training scheme	0	Not a student
		6	Intending to look for work, but temporary sickness	0	Not a student
		7	Permanently unable to work, long-term sickness	0	Not a student
		8	Retired from paid work	0	Not a student
		9	Looking after home or family	0	Not a student
		95	Doing something else (specify)	0	Not a student
		-9	No answer/refused	(Missing)	
		-8	Don't know	(Missing)	
		-1	Not applicable	(Missing)	
**Has a degree or equivalent**	**topqual3**	1	NVQ4/NVQ5/Degree or equiv	1	Has a degree or equivalent

		2	Higher ed below degree	0	
		3	NVQ3/GCE A Level equiv	0	
		4	NVQ2/GCE O Level equiv	0	
		5	NVQ1/CSE other grade equiv	0	
		6	Foreign/other	0	
		7	No qualification	0	
		-9	Refused	(Missing)	
		-8	Don't know	(Missing)	
		-1	Not applicable	(Missing)	
**Self-reported general health**	**GenHelf**	1	Very good	1	Very good
		2	Good	2	Good
		3	Fair	3	Fair
		4	Bad	4	Bad
		5	Very bad	5	Very bad
		-9	Refused	(Missing)	
		-8	Don't know	(Missing)	
		-1	Not applicable	(Missing)	
**Deprivation**	**(Merged from external dataset**(15))	1	Least deprived - 0.48 ->8.37	1	Least deprived
		2	Second least - 8.37 -> 13.92	2	Second least
		3	Intermediate - 13.92- >21.43	3	Intermediate
		4	Second most - 21.43 -> 33.88	4	Second most
		5	Most deprived - 33.88 -> 92.60	5	Most deprived

(1) Average age for over-90s in most recent ONS mid-year estimates of the England population is 93.0 years for females and 92.4 years for males.

(2) International Labour Organisation definition: seeking work and ready to start in 2 weeks, and Waiting to start a job already obtained and available to start within 2 weeks.

Appendix table 2: Percent of missing data in source datasets by variable used (England only, respondents aged 17 and over). *IMD was merged into Census data at the lower tier local authority level from ONS data.(15)

Dataset:	Census microdata		HSE 2018	
	Variable	% missing	Variable	% missing
	age	0.00	age	0.00
	sex	0.00	sex	0.00
	ethnicity	1.28	ethnicity	0.39
	student	1.28	student	0.21

has degree	1.28	has degree	0.46
health	1.28	health	0.04
No of cars	2.21	No of cars	0.06
IMD*	-	IMD	0.00
		BMI	18.33

Appendix table 3: Percent of missing data for the BMI variable used in HSE 2018 (BMIval2) by demographic group

Breakdown variable	Total records	Of which percent missing
Age		
18	281	17.8
22.5	366	19.4
27.5	487	17.5
32.5	581	18.1
37.5	647	14.2
42.5	684	15.8
47.5	668	15.3
52.5	686	17.9
57.5	733	16.2
62.5	634	17.5
67.5	614	14.3
72.5	625	20.0
77.5	444	22.3
82.5	298	30.5
87.5	177	36.2
90+	72	45.9
Sex		
Female	4461	18.7
Male	3536	17.8
IMD quintile		
1 (lowest deprivation)	1653	15.5
2	1707	18.3
3	1554	19.0
4	1557	19.1
5	1526	20.1

Appendix table 4: Baseline population characteristics. Age given as mean (median, interquartile range). Remaining variables given as number (percent of total).

Number	BMI up to 25 N= 2,198	BMI over 25 N= 4,333	Total N= 6,531
Age (years)	42.5 (32.5,62.5)	52.5 (42.5,67.5)	52.5 (37.5,67.5)
Males	1,337 (60.8%)	2,289 (52.8%)	3,626 (55.5%)
Ethnicity			
White	1,916 (87.2%)	3,828 (88.5%)	5,744 (88.1%)
Black	47 (2.1%)	131 (3.0%)	178 (2.7%)
Asian	184 (8.4%)	288 (6.7%)	472 (7.2%)
Mixed	34 (1.5%)	47 (1.1%)	81 (1.2%)
Any other	15 (0.7%)	33 (0.8%)	48 (0.7%)
IMD quintile			
1 (Least deprived)	501 (22.8%)	850 (19.6%)	1,351 (20.7%)
2	512 (23.3%)	931 (21.5%)	1,443 (22.1%)
3	433 (19.7%)	825 (19.0%)	1,258 (19.3%)
4	393 (17.9%)	867 (20.0%)	1,260 (19.3%)
5 (Most deprived)	359 (16.3%)	860 (19.8%)	1,219 (18.7%)
Self-rated health			
Very good	971 (44.2%)	1,238 (28.6%)	2,209 (33.8%)
Good	856 (39.0%)	1,880 (43.4%)	2,736 (41.9%)
Fair	259 (11.8%)	876 (20.2%)	1,135 (17.4%)
Bad	82 (3.7%)	253 (5.8%)	335 (5.1%)
Very bad	29 (1.3%)	86 (2.0%)	115 (1.8%)

Appendix figures

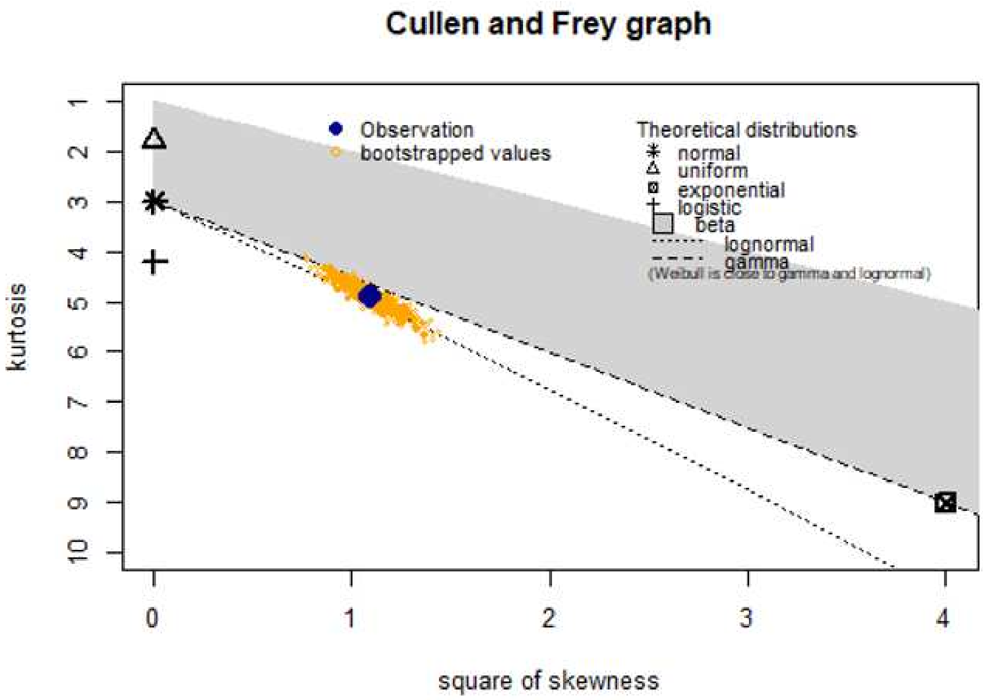

Appendix figure 1: Cullen-Frey plot exploring the mean-variance relationship of the model dependent BMI variable from HSE 2018, showing a marginally closer conformity with a lognormal than gamma distribution.

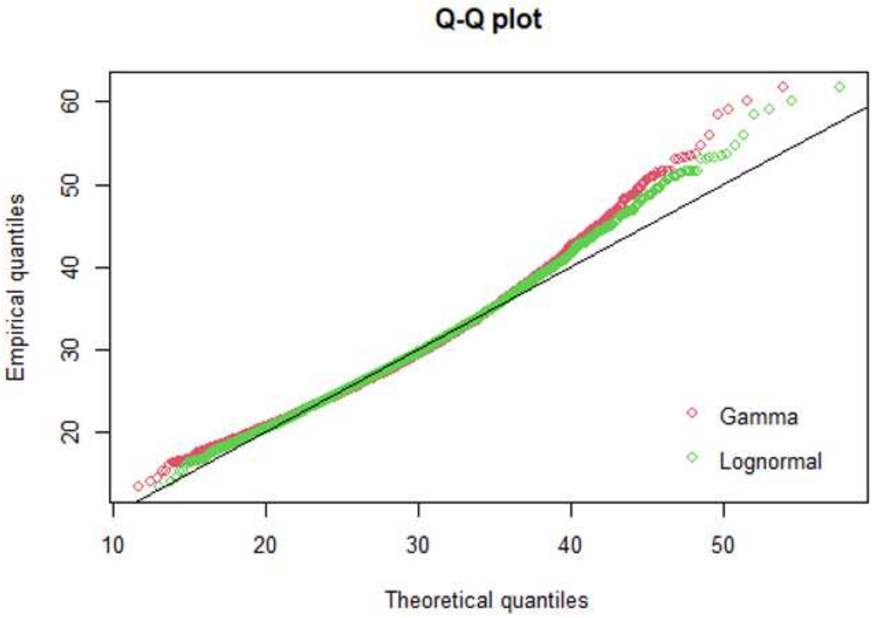

Appendix figure 2: Q-Q plot exploring the mean-variance relationship of the model dependent BMI variable from HSE 2018, detailing the deviation from gamma and lognormal distributions, showing a marginally closer conformity with a lognormal than gamma distribution.

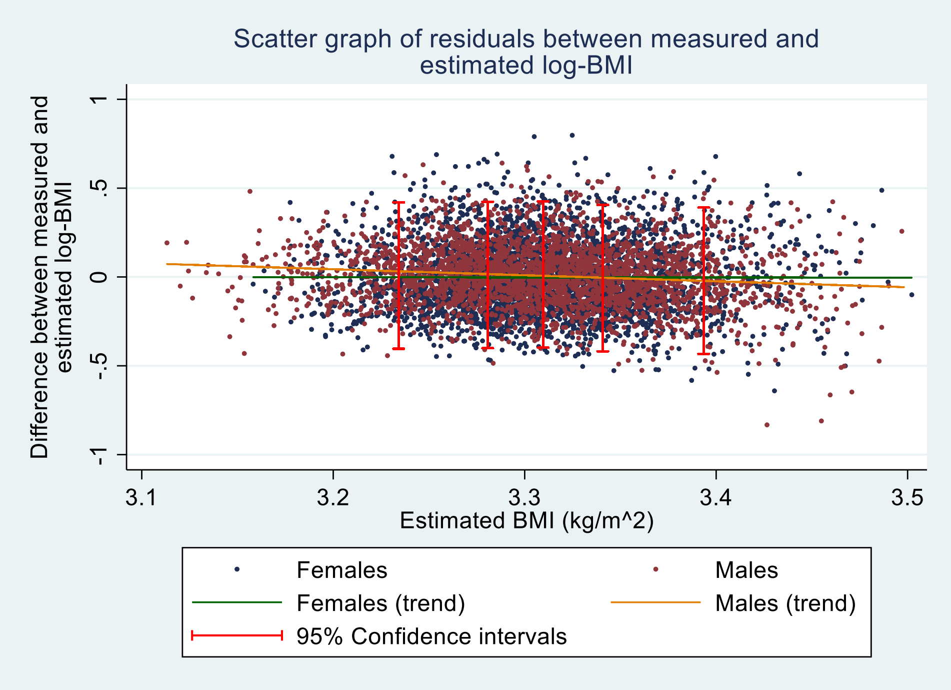

Appendix figure 3: Plot of residuals between measured log-BMI in the HSE 2018 and model-based estimates.

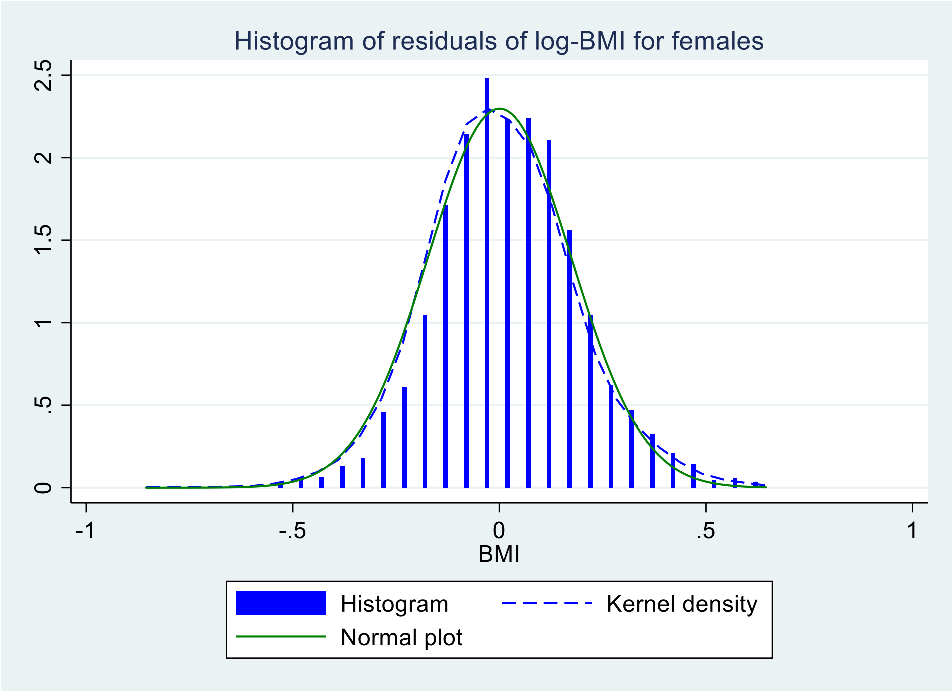

Appendix figure 4a: Histograms of residuals of between measured and estimated log-BMI for females to with kernel density and normal plot to examine for deviation from normality.

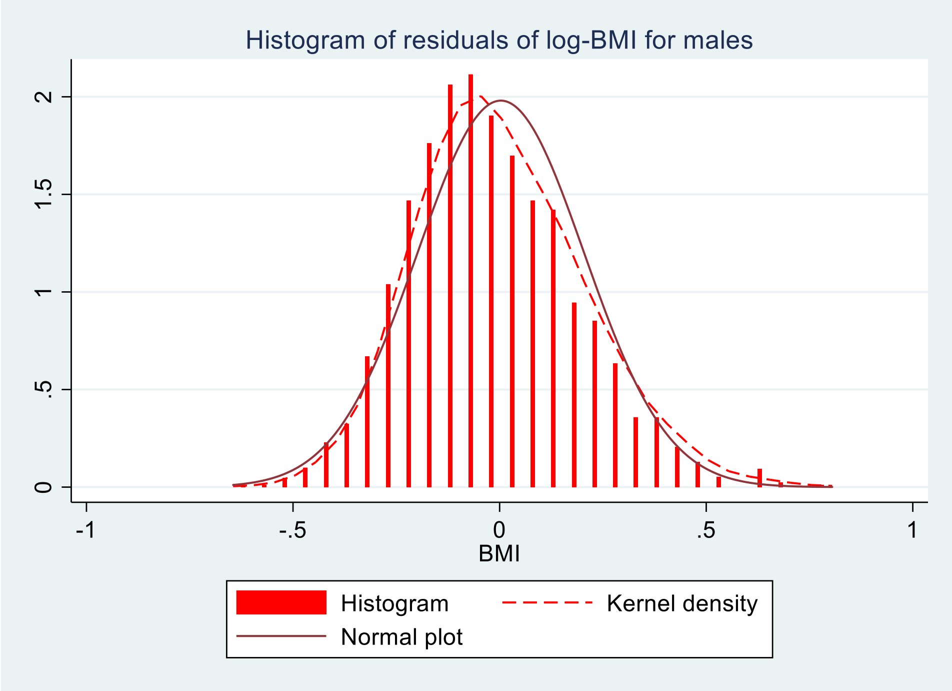

Appendix figure 4b: Histograms of residuals of between measured and estimated log-BMI for males to with kernel density and normal plot to examine for deviation from normality.

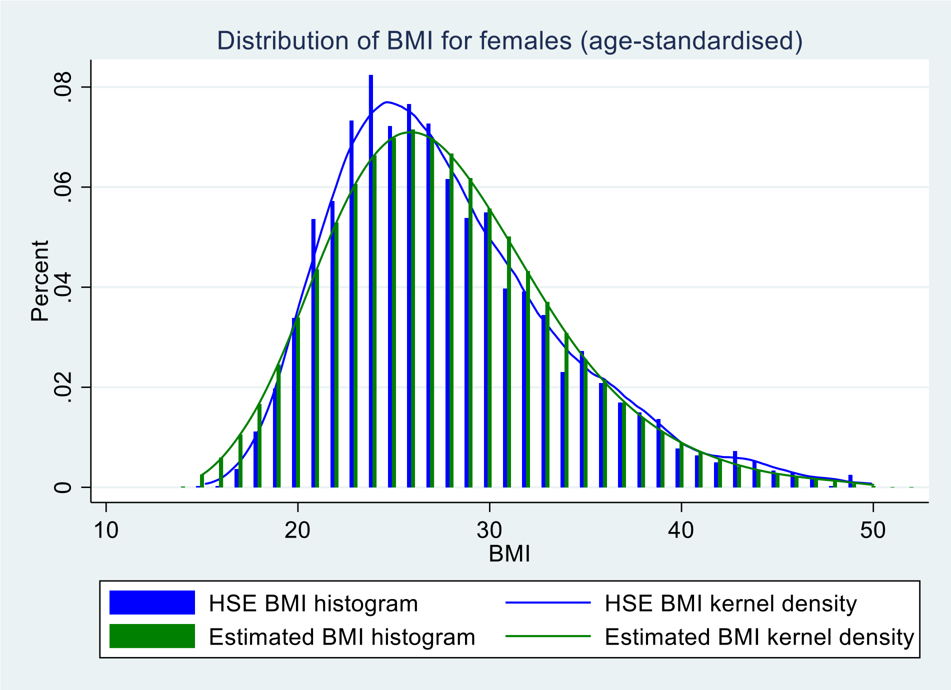

Appendix figure 5a: Histogram of age- and sex-standardised BMI estimates for England for females, including the equivalent histogram for the HSE 2018 BMI data for comparison.

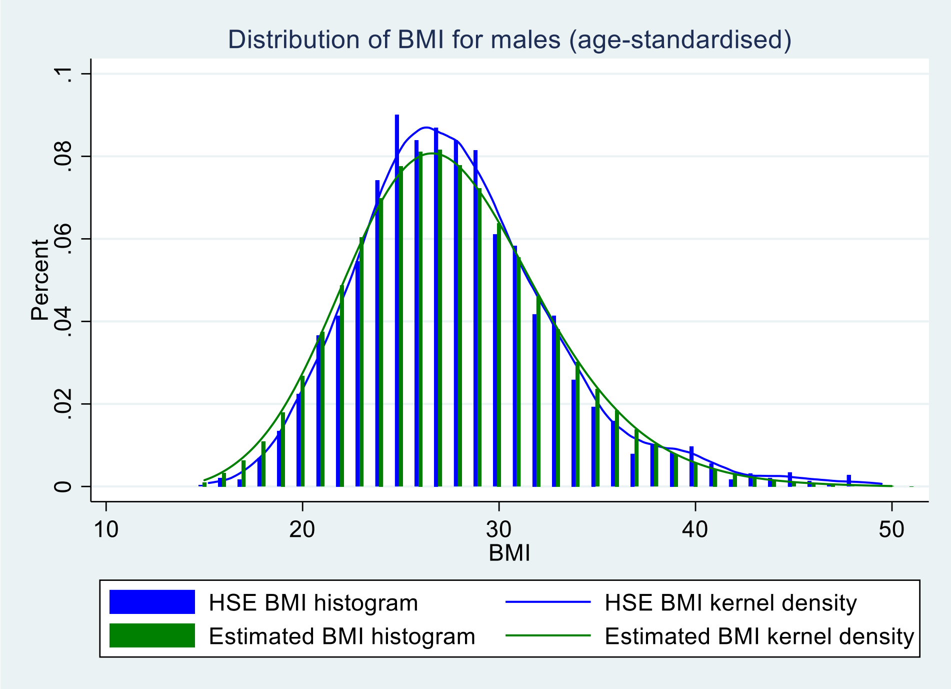

Appendix figure 5b: Histograms of age- and sex-standardised BMI estimates for England for males, including the equivalent histogram for the HSE 2018 BMI data for comparison.

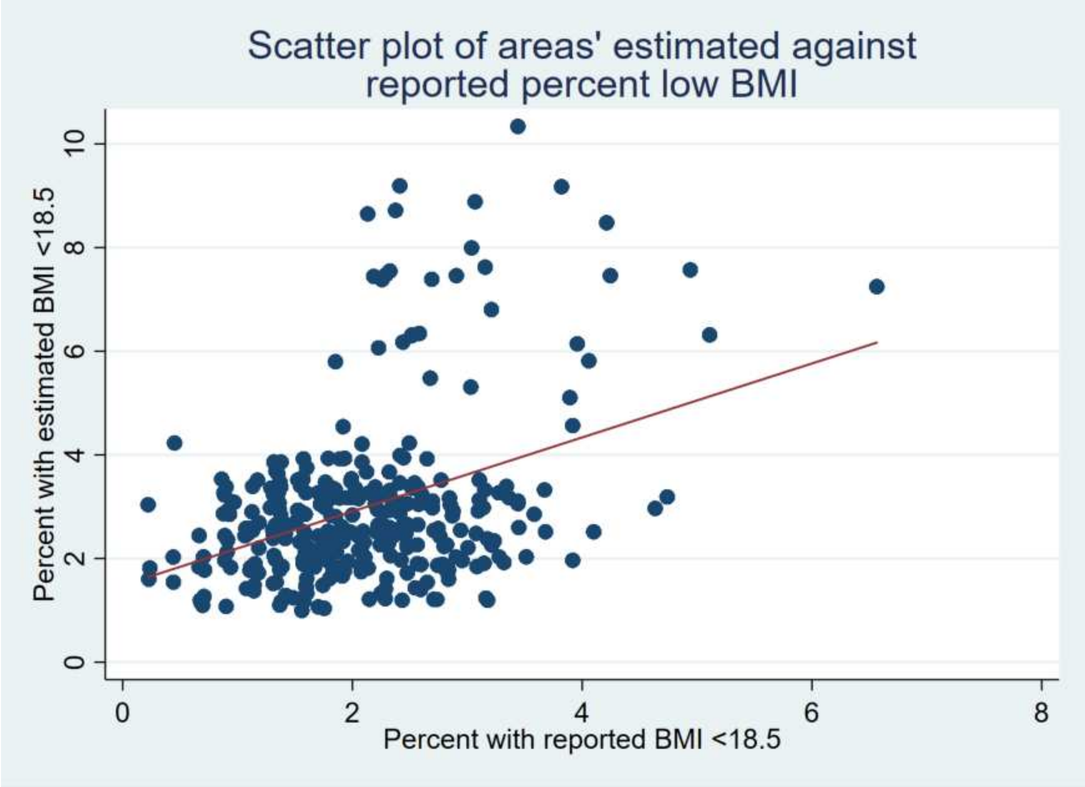

Appendix figure 6a: Scatter plot of the relationship between local areas' estimated versus reported percent of people with BMI <18.5.

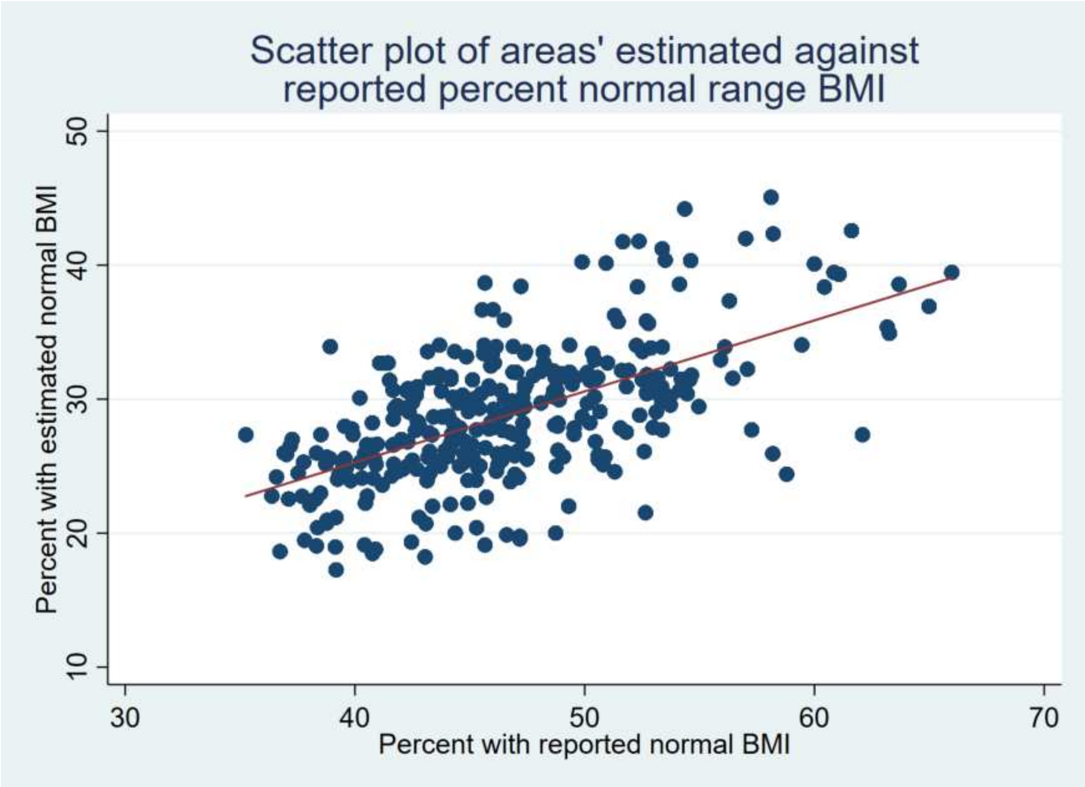

Appendix figure 6b: Scatter plot of the relationship between local areas' estimated versus reported percent of people with BMI in the normal range.

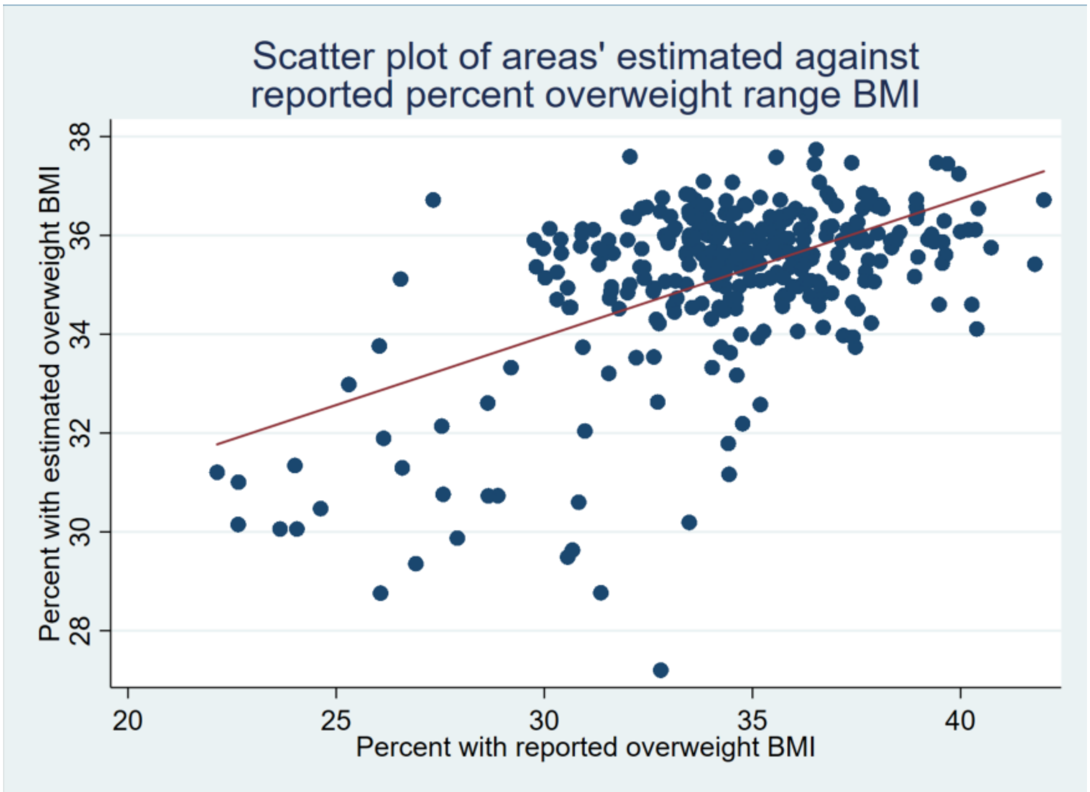

Appendix figure 6c: Scatter plot of the relationship between local areas' estimated versus reported percent of people with BMI in the overweight range.

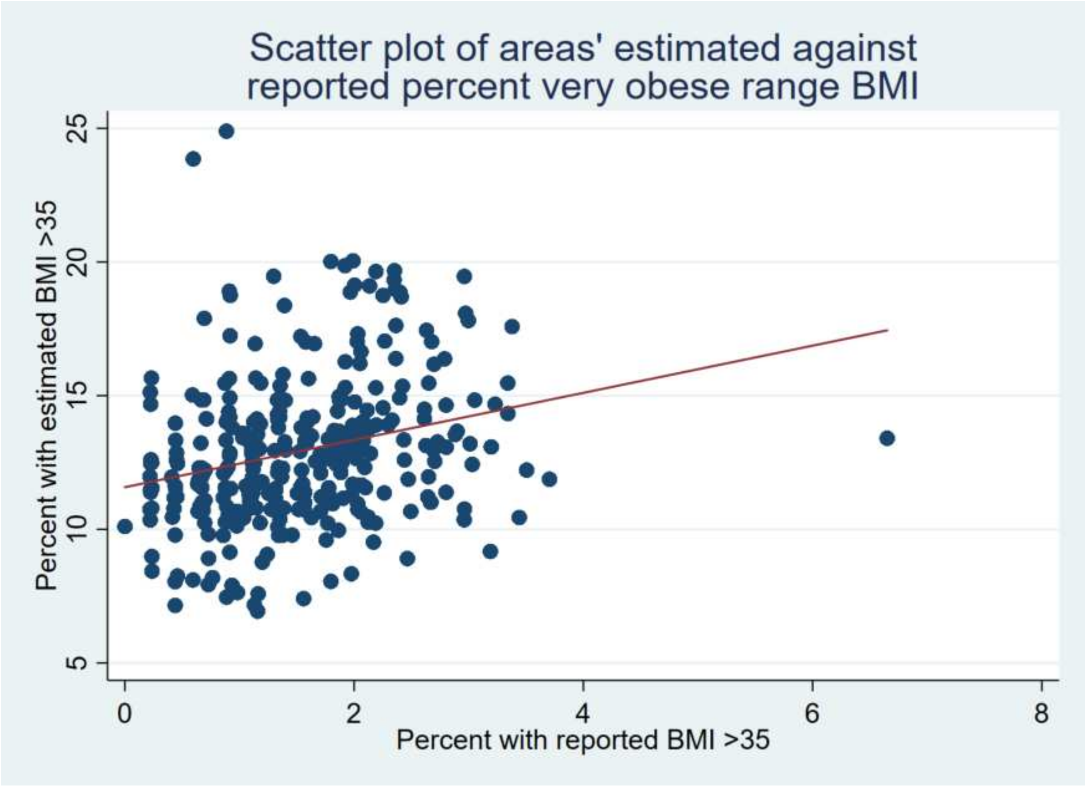

Appendix figure 6d: Scatter plot of the relationship between local areas' estimated versus reported percent of people with BMI >35.
