# Supplementary material for: Estimating BMI distributions by age and sex for local authorities in England: a small area estimation study
Source: BMJ Open. 2022 Jun 21;12(6):e060892. doi: 10.1136/bmjopen-2022-060892 (PMC9226908; doi:10.1136/bmjopen-2022-060892)
Supplement: Author's manuscript [file bmjopen-2022-060892.draft_revisions.pdf]

BMJ Open is committed to open peer review. As part of this commitment we make the peer review history of every article we publish publicly available.

When an article is published we post the peer reviewers' comments and the authors' responses online. We also post the versions of the paper that were used during peer review. These are the versions that the peer review comments apply to.

The versions of the paper that follow are the versions that were submitted during the peer review process. They are not the versions of record or the final published versions. They should not be cited or distributed as the published version of this manuscript.

BMJ Open is an open access journal and the full, final, typeset and author-corrected version of record of the manuscript is available on our site with no access controls, subscription charges or pay-per-view fees (http://bmjopen.bmj.com).

If you have any questions on BMJ Open's open peer review process please email info.bmjopen@bmj.com

BMJ Open

Estimating BMI distributions by age and sex for local authorities in England

Journal:	*BMJ Open*
Manuscript ID	bmjopen-2022-060892
Article Type:	Original research
Date Submitted by the Author:	17-Jan-2022
Complete List of Authors:	Amies-Cull, Ben; University of Oxford, Nuffield Department of Population Health<br>Wolstenholme, Jane; University of Oxford, HERC, Nuffield Department of Population Health<br>Cobiac, Linda; Griffith University<br>Scarborough, Peter; University of Oxford, Nuffield Department of Population Health
Keywords:	EPIDEMIOLOGY, PUBLIC HEALTH, STATISTICS & RESEARCH METHODS

SCHOLARONE™
Manuscripts

I, the Submitting Author has the right to grant and does grant on behalf of all authors of the Work (as defined in the below author licence), an exclusive licence and/or a non-exclusive licence for contributions from authors who are: i) UK Crown employees; ii) where BMJ has agreed a CC-BY licence shall apply, and/or iii) in accordance with the terms applicable for US Federal Government officers or employees acting as part of their official duties; on a worldwide, perpetual, irrevocable, royalty-free basis to BMJ Publishing Group Ltd ("BMJ") its licensees and where the relevant Journal is co-owned by BMJ to the co-owners of the Journal, to publish the Work in this journal and any other BMJ products and to exploit all rights, as set out in our licence.

The Submitting Author accepts and understands that any supply made under these terms is made by BMJ to the Submitting Author unless you are acting as an employee on behalf of your employer or a postgraduate student of an affiliated institution which is paying any applicable article publishing charge ("APC") for Open Access articles. Where the Submitting Author wishes to make the Work available on an Open Access basis (and intends to pay the relevant APC), the terms of reuse of such Open Access shall be governed by a Creative Commons licence – details of these licences and which Creative Commons licence will apply to this Work are set out in our licence referred to above.

Other than as permitted in any relevant BMJ Author's Self Archiving Policies, I confirm this Work has not been accepted for publication elsewhere, is not being considered for publication elsewhere and does not duplicate material already published. I confirm all authors consent to publication of this Work and authorise the granting of this licence.

For peer review only

Title:

Estimating BMI distributions by age and sex for local authorities in England

Corresponding author:

Ben Amies-Cull

Big Data Institute

Nuffield Department of Population Health

University of Oxford

Oxford, UK

OX3 7LF

ben.amies-cull@ndph.ox.ac.uk

Tel (+44 1865) 617784

Fax (NA)

Jane Wolstenholme, Nuffield Department of Population Health, University of Oxford, Oxford, UK

Linda Cobiac, Griffith University, Gold Coast, Australia

Peter Scarborough, Nuffield Department of Population Health, University of Oxford, Oxford, UK

Keywords:

Public health, epidemiology, obesity,

Word count: 3026

For peer review only

Abstract

Objectives

Rates of overweight and obesity vary across England, but local rates have not been estimated for over 10 years. We aimed to produce new small area estimates of BMI by age and sex for each lower-tier and unitary local authority in England, to provide up-to-date and more detailed estimates for the use of policymakers and academics working in non-communicable disease risk and health inequalities.

Design

We used Generalised Linear Modelling to estimate the relationship between BMI with social/ demographic markers in a cross-sectional survey, then used this model to impute a BMI for each adult in locally-representative populations. These groups were then disaggregated by 5-year age group, sex and local authority group.

Setting

The Health Survey for England 2018 (cross-sectional BMI data for England) and Census microdata 2011 (locally representative).

Participants

A total of 6,174 complete cases aged 16 and over were included.

Outcome measures

Modelled group-level BMI as mean and standard deviation of log-BMI. Extensive internal validation was performed, against the original data and external validation against the National Diet and Nutrition Survey and Active Lives Survey and previous small area estimates.

Results

In 94% of age-sex-are groups mean BMI was in the overweight or obese ranges. Older and more deprived areas had the highest overweight and obesity rates, which were particularly in coastal areas, the West Midlands, Yorkshire and the Humber. Validation showed close concordance with previous estimates by local area and demographic groups.

Conclusion

This work updated previous estimates of the distribution of BMI in England and contributes considerable additional detail to our understanding of the local epidemiology of overweight and obesity. Raised BMI now affects the vast majority of demographic groups by age, sex and area in England, regardless of geography or deprivation.

Strengths and limitations of this study

- This study provides considerable additional detail to previous local area estimates of BMI in England by estimating distributions of BMI and disaggregating local areas by age and sex group
- Social and demographic heterogeneity is incorporated into analysis by using the Census microdata 2011 and GLM regression, allowing variation in exposure-effect relationships

For peer review only

- Extensive internal and external validation exercises demonstrate good concordance with expected findings by age, sex and local area
- Results required calibration, particularly affecting younger individuals in the microdata, to allow the distribution of BMI to closely reflect the base data
- It was not possible to estimate parameter uncertainty for these estimates

Estimating BMI distributions by age and sex for local authorities in England

Introduction

Good quality primary data on Body Mass Index (BMI) do not exist for local areas of England. The sample size and sampling structure of national health surveys generally prevent health variables from being estimated at the local level by simple disaggregation, so a wide variety of local area estimation techniques have been developed(1–3). These are largely designed to predict the prevalence of a categorical variable rather than a continuous distribution that is more appropriate for many variables such as BMI. These estimates are useful for policymakers at local and national levels to understand where and for who the burden of raised BMI is highest and where to direct scarce resources. They also provide a valuable input to further research into BMI-related risk and related health inequalities.

The most recent set of estimates for BMI at the local authority level in England are by Moon *et al* (2007)(4) based on data from 1998-99. Their paper used multilevel modelling to estimate the proportions of each local authority in the overweight and obese BMI categories. This found obesity rates were highest in parts of the West Midlands, Yorkshire and the Humber, and overweight rates highest in coastal areas, particularly in Devon and Cornwall.

Survey data exist for estimating risk factor prevalence in England from studies such as the Health Survey for England (HSE)(5), National Diet and Nutrition Survey (NDNS)(6) and Active Lives Survey (ALS)(7), but each of these has limitations that prevent them from giving precise estimates of risk factor prevalence for age and sex groups at the local authority level. Both the HSE and NDNS are underpowered for such estimates and they do not report each respondent's local authority. The ALS collects self-reported height and weight data from phone interview and reports data at the local authority level, but only reports BMI as BMI category (eg. overweight, obese). These surveys suffer with the common problems for small area estimation(8) that they do not specify the local geographic area of the respondent and their sample sizes are too small to deliver precise estimates of risk factors for subgroups. To overcome these problems with survey data, a wide variety of small area estimation methods have been developed to allow researchers to estimate the prevalence of risk factors for small geographical areas. These methods can be grouped into three broad approaches(1–3): indirect standardisation, model-based estimation and microsimulation approaches. The indirect standardisation approach involves calculating the proportion of the population with a feature of interest (eg. BMI in the overweight range) for different subgroups, then weights these proportions according to the subgroups' representation in each local area. Model-based estimation improves on this approach by using logistic regression (usually) to estimate the proportion of a feature in a local area based on individual-level and area-level variables, using

For peer review only - http://bmjopen.bmj.com/site/about/guidelines.xhtml

aggregate data on the frequency of these variables(1,2). This approach was improved on by using Census data to increase the number of variables available for use.(9) To better account for unmeasured variation between areas, for example related to more granular sociodemographics, local context and culture(10), Twigg *et al*(11) developed an approach using multilevel logistic regression (later used for the BMI estimates by Moon *et al* 2007,(4) mentioned above). Finally, microsimulation approaches combine spatially aggregated survey data with geographically disaggregated microdata (that may need to be simulated)(8) but estimating credible intervals is challenging.(12,13)

We set out to estimate and validate the continuous distribution of BMI by age and sex in each local authority in England. We built on previous approaches to estimate continuous distributions, combining the strengths of model-based estimates and microsimulation.

Methods

Producing the modelled estimates of BMI distributions involved the following steps. First, we selected appropriate national-level survey data that included a BMI variable. Then we identified social and demographic variables that existed in both the health survey and the Census microdata 2011 (comprising every 20th entry from the UK Census 2011, available for all local authorities in England). We then fit a model relating these variables with BMI on the health survey data and applied the model to each respondent in the Census microdata, to generate predictions of BMI at the individual level. These estimates were validated using established approaches(12,14).

Data

For the national-level survey data on BMI, the most recent wave of HSE (2018)(5) was chosen as the BMI dataset on the basis that the alternative of the NDNS 2014-16 has a much smaller sample size than the HSE(6) and the ALS 2017 has only a self-reported categorical BMI variable.(7)

The local authority-level data were taken from the Census microdata 2011, which is a 5% sample of anonymised individual-level Census records, with the lowest geographic level at the lower tier or unitary local authority (UK local government, mostly 150,000-500,000 persons, of which there are 315 in England, plus the Isles of Scilly and City of London), or small groups of these termed "local authority Census groups" where populations are small.

'Candidate variables' for the model were social or demographic markers that were available in the same or translatable forms in the HSE 2018 and the Census microdata. These were age, sex, ethnicity, self-rated health (from 1 (good) to 5 (poor)), number of cars in the household, socioeconomic status (SES, using the National Statistics Socioeconomic Classification), NUTS-1 region (the nine regions of England), tenure status (ie. living arrangements, such as renters), and dummy variables for being unemployed, being a student, having no formal qualifications, and having a degree. Index of Multiple Deprivation (IMD) is given in the HSE at the postcode level, but there is no IMD variable given in the Census microdata, so it had to be merged at the local authority level from an external dataset.(15) Appendix table 1 provides a full description of these candidate variables and adaptations to make them equivalent. Participants in the HSE with missing data either for BMI or any of the candidate variables were dropped, detailed in Appendix tables 2 and 3.

Generalised Linear Models

For peer review only

The estimation process involved building a Generalised Linear Model (GLM) regression model in HSE 2018 with BMI as the dependent variable and the candidate variables as independent variables. This model was then used to predict a BMI for each individual in the UK Census 2011 microdata. To specify the GLM model, the data were first examined. The variance function (or family) was chosen on the basis of examination of the BMI variable, which identified a continuous skewed bell-shaped distribution, consistent with either a gamma or a gaussian (including log-normal) mean-variance relationship. Formal tests for family choice such as the modified Park test(16) could not be used as they rely on squared error and are not valid with clustered sampling. The Cullen-Frey plot and QQ-plot in Appendix figures 1 and 2, respectively, indicate the BMI variable conformed more closely with a lognormal than gamma distribution. Therefore, the GLM was built specifying a gaussian family with log link, as has become accepted practice(16,17) for log-normally distributed dependent variables. Cluster robust standard errors were specified to account for clustered sampling in HSE.

The model was built by regressing each variable against BMI for individuals aged 16 and over and including it in the model if Bayesian Inference Criterion (BIC) indicated an improved model (with an arbitrary cutoff of -2 to indicate superiority). BIC was used over the Akaike Information Criterion as BIC prefers more parsimonious models. Likelihood Ratio tests were not used as they are invalid with robust standard errors.(16) Ordinal variables were assessed as continuous before assessing if the levels contributed beyond linearity. If the final model output Wald Test did not support a significant association (at p<= 0.05), the variable's contribution was re-assessed by BIC.

Outputs were used to impute BMI in the Census microdata for each respondent aged 16 and over. GLM log-link variables are given in the log scale and interpreted multiplicatively as:

$$E(Y) = \prod_{i=0}^{n} \exp(\beta_i X_i)$$

Where E(Y) is the estimate of the independent variable (BMI), X_i are variable values (where $X_0 = 1$ for the constant) and β_i are coefficients. To reflect the uncertainty around coefficient estimates, random variation was added to coefficients at the individual level. This random variable was normally distributed with standard deviation equal to the standard error of the coefficient from the GLM. Variance of BMI was underestimated in the first set of estimates so these were calibrated. To do this, individual-level estimates in each age-sex group were transformed by subtracting the mean of the group and dividing by the SD of that group, before retransforming by multiplying them by a smoothed estimate of that group's SD in the HSE data and adding back the mean of the group. Mean and SD of estimated log-BMI were then taken for each age-sex-area group.

Validation steps

Three validation steps were used, based on previous approaches.(12,14)

1. Estimates were assessed for heteroskedasticity by examining residual plots of log-BMI. Formal tests for heteroskedasticity (eg. Breush-Pagan test) are not valid with robust standard errors.
2. Internal validity was tested by comparing age-standardised distributions of estimated and measured BMI (from HSE 2018) by sex.
3. External validity was tested in three ways.
   - Comparing age-standardised distributions of estimated and measured BMI from NDNS 2014-16 by sex

- For each local authority, the estimated obesity rate was plotted against the obesity rate according to self-reported BMI from the ALS 2017.
- Results were compared with Moon *et al* 2007(4), in terms of the areas in the top decile for obesity rates and for overweight rates.

Public and patient involvement statement

Patients or the public were not involved in the design, or conduct, or reporting, or dissemination plans of our research.

Ethics

The terms of the institutional ethics committees were reviewed. The study did not require ethics approval as only pre-collected data on non-identifiable individuals was used.

Results

BMI was estimated for adult males and females across 16 age groups in 315 local authority areas of England. In 94% of these subgroups the mean BMI was in the overweight range (97% for males, 90% for females). In no area was mean BMI in the normal range between the ages of 50 and 85 years for either sex. Appendix table 4 shows that people with raised BMI in HSE 2018 tended to be older and more deprived and had worse health. There were 6174 complete cases in HSE and Appendix table 2 shows details of missing data, identifying 18% missing for the BMI variable and Appendix table 3 shows that older people are overrepresented in these missing data.

Model results:

Table 1 shows the coefficients for each of the variables fitted in the final model, with their 95% confidence intervals.

Table 1: Model variables, coefficients and coefficient 95% confidence intervals given to 3 significant figures (* = interaction between stated variables). IMD = Index of Multiple Deprivation, SES = socioeconomic status.

Variable	Coefficient (log scale)	95% confidence intervals	
		Lower	Upper
Age (years)	0.0114	0.00932	0.0136
Age (years) squared	-9.81x10^{-05}	-0.000119	-7.70 x10^{-5}
Female (dummy)	0.108	0.0288	0.188
Female*age	-0.00398	-0.00709	-0.000874
Female*age squared	3.22 x10^{-5}	3.66 x10^{-6}	6.08 x10^{-5}

For peer review only

Ethnicity			
White/ mixed (base)			
Black	0.0483	0.0147	0.0819
Asian	-0.0219	-0.0435	-0.000323
Other	-0.0423	-0.0901	0.00538
Self-employed (dummy)	-0.0159	-0.0337	0.00200
Retired (dummy)	0.00450	-0.0154	0.0244
Long-term sick (dummy)	-0.0221	-0.0653	0.0210
No qualifications (dummy)	-0.00621	-0.0209	0.00844
Degree holder (dummy)	-0.0286	-0.0407	-0.0164
IMD			
Quintile 1 (base, lowest deprivation)			
Quintile 2	0.00732	-0.00890	0.0235
Quintile 3	0.0130	-0.00446	0.0305
Quintile 4	0.0176	0.000301	0.0349
Quintile 5	0.0315	0.0116	0.0514
Health status			
Very good (base)			
Good	0.0574	0.0467	0.0682
Fair	0.108	0.09207	0.124
Bad	0.109	0.0794	0.138
Very bad	0.142	0.0838	0.200
Tenure status			
Own outright (base)			
On a mortgage	-0.00130	-0.0178	0.0152
Shared ownership	0.0275	-0.0525	0.107
Rental	0.0192	0.00304	0.0354
Living rent-free	0.0684	-0.000499	0.137
Region			
North East (base)			
North West	-0.00122	-0.0249	0.0225
Yorks and Humber	0.0209	-0.00582	0.0477
East Midlands	0.00591	-0.0194	0.0312
West Midlands	0.0202	-0.00633	0.0467
East of England	0.0122	-0.0140	0.0384
London	-0.0289	-0.0540	-0.00373
South East	0.0162	-0.00975	0.0423
South West	-0.00181	-0.0259	0.0222
SES			
AB – managerial and professional (base)			
C1 – intermediate occupations	0.00200	-0.0177	0.0217
C2 – Small employers and own account workers	0.0141	-0.0113	0.0395
DE – lower supervisory, technical, and semi-routine occupations	-0.0116	-0.0280	0.00480
Other/ no response	-0.0123	-0.0366	0.0120
Constant	2.97	2.91	3.03

For peer review only

After standardising for age, mean BMI was 27.7kg/m^2 for females and 27.9kg/m^2 for males, though the distribution was broader for females, with higher proportions in low and high categories. Figure 1 shows the orders of magnitude of difference between local authorities across different quintiles of deprivation with the England average for reference. Even for the lowest deprivation area, almost all age-sex groups have mean BMI in the overweight range.

Validation

Heteroskedasticity

Residual plots (Appendix figure 3) demonstrated homoskedasticity. Trend lines show no overall bias across measured log-BMI and residuals' are shown to be normally distributed in Appendix figures 4a and 4b.

Internal validity

The age-standardised England-level distributions of estimated BMI for females and males are compared with the equivalent distributions of measured BMI from the original HSE 2018 data, shown in Appendix figures 5a and 5b, showing close concordance with the base data.

Figure 2 compares the 25th, 50th and 75th centiles of the calibrated and uncalibrated estimates with the HSE data across the age range across England. These show that calibration mainly affected younger age groups and produced distributions closely in alignment with those in the HSE.

External validity

Estimated BMI distributions for females and males were compared with the equivalent distributions from the independently-collected NDNS 2014-16 data in figures 3a and 3b (standardised to represent each age group equally). Figure 4 plots the reported proportion of people with BMI in the obese range for each local authority in the ALS against the same proportions on the new estimates. This shows a close relationship, though the proportion in the obese range is approximately 5 percentage points higher in the estimates than ALS across the distribution. Appendix figures 6a-6d show graphs for the other BMI categories.

Comparison of model results with those by Moon *et al* (2007)(4) shown in figure 5. As with Moon *et al*, many of the areas with highest estimated obesity rates were in the Yorkshire and the Humber, while overweight rates were highest in coastal areas. Coefficients used in their model were similar to this paper: individual-level age, sex, Black ethnicity, Asian ethnicity, and area-level percentages of low social grade, high social grade, households with dependent children, in local authority housing, and adults married. Likewise, their greatest coefficients were with age and sex, but Black and Asian ethnicities were more important in their paper while health status more.

Discussion

For peer review only - http://bmjopen.bmj.com/site/about/guidelines.xhtml

This paper introduces a method that combined the individual-level associations of BMI with social and demographic features to estimate the BMI of each individual in the Census microdata 2011, allowing a mean BMI and associated distribution to be estimated for each five-year age and sex group for each local authority. It builds on the established methods estimating local BMI(1,4,9,17) while adding further detail by estimating continuous distributions of BMI. These will provide policymakers and academics much greater detail on BMI-related risk, allowing policy impact on health inequalities to be better understood and enabling resources to be better targeted.

The distributions of age- and sex-level estimates were calibrated to replicate the national distribution of BMI. Figure 2 shows that this process produced highly consistent distributions with the underlying data and calibration affected younger ages groups more. Females had higher proportions with low and high BMI. BMIs were higher in older and more deprived areas.

Examination of residuals identified homoskedasticity (Appendix Figure 2), in the context of the GLM implying that the link function was appropriately specified and that the model was well-fitted. Internal validation against HSE showed good concordance. External validation at the local authority level against the ALS in Figure 4 showed a close linear relationship between the proportion of the population in the obese range, though with a distribution approximately 5 percentage points higher in the estimates. This could be due to the reporting bias of the self-reported height and weight items in the ALS or response bias of people with lower BMI into the survey. The other internal and external validation exercises indicate that this is unlikely to be a consequence of bias in the estimates.

Comparison of the new estimates against those from Moon *et al* 2007(4) show close alignment. Many of the same local areas are identified, such as Birmingham, Humberside, Lincolnshire and the south coast. In others, if the same area was not identified then neighbouring areas usually were, such as around Norwich.

Strengths and limitations

This novel approach of estimating BMI at the individual level has allowed estimates to be drawn with a greater degree of detail than previously, namely estimating the whole BMI distribution and drawing estimates for many subgroups. The HSE and Census microdata are both highly granular and representative of the population and data quality of the HSE is generally good, being a large and representative sample. One relevant limitation to the HSE 2018 is missing data, namely for the BMI variable, especially from the late 70s (Appendix table 3). One potential reason that older people are underrepresented may be poor health. While it is not possible to account for systematic differences in non-responders, health is captured in the model, while the Census should provide a representative estimate of health in each age group, helping to avoid bias from these missing data.

The range of variables that can be aligned between datasets is also a limitation, in common with previous local area estimation methods(11). First, the levels of geography do not align apart from at the NUTS-1 level. This means that multilevel modelling previously used(4,11,21,22) is not feasible, as the NUTS-1 areas are far too large and internally heterogeneous to pick up local authority-sized variation. Therefore, this method assumes that the national-level associations between the predictors and BMI do not systematically vary between local areas. Second, deprivation is measured at the postcode level in the HSE 2018 but is merged at the local authority level into the Census data. This means that deprivation is not heterogeneous within local authorities in the Census as it is in the real world, so resulting in slightly narrower modelled BMI. A calibration step was used to allow

estimated distributions to closely reflect the real world, relying on an assumption that the order of age-sex-area-level estimates produced by the model was correct.

The Census data is, of writing, ten years old. Ageing populations are not directly important as age is accounted for, but local changes (such as increasing education levels) may lead to bias in estimates. As the model was built using recent HSE and IMD data, change in association between variables and BMI over time should not be a source of bias. The final limitation is that estimating the parameter uncertainty to a variable's estimated mean in small area estimation methods is difficult(8) and not attempted here.

Conclusion

This study used HSE 2018 to estimate the BMI of respondents in the Census microdata 2011, providing the most recent set of disaggregated small area estimates for BMI at the local authority level in England since 2007(4). Granular social and demographic information provided by the Census microdata 2011 captured local variation. Results showed that over 50% of people in almost every demographic group and area had above-normal BMI.

Validation showed there was a low risk of heteroskedasticity and there were approximately normally-distributed residuals, providing confidence that models were well-fitted. Comparison of estimates against external datasets at the national and regional levels identified close concordance with previous findings, but comparison at the local authority level with self-reported BMI implied that many areas have higher rates of raised BMI than previously(20) estimated.

These new estimates go further than previous studies by two dimensions - by estimating BMI by age-sex groups and by modelling the distribution of BMI rather than percentages in BMI categories. As such, this study has produced the most demographically granular estimates for BMI in local areas of England to date.

Contributorship statement

BAC conceived of the study, contributed to study design, undertook the main analysis and completed the first draft of the manuscript.

JW conceived of the study, contributed to design, supervised the analysis and commented on drafts of the manuscript.

LC conceived of the study, contributed to design, supervised the analysis and commented on drafts of the manuscript.

PS conceived of the study, contributed to design, supervised the analysis and commented on drafts of the manuscript.

Competing interest statement

BAC is supported by the MRC, JW by the NIHR and CRUK, and PS by the BHF and NIHR Oxford Biomedical Research Centre.

For peer review only

Funders had no involvement in the study design; in the collection, analysis and interpretation of the data; in the writing of the report; or in the decision to submit the paper for publication.

Funding statement

This work was funded by the Medical Research Council grant number MR/N013468/1.

Data sharing statement

Results are available at Oxford Research Archive, provided as mean and SD of log-BMI for each age and sex group for each local authority area.

For peer review only

References

1. Scholes S, Bajekal M. Synthetic estimation of healthy lifestyles indicators: User guide [Internet]. Available from: https://www.researchgate.net/publication/265658543

2. Marshall A, Economic and Social Data Service. Small area estimation using ESDS government surveys – An introductory guide. 2010.

3. Rahman A. Estimating small area health-related characteristics of populations: A methodological review. Geospatial Health. 2017;12(1).

4. Moon G, Quarendon G, Barnard S, Twigg L, Blyth B. Fat nation: Deciphering the distinctive geographies of obesity in England. Social Science and Medicine. 2007 Jul;65(1):20–31.

5. National Centre for Social Research (NatCen), University College London, Department of Epidemiology and Public Health. (2020). Health Survey for England, 2018. [data collection]. UK Data Service. SN: 8649, http://doi.org/10.5255/UKDA-SN-8649-1.

6. University of Cambridge, MRC Epidemiology Unit, NatCen Social Research. (2021). National Diet and Nutrition Survey Years 1-11, 2008-2019. [data collection]. 19th Edition. UK Data Service. SN: 6533, http://doi.org/10.5255/UKDA-SN-6533-19.

7. Sport England. (2020). Active Lives Survey, 2017-2018. [data collection]. UK Data Service. SN: 8651, http://doi.org/10.5255/UKDA-SN-8651-1.

8. Rahman A, Harding A. Small area estimation and microsimulation modeling. Boca Raton; 2019.

9. Charlton J. Use of the Census Samples of Anonymised Records (SARs) and survey data in combination to obtain estimates at local authority level. Vol. 30, Environment and Planning A. 1998.

10. Macintyre S, Ellaway A, Cummins S. Place effects on health: how can we conceptualise, operationalise and measure them? Vol. 55, Social Science & Medicine. 2002.

11. Twigg L, Moon G, Jones K. Predicting small-area health-related behaviour: a comparison of smoking and drinking indicators [Internet]. 2000. Available from: www.elsevier.com/locate/socscimed

12. Edwards KL, Clarke GP, Thomas J, Forman D. Internal and External Validation of Spatial Microsimulation Models: Small Area Estimates of Adult Obesity. Applied Spatial Analysis and Policy. 2011 Nov;4(4):281–300.

13. Whitworth A, Aitken G, Mclennan D, Taylor J. Evaluations and improvements in small area estimation methodologies, National Centre for Research Methods Methodological Review paper. 2013.

14. Scarborough P, Allender S, Rayner M, Goldacre M. Validation of model-based estimates (synthetic estimates) of the prevalence of risk factors for coronary heart disease for wards in England. Health and Place. 2009;15(2):596–605.

For peer review only

15. English Indices of Deprivation 2019, Department for Communities and Local Government, Crown Copyright 2019, https://www.gov.uk/government/statistics/english-indices-of-deprivation-2019.

16. Partha D, Norton. Edward, Manning W. Health Econometrics Using Stata. College Station, Texas, 2017.

17. Mohammad N, Mcgivern L. Comparing Gamma and Log-Normal GLMs in R Using Simulation and Real Data Set.

18. Mansournia MA, Nazemipour M, Naimi AI, Collins GS, Campbell MJ. Reflection on modern methods: Demystifying robust standard errors for epidemiologists. International Journal of Epidemiology. 2021 Feb 1;50(1):346–51.

19. Edwards KL, Clarke GP. The design and validation of a spatial microsimulation model of obesogenic environments for children in Leeds, UK: SimObesity. Social Science and Medicine. 2009 Oct;69(7):1127–34.

20. Obesity Profile, Public Health England Public Health Profiles 2021, https://fingertips.phe.org.uk/profile/national-child-measurement-programme, Crown copyright 2021.

21. Moon G, Twigg L, Jones K, Aitken G, Taylor J. The utility of geodemographic indicators in small area estimates of limiting long-term illness. Social Science and Medicine. 2019 Apr 1;227:47–55.

22. Whitworth A, Carter E, Ballas D, Moon G. Estimating uncertainty in spatial microsimulation approaches to small area estimation: A new approach to solving an old problem. Computers, Environment and Urban Systems. 2017 May 1;63:50–7.

Figure legend

Figure 1a/b: Distribution of BMI across the age range for three local authorities for females (1a, above) and males (1b, below): Manchester (IMD quintile 5 – highest deprivation), Oxford (IMD quintile 3) and South Gloucestershire (IMD quintile 1)

Figure 2: Scatter plot of mean measured BMI, uncalibrated estimate and calibrated estimate, with their associated 25th-75th centile range across the age range (for both sexes combined).

Figure 3a/b: Histogram of age-standardised BMI estimates for England for females (3a, above) and males (3b, below), including the equivalent histogram for measured BMI from NDNS 2014-16 for comparison.

Figure 4: Scatter plot of local areas' age-standardised percent of population with estimated BMI in the obese range against self-reported BMI in obese range in ALS 2017, with best fit line.

Figure 5: Comparison of results from Moon et al 2007(4) (left) with new estimates (right), showing the top decile of areas with the highest rates of obesity (above) and overweight (below). Left images reprinted from Moon G et al, Fat nation: Deciphering the distinctive geographies of obesity in

For peer review only

England. Social Science and Medicine. 2007 Jul;65(1):20–31. Copyright (2007), with permission from Elsevier.

For peer review only

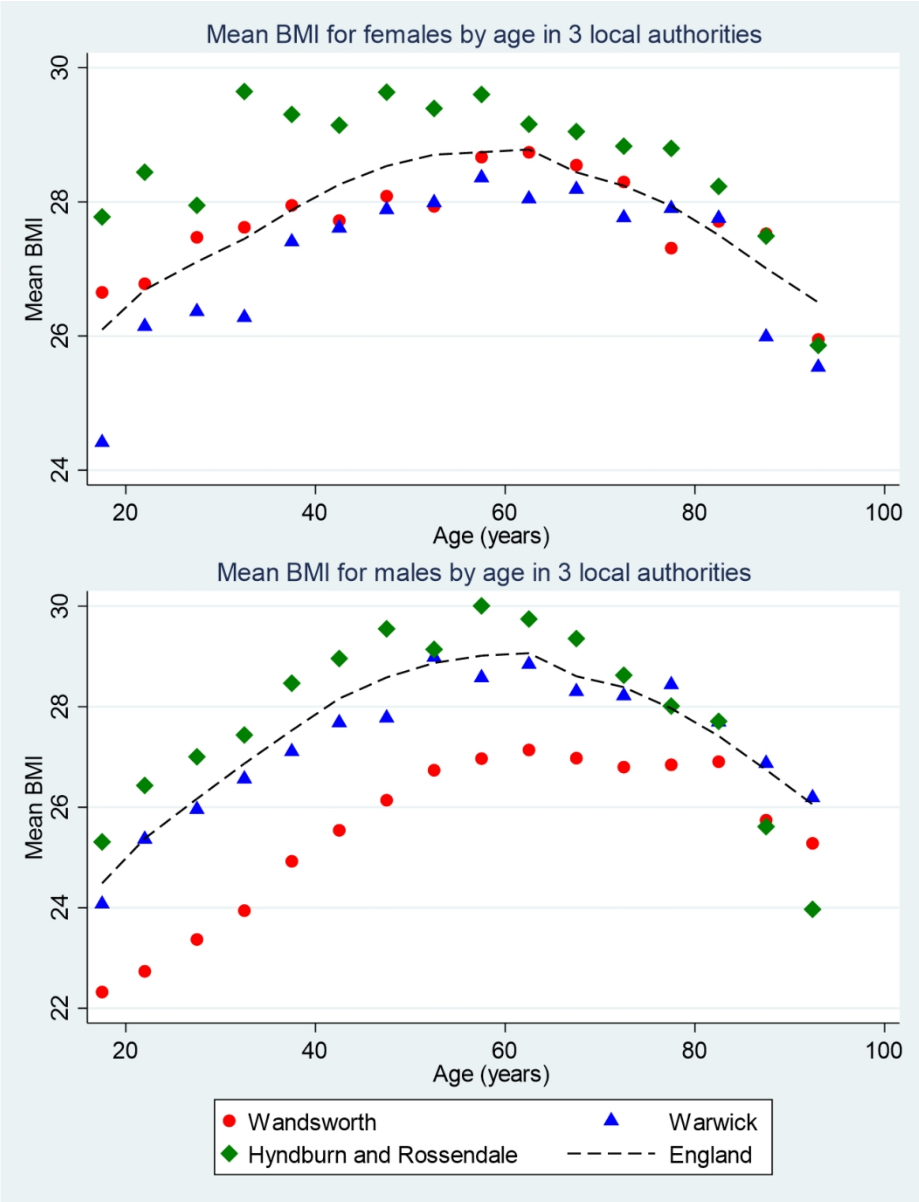

Figure 1a/b: Distribution of BMI across the age range for three local authorities for females (1a, above) and males (1b, below): Manchester (IMD quintile 5 – highest deprivation), Oxford (IMD quintile 3) and South Gloucestershire (IMD quintile 1)

387x506mm (85 x 85 DPI)

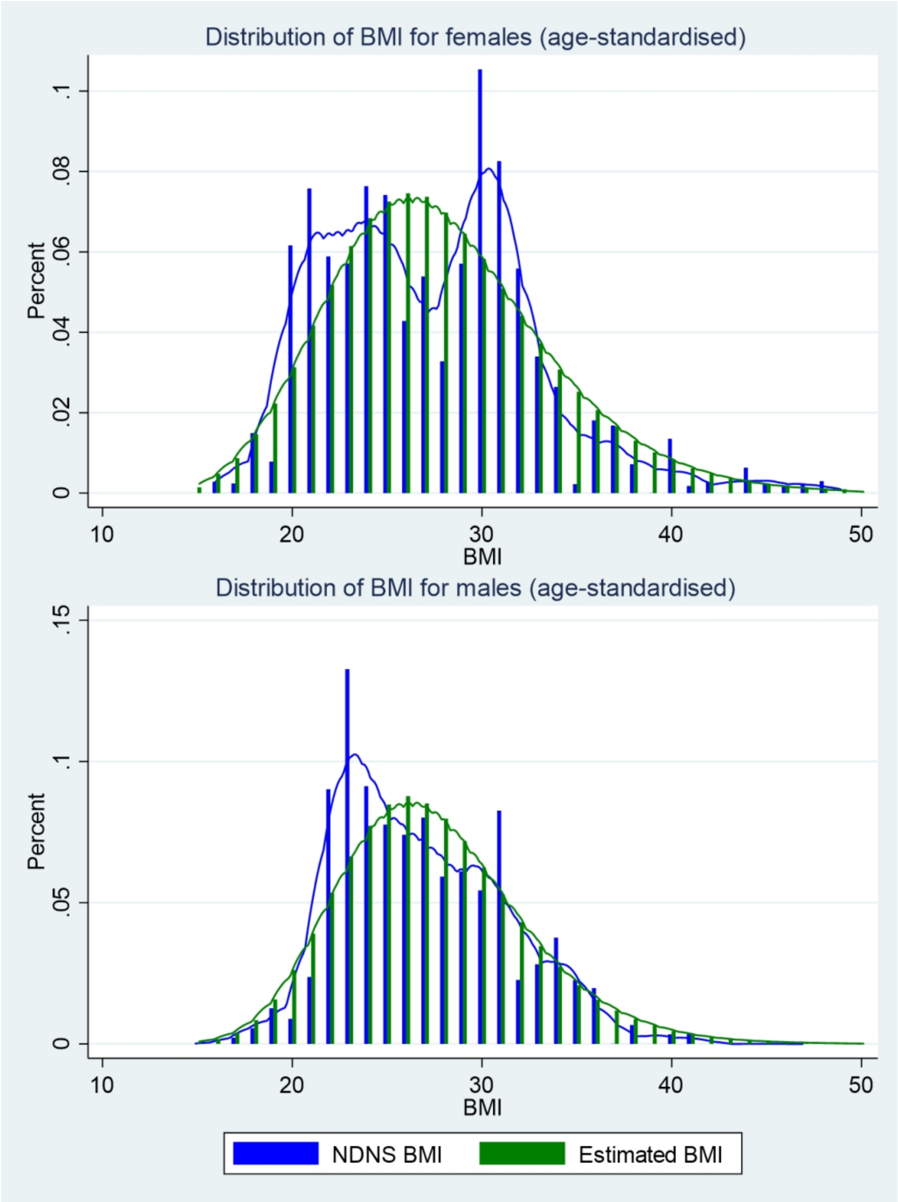

Figure 3a/b: Histogram of age-standardised BMI estimates for England for females (3a, above) and males (3b, below), including the equivalent histogram for measured BMI from NDNS 2014-16 for comparison.

388x519mm (85 x 85 DPI)

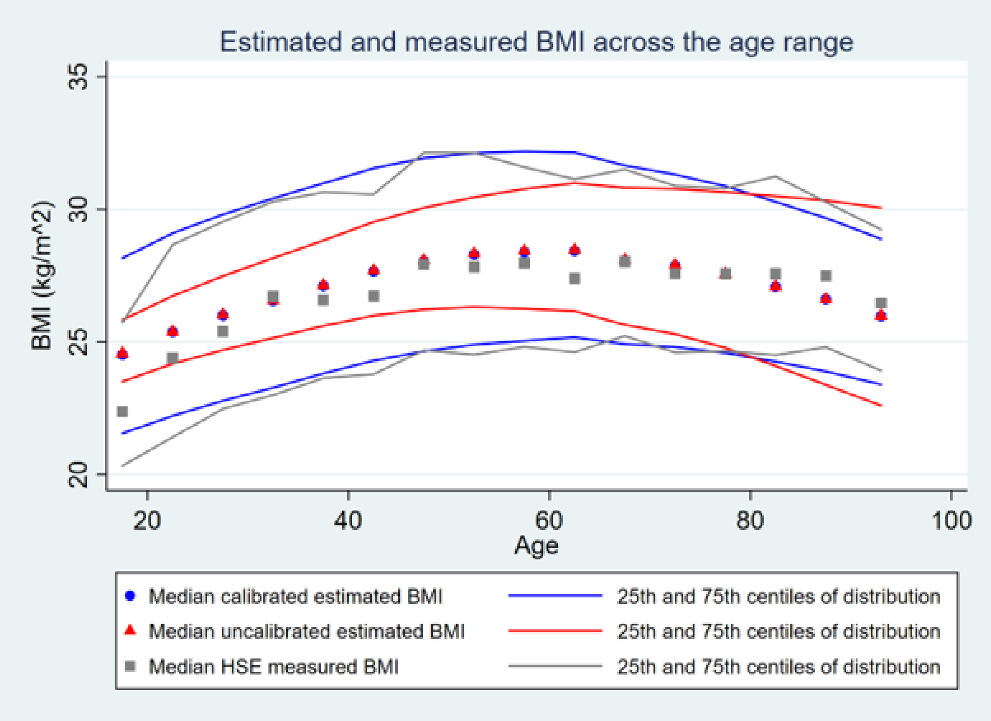

Figure 2: Scatter plot of mean measured BMI, uncalibrated estimate and calibrated estimate, with their associated 25th-75th centile range across the age range (for both sexes combined).

181x131mm (85 x 85 DPI)

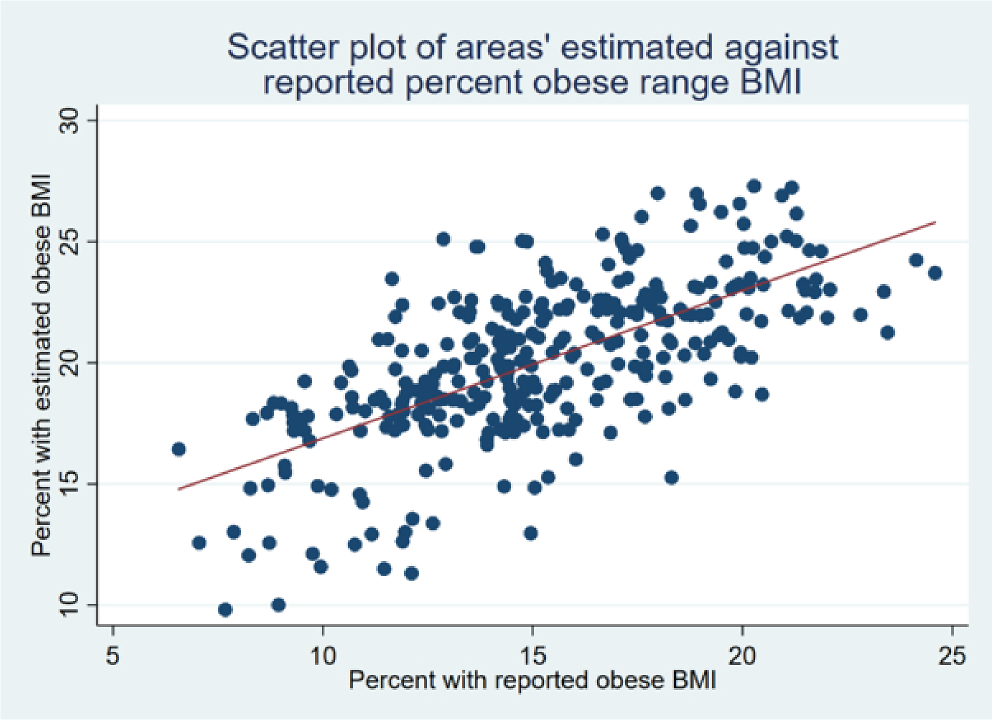

Figure 4: Scatter plot of local areas' age-standardised percent of population with estimated BMI in the obese range against self-reported BMI in obese range in ALS 2017, with best fit line.

181x130mm (85 x 85 DPI)

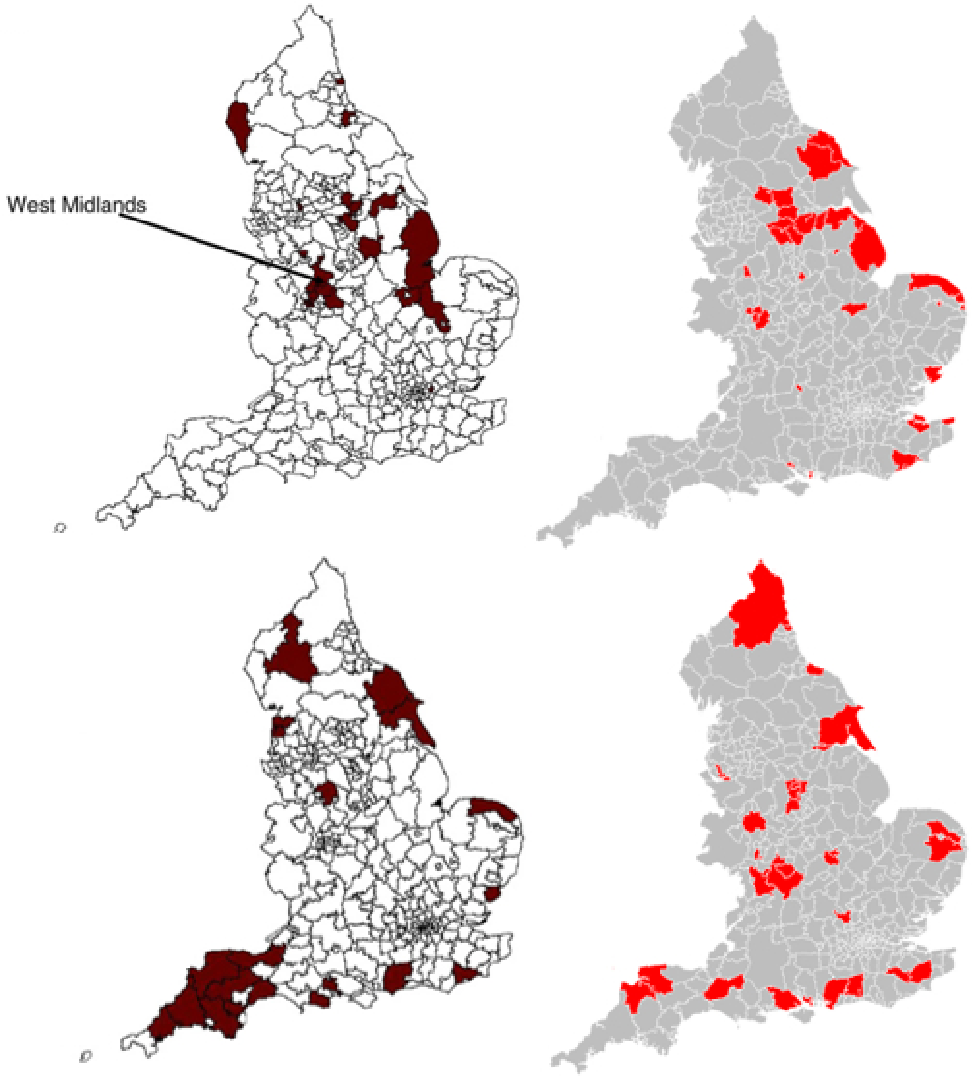

Figure 5: Comparison of results from Moon et al 2007(4) (left) with new estimates (right), showing the top decile of areas with the highest rates of obesity (above) and overweight (below). Left images reprinted from Moon G et al, Fat nation: Deciphering the distinctive geographies of obesity in England. Social Science and Medicine. 2007 Jul;65(1):20–31. Copyright (2007), with permission from Elsevier.

154x169mm (85 x 85 DPI)

Appendix tables

Appendix table 1: Variable mapping showing matched variables in HSE 2018 and the Census microdata and any adaptations to variables necessary to make them equivalent.

Characteristic	Census 2011 microdata Variable name	Census value	Census label	New value	New Label
Age	AGEC	1	0-4 year olds	(Missing)	
		2	5-9 year olds	(Missing)	
		3	10-15 year olds	(Missing)	
		4	16-18 year olds	17.5	16-18 year olds
		5	19-24 year olds	22	19-24 year olds
		6	25-29 year olds	27.5	25-29 year olds
		7	30-34 year olds	32.5	30-34 year olds
		8	35-39 year olds	37.5	35-39 year olds
		9	40-44 year olds	42.5	40-44 year olds
		10	45- 49 year olds	47.5	45- 49 year olds
		11	50- 54 year olds	52.5	50- 54 year olds
		12	55-59 year olds	57.5	55-59 year olds
		13	60-64 year olds	62.5	60-64 year olds
		14	65-69 year olds	67.5	65-69 year olds
		15	70-74 year olds	72.5	70-74 year olds
		16	75-79 year olds	77.5	75-79 year olds
		17	80-84 year olds	82.5	80-84 year olds
		18	85-89 year olds	87.5	85-89 year olds
		19	90+	F=93.0 M=92.4(1)	90+ year olds
Sex	SEX	1	male	0	male
		2	female	1	female
Ethnicity	AGGETHPUK113	1	White	1	White
		2	Mixed/multiple ethnic groups	4	Mixed/multiple
		3	Asian/Asian British: Indian	3	Asian
		4	Asian/Asian British: Pakistani	3	Asian
		5	Asian/Asian British: Bangladeshi	3	Asian
		6	Asian/Asian British: Chinese	3	Asian
		7	Asian/Asian British: Other Asian	3	Asian
		8	Black/African/Caribbean/Black British: African	2	Black

		9	Black/African/Caribbean/Black British: Black Caribbean	2	Black
		10	Black/African/Caribbean/Black British: Other Black	2	Black
		11	Other ethnic group	5	Any other group
**Unemployed**	**ECOPUK1**	1	Econ. Active (excl. Students), Employee, Part-time	0	Not unemployed
		2	Econ. Active (excl. Students), Employee, Full-time	0	Not unemployed
		3	Econ. Active (excl. Students), Self-employed with employees, Part-time	0	Not unemployed
		4	Econ. Active (excl. Students), Self-employed with employees, Full-time	0	Not unemployed
		5	Econ. Active (excl. Students), Self-employed no employees, Part-time	0	Not unemployed
		6	Econ. Active (excl. Students), Self-employed no employees, Full-time	0	Not unemployed
		7	Econ. Active (excl. Students), Unemployed(2)	1	Unemployed
		8	Economically Active Full-time Students, In Employment	0	Not unemployed
		9	Economically Active Full-time Students, Unemployed(2)	1	Unemployed
		10	Economically Inactive, Retired	0	Not unemployed
		11	Economically Inactive, Student	0	Not unemployed
		12	Economically Inactive, Looking after home/family	0	Not unemployed
		13	Economically Inactive, Permanently sick/disabled	0	Not unemployed
		14	Economically Inactive, Other	0	Not unemployed
**Student**	**ECOPUK1**	1	Econ. Active (excl. Students), Employee, Part-time	0	Not a student
		2	Econ. Active (excl. Students), Employee, Full-time	0	Not a student
		3	Econ. Active (excl. Students), Self-employed with employees, Part-time	0	Not a student
		4	Econ. Active (excl. Students), Self-employed with employees, Full-time	0	Not a student

		5	Econ. Active (excl. Students), Self-employed no employees, Part-time	0	Not a student
		6	Econ. Active (excl. Students), Self-employed no employees, Full-time	0	Not a student
		7	Econ. Active (excl. Students), Unemployed(2)	0	Not a student
		8	Economically Active Full-time Students, In Employment	1	Student
		9	Economically Active Full-time Students, Unemployed(2)	1	Student
		10	Economically Inactive, Retired	0	Not a student
		11	Economically Inactive, Student	1	Student
		12	Economically Inactive, Looking after home/family	0	Not a student
		13	Economically Inactive, Permanently sick/disabled	0	Not a student
		14	Economically Inactive, Other	0	Not a student
**Has a degree or equivalent**	**hlqupuk11**	10	No academic or professional qualifications	0	
		11	Level 1 (0-4 GCSE, O level, or equivalents)	0	
		12	Level 2 (5+ GCSE, O level, 1 A level, or equivalents)	0	
		13	Apprenticeship	0	
		14	Level 3 (2+ A levels, or equivalents)	0	
		15	Level 4+ (degree, postgrad, professional quals)	1	Has a degree or equivalent
		16	Other (vocational/foreign/outside UK quals)	0	
		-9	Not applicable	(Missing)	
**Self-reported general health**	**HEALTH**	1	Very good	1	Very good
		2	Good	2	Good
		3	Fair	3	Fair
		4	Bad	4	Bad
		5	Very bad	5	Very bad
**Deprivation**	**IMDQUINTE**	1	Least deprived	1	Least deprived
		2	Second least	2	Second least
		3	Intermediate	3	Intermediate
		4	Second most	4	Second most
		5	Most deprived	5	Most deprived
		-8	unknown	(Missing)	

	HSE 2017				
	Variable name	HSE value	HSE label	New value	New Label
Age	Age16g10	1	0-1 year olds	(Missing)	
		2	2-4 year olds	(Missing)	
		3	5-7 year olds	(Missing)	
		4	8-10 year olds	(Missing)	
		5	11-12 year olds	(Missing)	
		6	13-15 year olds	(Missing)	
		7	16-19 year olds	18	16-19 year olds
		8	20-24 year olds	22.5	20-24 year olds
		9	25-29 year olds	27.5	25-29 year olds
		10	30-34 year olds	32.5	30-34 year olds
		11	35-39 year olds	37.5	35-39 year olds
		12	40-44 year olds	42.5	40-44 year olds
		13	45- 49 year olds	47.5	45- 49 year olds
		14	50- 54 year olds	52.5	50- 54 year olds
		15	55-59 year olds	57.5	55-59 year olds
		16	60-64 year olds	62.5	60-64 year olds
		17	65-69 year olds	67.5	65-69 year olds
		18	70-74 year olds	72.5	70-74 year olds
		19	75-79 year olds	77.5	75-79 year olds
		20	80-84 year olds	82.5	80-84 year olds
		21	85-89 year olds	87.5	85-89 year olds
		22	90+	F=93.0, M=92.4(1)	90+ year olds
Sex	Sex	1	Male	0	male
		2	Female	1	female
		3	Refused	(Missing)	
		4	Don't know	(Missing)	
		5	Not applicable	(Missing)	
Ethnicity	Origin2	1	White	1	White
		2	Black	2	Black
		3	Asian	3	Asian
		4	Mixed/multiple ethnic background	4	Mixed/multiple
		5	Any other ethnic group	5	Any other group
		6	Refusal	(Missing)	

		7	Don't know	(Missing)	
**Unemployed**	**Activb2**	1	Going to school or college full-time	0	Not unemployed
		2	In paid employment or self-employed	0	Not unemployed
		3	Doing unpaid work for family business	0	Not unemployed
		4	Waiting to take up paid work already obtained	0	Not unemployed
		5	Looking for paid work/ Govt. training scheme	1	Unemployed
		6	Intending to look for work, but temporary sickness	0	Not unemployed
		7	Permanently unable to work, long-term sickness	0	Not unemployed
		8	Retired from paid work	0	Not unemployed
		9	Looking after home or family	0	Not unemployed
		95	Doing something else (specify)	0	Not unemployed
		-9	No answer/refused	(Missing)	
		-8	Don't know	(Missing)	
		-1	Not applicable	(Missing)	
**Student**	**Activb2**	1	Going to school or college full-time	1	Student
		2	In paid employment or self-employed	0	Not a student
		3	Doing unpaid work for family business	0	Not a student
		4	Waiting to take up paid work already obtained	0	Not a student
		5	Looking for paid work/ Govt. training scheme	0	Not a student
		6	Intending to look for work, but temporary sickness	0	Not a student
		7	Permanently unable to work, long-term sickness	0	Not a student
		8	Retired from paid work	0	Not a student
		9	Looking after home or family	0	Not a student
		95	Doing something else (specify)	0	Not a student
		-9	No answer/refused	(Missing)	
		-8	Don't know	(Missing)	
		-1	Not applicable	(Missing)	
**Has a degree or equivalent**	**topqual3**	1	NVQ4/NVQ5/Degree or equiv	1	Has a degree or equivalent

		2	Higher ed below degree	0		
		3	NVQ3/GCE A Level equiv	0		
		4	NVQ2/GCE O Level equiv	0		
		5	NVQ1/CSE other grade equiv	0		
		6	Foreign/other	0		
		7	No qualification	0		
		-9	Refused	(Missing)		
		-8	Don't know	(Missing)		
		-1	Not applicable	(Missing)		
**Self-reported general health**	**GenHelf**	1	Very good	1	Very good	
		2	Good	2	Good	
		3	Fair	3	Fair	
		4	Bad	4	Bad	
		5	Very bad	5	Very bad	
		-9	Refused	(Missing)		
		-8	Don't know	(Missing)		
		-1	Not applicable	(Missing)		
**Deprivation**	**(Merged from external dataset**(15)**)**	1	Least deprived - 0.48 ->8.37	1	Least deprived	
		2	Second least - 8.37 -> 13.92	2	Second least	
		3	Intermediate - 13.92- >21.43	3	Intermediate	
		4	Second most - 21.43 -> 33.88	4	Second most	
		5	Most deprived - 33.88 -> 92.60	5	Most deprived	

(1) Average age for over-90s in most recent ONS mid-year estimates of the England population is 93.0 years for females and 92.4 years for males.

(2) International Labour Organisation definition: seeking work and ready to start in 2 weeks, and Waiting to start a job already obtained and available to start within 2 weeks.

Appendix table 2: Percent of missing data in source datasets by variable used (England only, respondents aged 17 and over). *IMD was merged into Census data at the lower tier local authority level from ONS data.(15)

Dataset: Census microdata		HSE 2018	
Variable	% missing	Variable	% missing
age	0.00	age	0.00
sex	0.00	sex	0.00
ethnicity	1.28	ethnicity	0.39
student	1.28	student	0.21

has degree	1.28	has degree	0.46
health	1.28	health	0.04
No of cars	2.21	No of cars	0.06
IMD*	-	IMD	0.00
		BMI	18.33

Appendix table 3: Percent of missing data for the BMI variable used in HSE 2018 (BMIval2) by demographic group

Breakdown variable	Total records	Of which percent missing
Age		
18	281	17.8
22.5	366	19.4
27.5	487	17.5
32.5	581	18.1
37.5	647	14.2
42.5	684	15.8
47.5	668	15.3
52.5	686	17.9
57.5	733	16.2
62.5	634	17.5
67.5	614	14.3
72.5	625	20.0
77.5	444	22.3
82.5	298	30.5
87.5	177	36.2
90+	72	45.9
Sex		
Female	4461	18.7
Male	3536	17.8
IMD quintile		
1 (lowest deprivation)	1653	15.5
2	1707	18.3
3	1554	19.0
4	1557	19.1
5	1526	20.1

view only

Appendix table 4: Baseline population characteristics. Age given as mean (median, interquartile range). Remaining variables given as number (percent of total).

Number	BMI up to 25 N= 2,198	BMI over 25 N= 4,333	Total N= 6,531
Age (years)	42.5 (32.5,62.5)	52.5 (42.5,67.5)	52.5 (37.5,67.5)
Males	1,337 (60.8%)	2,289 (52.8%)	3,626 (55.5%)
Ethnicity			
White	1,916 (87.2%)	3,828 (88.5%)	5,744 (88.1%)
Black	47 (2.1%)	131 (3.0%)	178 (2.7%)
Asian	184 (8.4%)	288 (6.7%)	472 (7.2%)
Mixed	34 (1.5%)	47 (1.1%)	81 (1.2%)
Any other	15 (0.7%)	33 (0.8%)	48 (0.7%)
IMD quintile			
1 (Least deprived)	501 (22.8%)	850 (19.6%)	1,351 (20.7%)
2	512 (23.3%)	931 (21.5%)	1,443 (22.1%)
3	433 (19.7%)	825 (19.0%)	1,258 (19.3%)
4	393 (17.9%)	867 (20.0%)	1,260 (19.3%)
5 (Most deprived)	359 (16.3%)	860 (19.8%)	1,219 (18.7%)
Self-rated health			
Very good	971 (44.2%)	1,238 (28.6%)	2,209 (33.8%)
Good	856 (39.0%)	1,880 (43.4%)	2,736 (41.9%)
Fair	259 (11.8%)	876 (20.2%)	1,135 (17.4%)
Bad	82 (3.7%)	253 (5.8%)	335 (5.1%)
Very bad	29 (1.3%)	86 (2.0%)	115 (1.8%)

For peer review only

Appendix figures

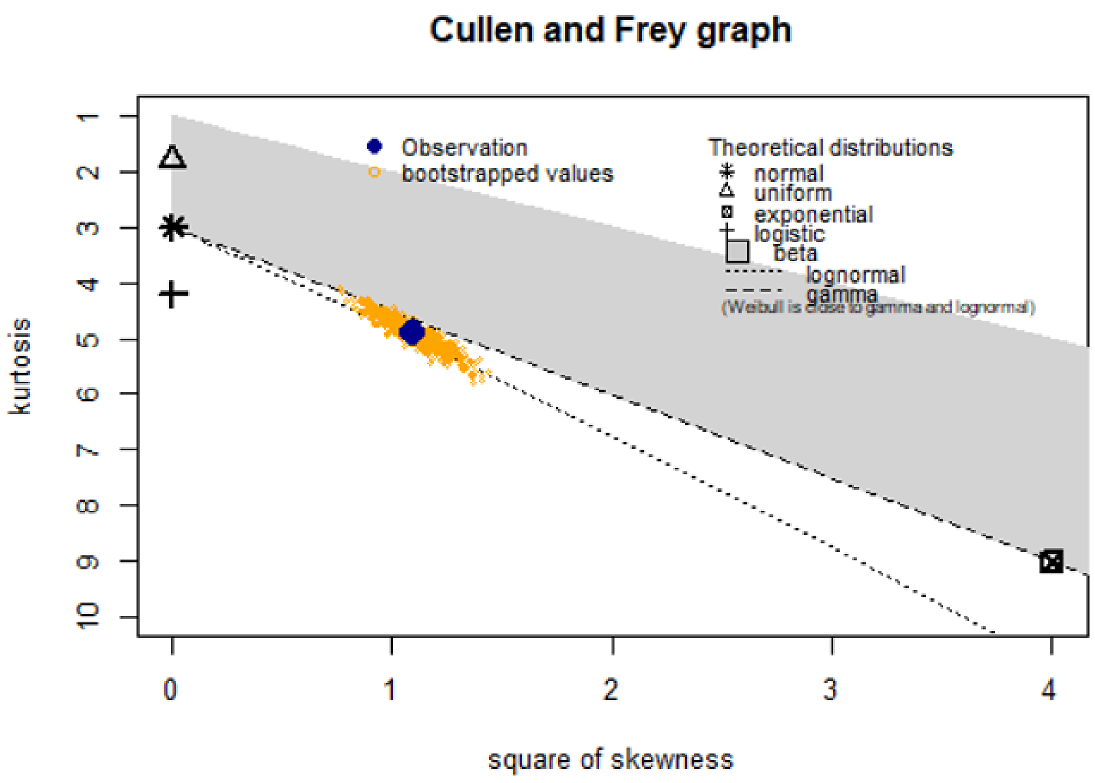

Appendix figure 1: Cullen-Frey plot exploring the mean-variance relationship of the model dependent BMI variable from HSE 2018, showing a marginally closer conformity with a lognormal than gamma distribution.

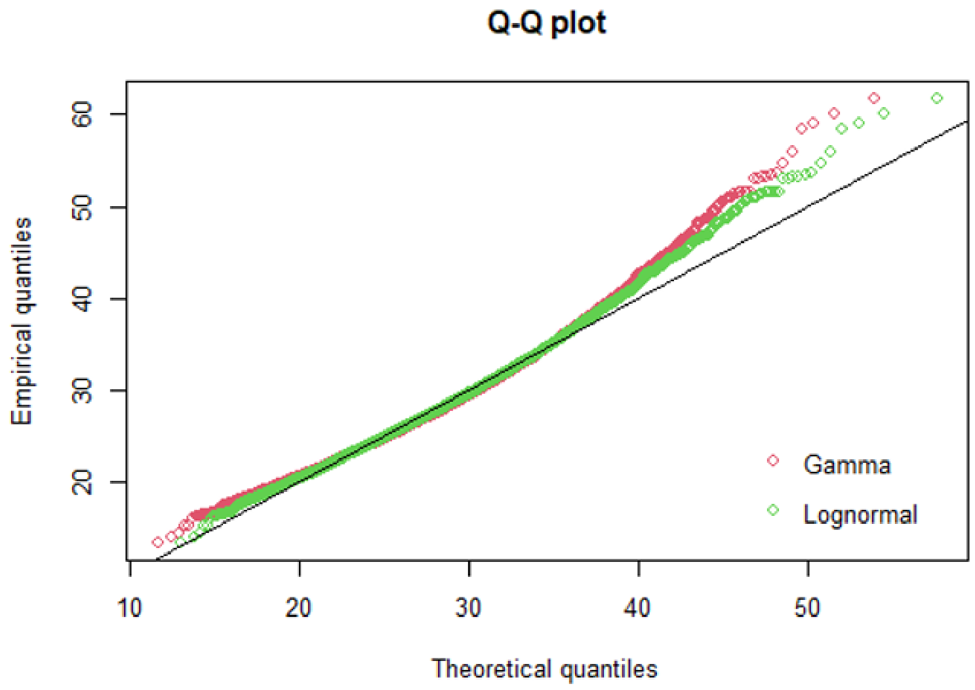

Appendix figure 2: Q-Q plot exploring the mean-variance relationship of the model dependent BMI variable from HSE 2018, detailing the deviation from gamma and lognormal distributions, showing a marginally closer conformity with a lognormal than gamma distribution.

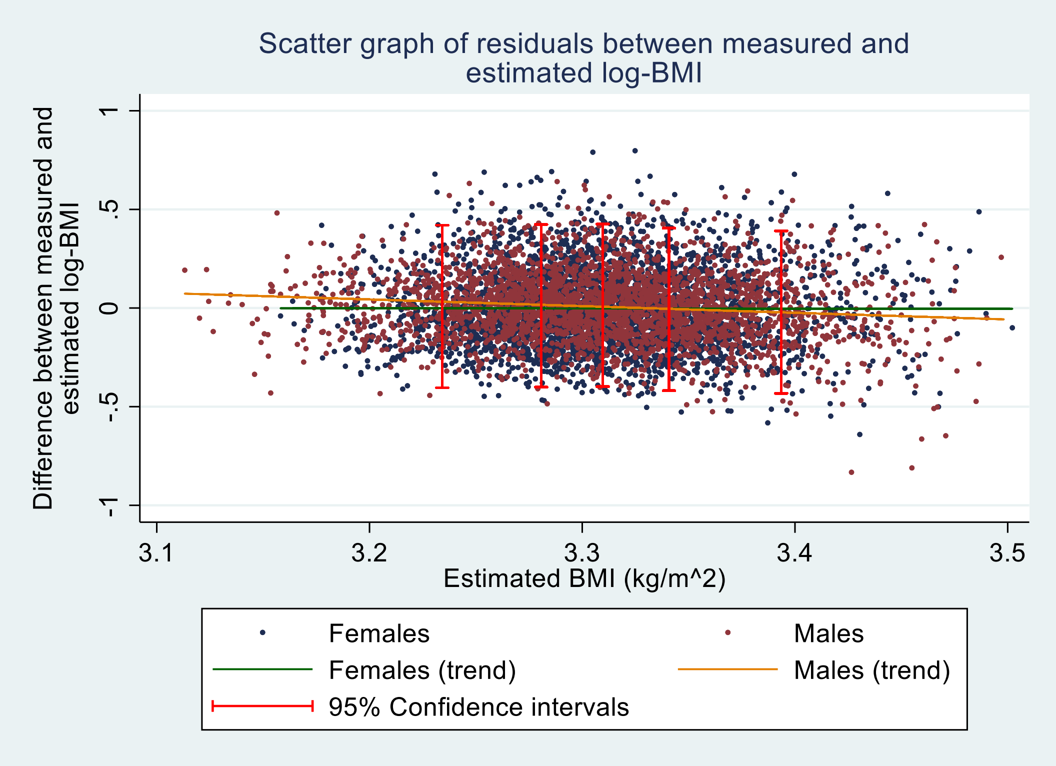

Appendix figure 3: Plot of residuals between measured log-BMI in the HSE 2018 and model-based estimates.

For peer review only

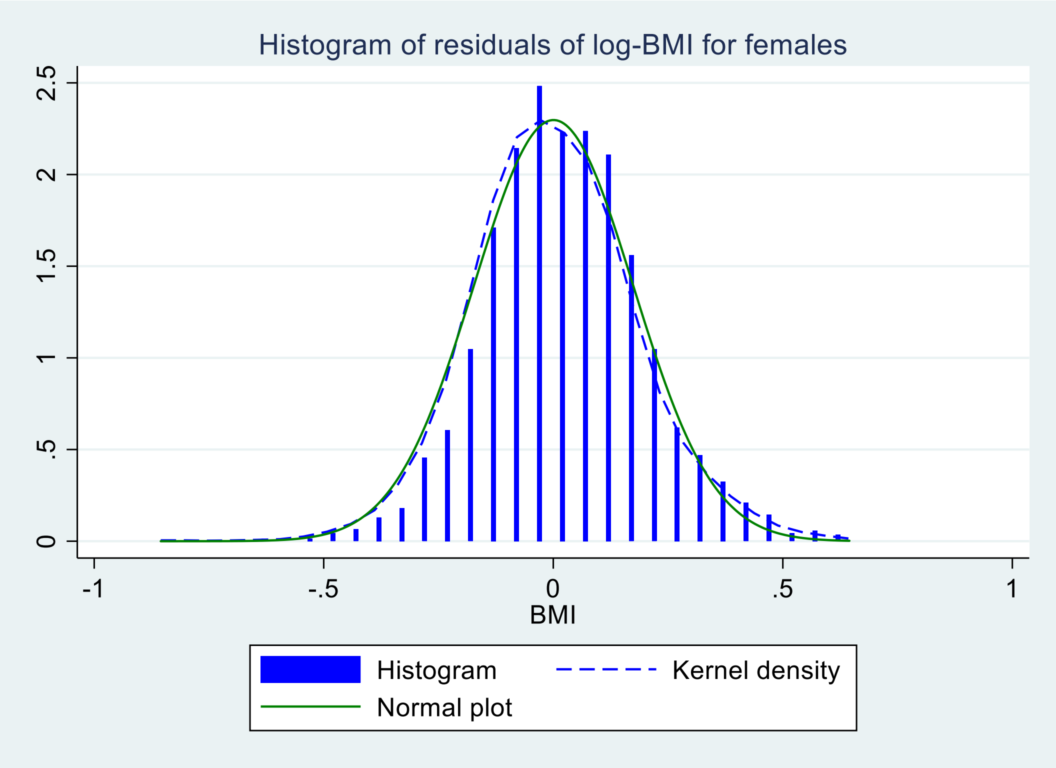

Appendix figure 4a: Histograms of residuals of between measured and estimated log-BMI for females to with kernel density and normal plot to examine for deviation from normality.

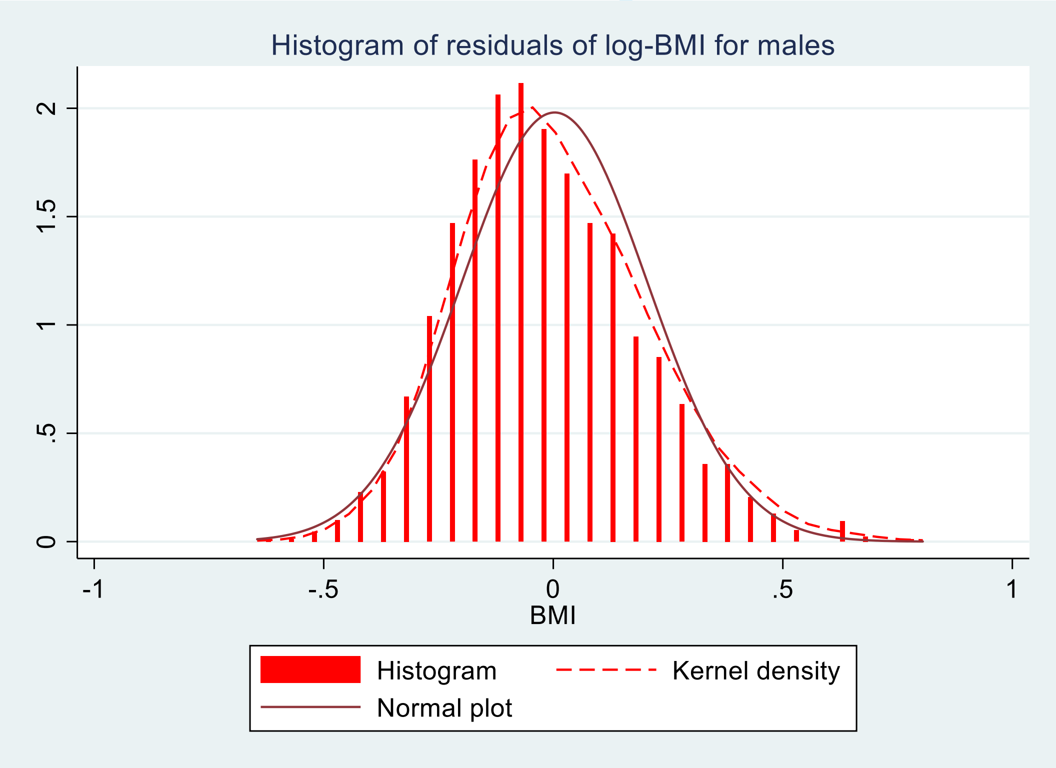

Appendix figure 4b: Histograms of residuals of between measured and estimated log-BMI for males to with kernel density and normal plot to examine for deviation from normality.

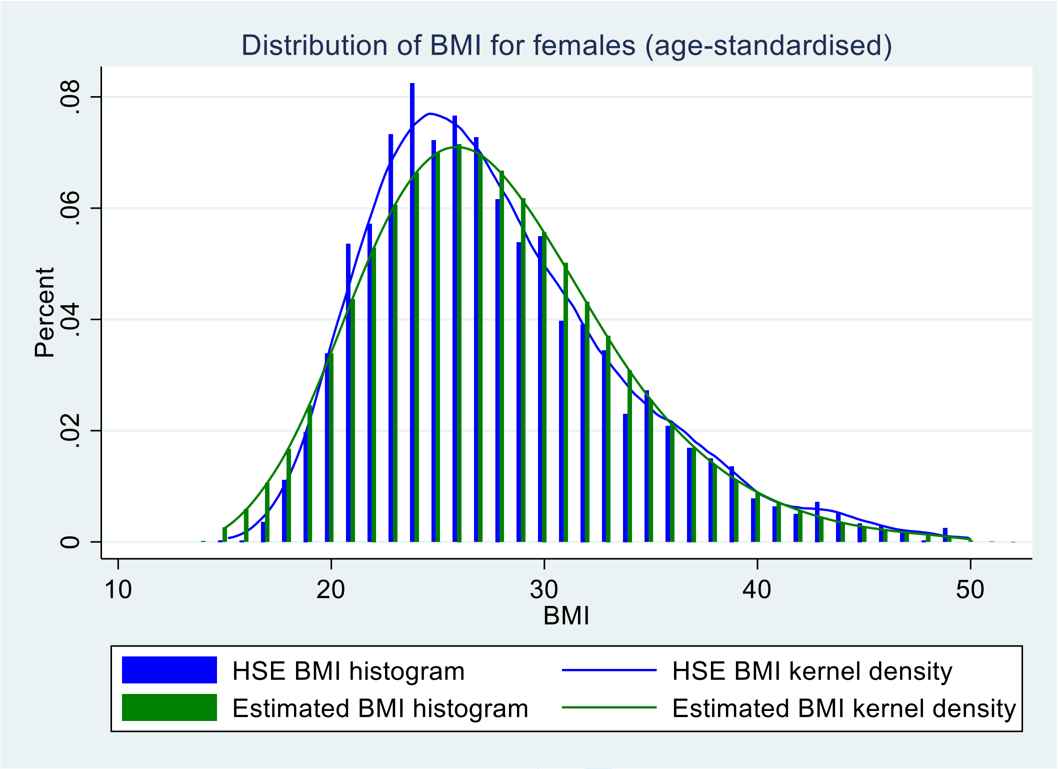

Appendix figure 5a: Histogram of age- and sex-standardised BMI estimates for England for females, including the equivalent histogram for the HSE 2018 BMI data for comparison.

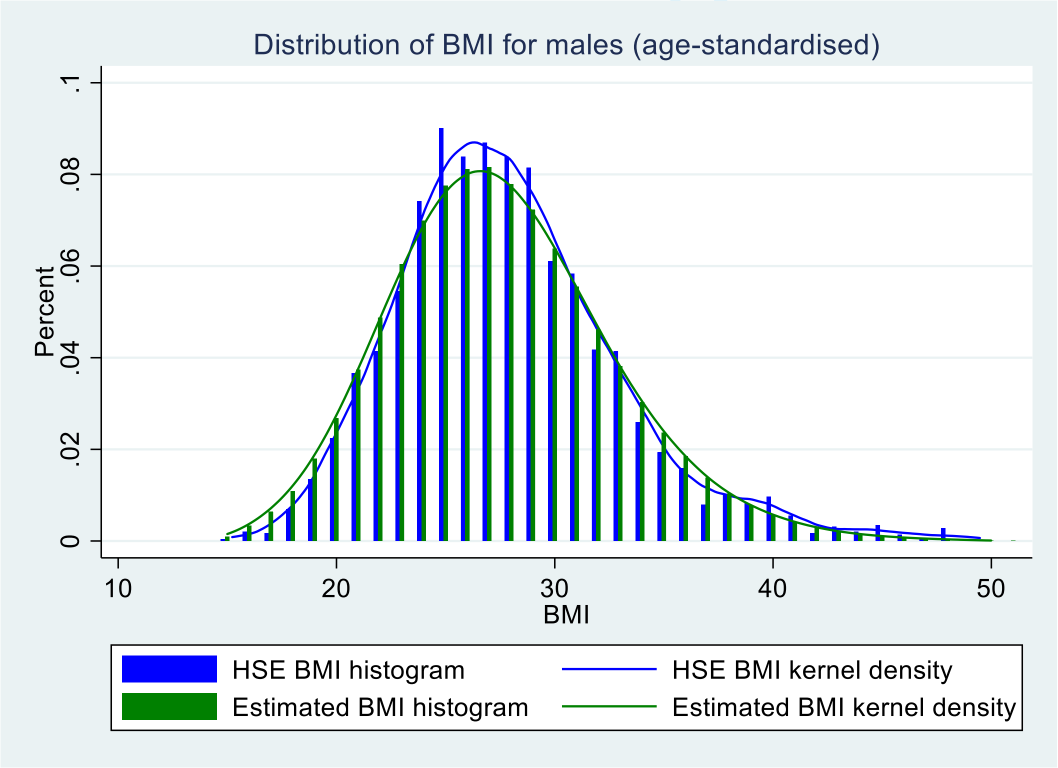

Appendix figure 5b: Histograms of age- and sex-standardised BMI estimates for England for males, including the equivalent histogram for the HSE 2018 BMI data for comparison.

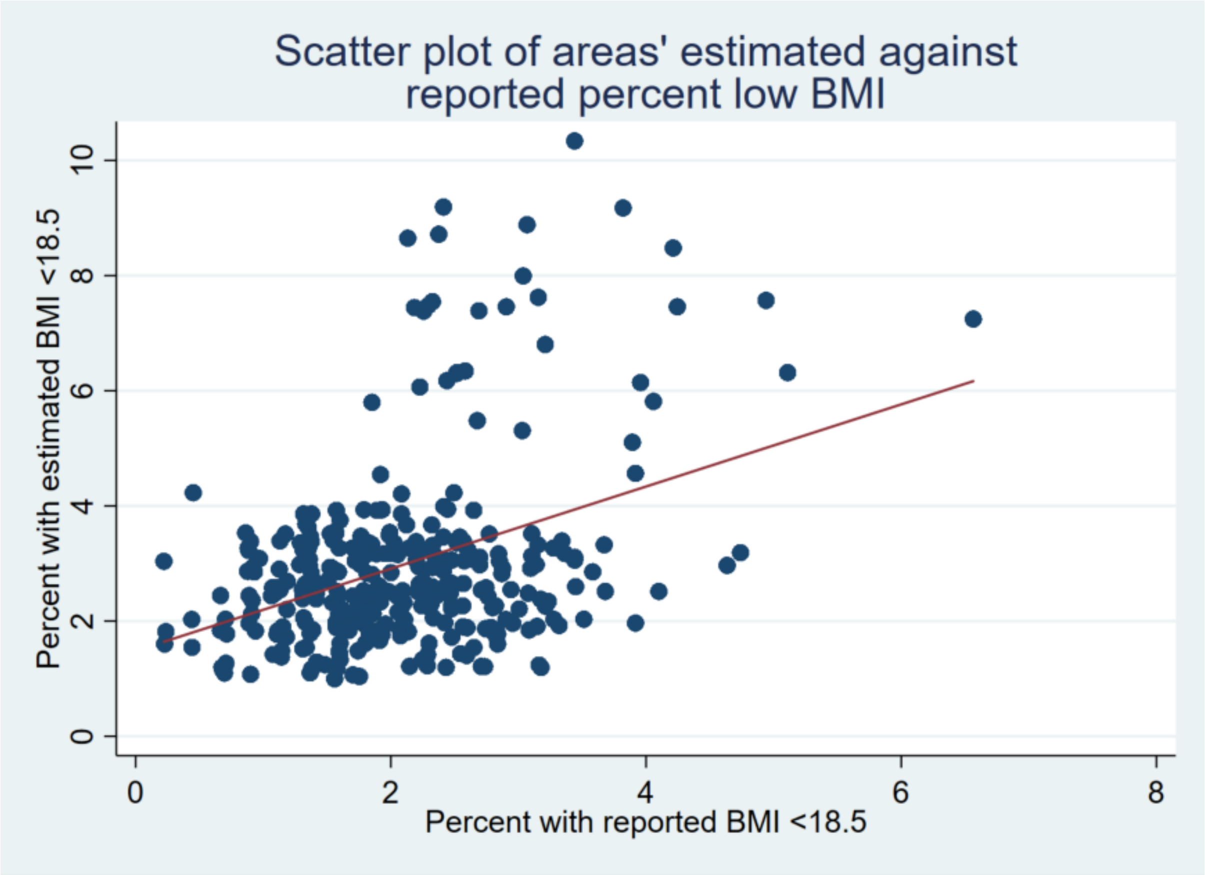

Appendix figure 6a: Scatter plot of the relationship between local areas' estimated versus reported percent of people with BMI <18.5.

For peer review only - http://bmjopen.bmj.com/site/about/guidelines.xhtml

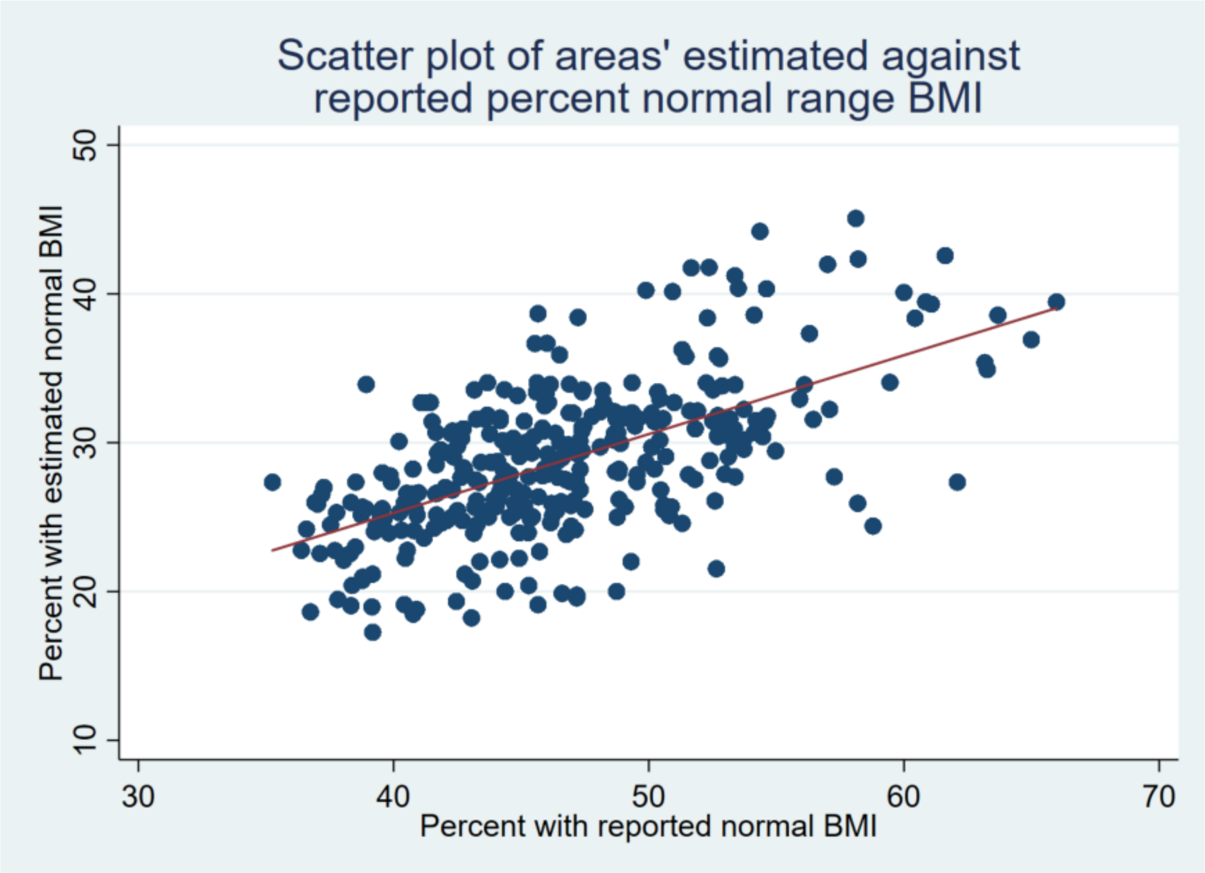

Appendix figure 6b: Scatter plot of the relationship between local areas' estimated versus reported percent of people with BMI in the normal range.

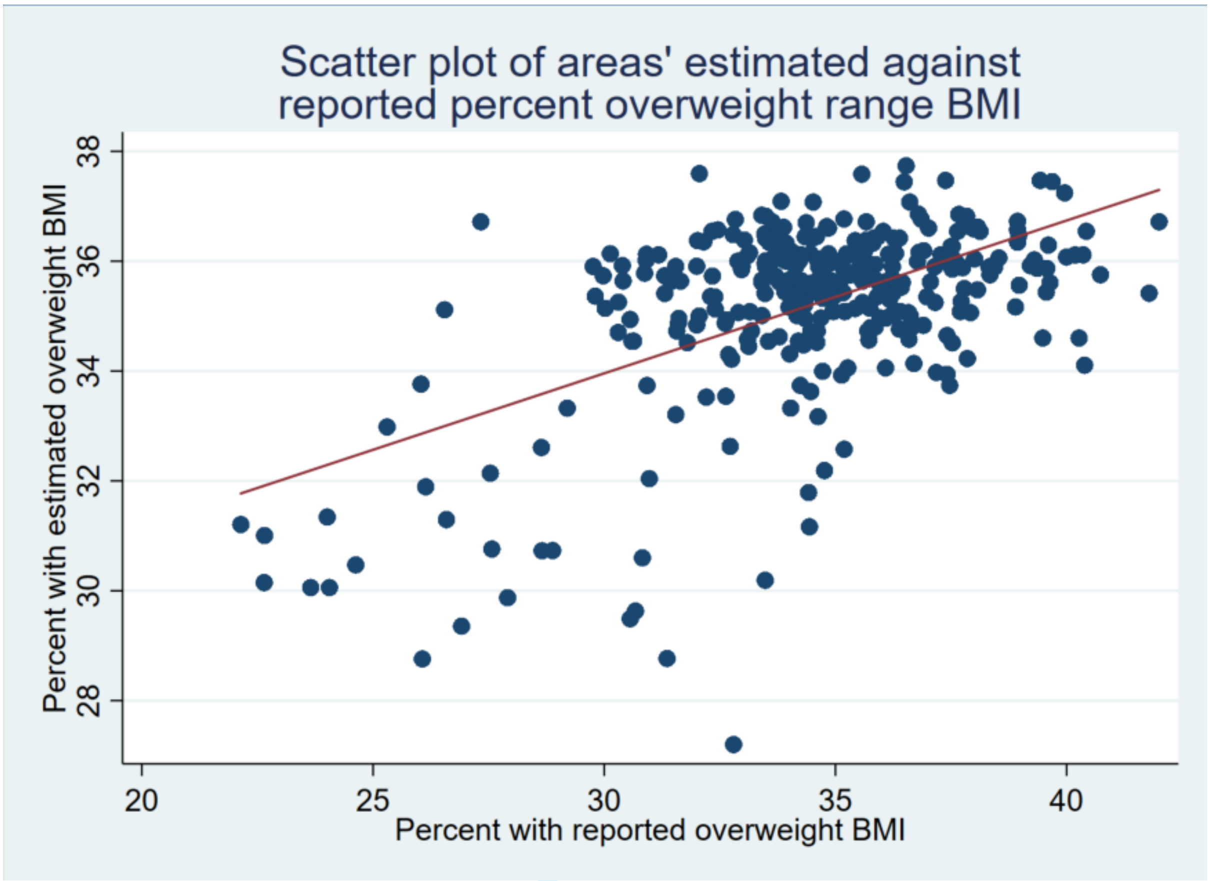

Appendix figure 6c: Scatter plot of the relationship between local areas' estimated versus reported percent of people with BMI in the overweight range.

For peer review only

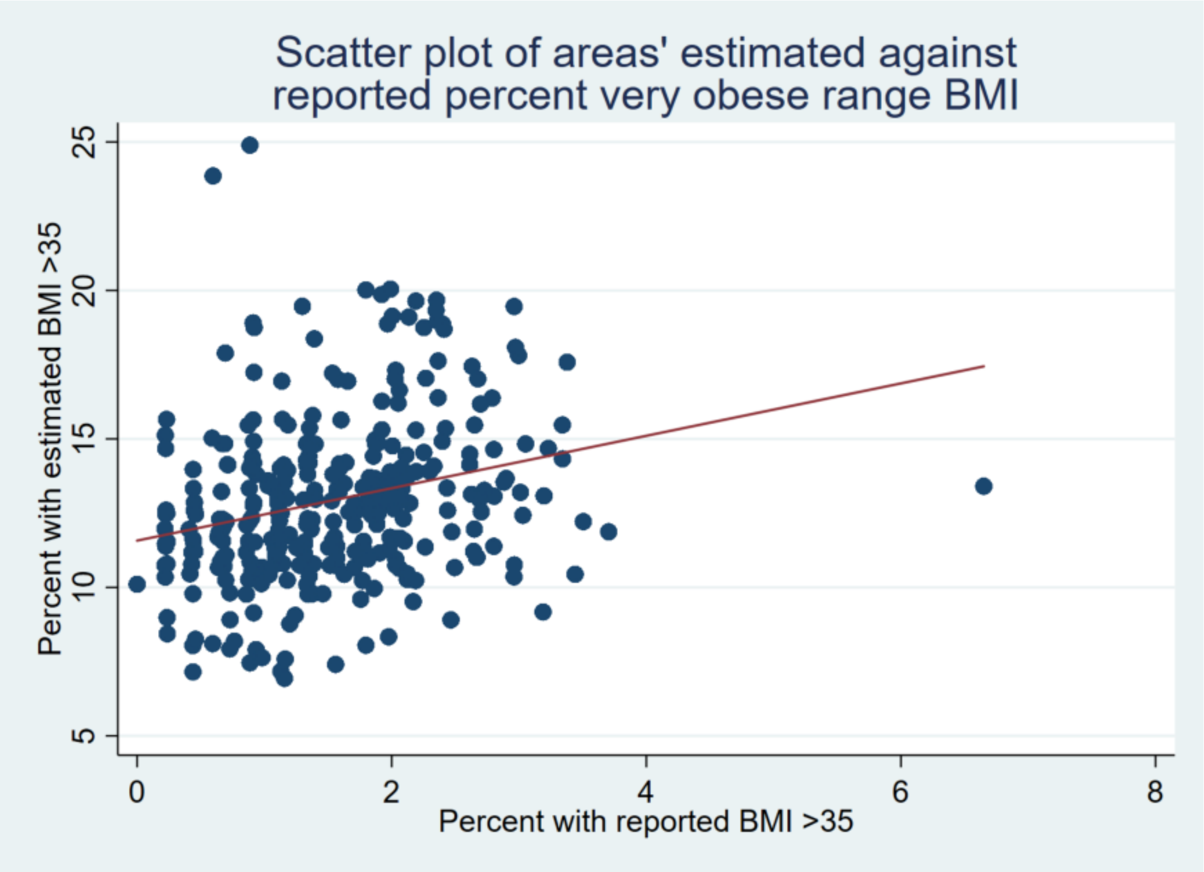

Appendix figure 6d: Scatter plot of the relationship between local areas' estimated versus reported percent of people with BMI >35.

For peer review only

BMJ Open

Estimating BMI distributions by age and sex for local authorities in England: a small area estimation study

Journal:	*BMJ Open*
Manuscript ID	bmjopen-2022-060892.R1
Article Type:	Original research
Date Submitted by the Author:	14-Apr-2022
Complete List of Authors:	Amies-Cull, Ben; University of Oxford, Nuffield Department of Population Health<br>Wolstenholme, Jane; University of Oxford, HERC, Nuffield Department of Population Health<br>Cobiac, Linda; Griffith University<br>Scarborough, Peter; University of Oxford, Nuffield Department of Population Health
**Primary Subject Heading**:	Public health
Secondary Subject Heading:	Nutrition and metabolism, Epidemiology
Keywords:	EPIDEMIOLOGY, PUBLIC HEALTH, STATISTICS & RESEARCH METHODS

SCHOLARONE™
Manuscripts

I, the Submitting Author has the right to grant and does grant on behalf of all authors of the Work (as defined in the below author licence), an exclusive licence and/or a non-exclusive licence for contributions from authors who are: i) UK Crown employees; ii) where BMJ has agreed a CC-BY licence shall apply, and/or iii) in accordance with the terms applicable for US Federal Government officers or employees acting as part of their official duties; on a worldwide, perpetual, irrevocable, royalty-free basis to BMJ Publishing Group Ltd ("BMJ") its licensees and where the relevant Journal is co-owned by BMJ to the co-owners of the Journal, to publish the Work in this journal and any other BMJ products and to exploit all rights, as set out in our licence.

The Submitting Author accepts and understands that any supply made under these terms is made by BMJ to the Submitting Author unless you are acting as an employee on behalf of your employer or a postgraduate student of an affiliated institution which is paying any applicable article publishing charge ("APC") for Open Access articles. Where the Submitting Author wishes to make the Work available on an Open Access basis (and intends to pay the relevant APC), the terms of reuse of such Open Access shall be governed by a Creative Commons licence – details of these licences and which Creative Commons licence will apply to this Work are set out in our licence referred to above.

Other than as permitted in any relevant BMJ Author's Self Archiving Policies, I confirm this Work has not been accepted for publication elsewhere, is not being considered for publication elsewhere and does not duplicate material already published. I confirm all authors consent to publication of this Work and authorise the granting of this licence.

For peer review only

Title:

Estimating BMI distributions by age and sex for local authorities in England: a small area estimation study

Corresponding author:

Ben Amies-Cull

Big Data Institute

Nuffield Department of Population Health

University of Oxford

Oxford, UK

OX3 7LF

ben.amies-cull@ndph.ox.ac.uk

Tel (+44 1865) 617784

Fax (NA)

Jane Wolstenholme, Nuffield Department of Population Health, University of Oxford, Oxford, UK

Linda Cobiac, Griffith University, Gold Coast, Australia

Peter Scarborough, Nuffield Department of Population Health, University of Oxford, Oxford, UK

Keywords:

Public health, epidemiology, obesity,

Word count: 3336

For peer review only

Abstract

Objectives

Rates of overweight and obesity vary across England, but local rates have not been estimated for over 10 years. We aimed to produce new small area estimates of BMI by age and sex for each lower-tier and unitary local authority in England, to provide up-to-date and more detailed estimates for the use of policymakers and academics working in non-communicable disease risk and health inequalities.

Design

We used Generalised Linear Modelling to estimate the relationship between BMI with social/ demographic markers in a cross-sectional survey, then used this model to impute a BMI for each adult in locally-representative populations. These groups were then disaggregated by 5-year age group, sex and local authority group.

Setting

The Health Survey for England 2018 (cross-sectional BMI data for England) and Census microdata 2011 (locally representative).

Participants

A total of 6,174 complete cases aged 16 and over were included.

Outcome measures

Modelled group-level BMI as mean and standard deviation of log-BMI. Extensive internal validation was performed, against the original data and external validation against the National Diet and Nutrition Survey and Active Lives Survey and previous small area estimates.

Results

In 94% of age-sex-are groups mean BMI was in the overweight or obese ranges. Older and more deprived areas had the highest overweight and obesity rates, which were particularly in coastal areas, the West Midlands, Yorkshire and the Humber. Validation showed close concordance with previous estimates by local area and demographic groups.

Conclusion

This work updated previous estimates of the distribution of BMI in England and contributes considerable additional detail to our understanding of the local epidemiology of overweight and obesity. Raised BMI now affects the vast majority of demographic groups by age, sex and area in England, regardless of geography or deprivation.

Strengths and limitations of this study

- Social and demographic heterogeneity is incorporated into analysis by using the Census microdata 2011 and GLM regression, allowing variation in exposure-effect relationships
- Extensive internal and external validation was performed in line with previous small area estimation studies

For peer review only

- Results required calibration, particularly affecting younger individuals in the microdata, to allow the distribution of BMI to closely reflect the base data
- It was not possible to estimate parameter uncertainty for these estimates
- Accounting for ethnicity-specific variation in BMI-risk relationships was not possible, for example for the variation observed between ethnic groups in the risk of type-2 diabetes at a given BMI.

Estimating BMI distributions by age and sex for local authorities in England

For peer review only

Introduction

Good quality primary data on Body Mass Index (BMI) do not exist for local areas of England. The sample size and sampling structure of national health surveys generally prevent health variables from being estimated at the local level by simple disaggregation, so a wide variety of local area estimation techniques have been developed.(1–3) These are largely designed to predict the prevalence of a categorical variable rather than a continuous distribution that is more appropriate for many variables such as BMI. These estimates are useful for policymakers at local and national levels to understand where and for who the burden of raised BMI is highest and where to direct scarce resources. They also provide a valuable input to further research into BMI-related risk and related health inequalities.

The most recent set of estimates for BMI at the local authority level in England are by Moon *et al* (2007)(4) based on data from 1998-99. Their paper used multilevel modelling to estimate the proportions of each local authority in the overweight and obese BMI categories. This found obesity rates were highest in parts of the West Midlands, Yorkshire and the Humber, and overweight rates highest in coastal areas, particularly in Devon and Cornwall.

Survey data exist for estimating risk factor prevalence in England from studies such as the Health Survey for England (HSE),(5) National Diet and Nutrition Survey (NDNS)(6) and Active Lives Survey (ALS),(7) but each of these has limitations that prevent them from giving precise estimates of risk factor prevalence for age and sex groups at the local authority level. Both the HSE and NDNS are underpowered for such estimates and they do not report each respondent's local authority. The ALS collects self-reported height and weight data from phone interview and reports data at the local authority level, but only reports BMI as BMI category (eg. overweight, obese). These surveys suffer with the common problems for small area estimation(8) that they do not specify the local geographic area of the respondent and their sample sizes are too small to deliver precise estimates of risk factors for subgroups. To overcome these problems with survey data, a wide variety of small area estimation methods have been developed to allow researchers to estimate the prevalence of risk factors for small geographical areas. These methods can be grouped into three broad approaches:(1–3) indirect standardisation, model-based estimation and microsimulation approaches. The indirect standardisation approach involves calculating the proportion of the population with a feature of interest (eg. BMI in the overweight range) for different subgroups, then weights these proportions according to the subgroups' representation in each local area. Model-based estimation improves on this approach by using logistic regression (usually) to estimate the

proportion of a feature in a local area based on individual-level and area-level variables, using aggregate data on the frequency of these variables.(1,2) This approach was improved on by using Census data to increase the number of variables available for use.(9) To better account for unmeasured variation between areas, for example related to more granular sociodemographics, local context and culture,(10) Twigg *et al*(11) developed an approach using multilevel logistic regression (later used for the BMI estimates by Moon *et al* 2007,(4) mentioned above). Finally, microsimulation approaches combine spatially aggregated survey data with geographically disaggregated microdata (that may need to be simulated)(8) but estimating credible intervals is challenging.(12,13)

We set out to estimate and validate the continuous distribution of BMI by age and sex in each local authority in England. We built on previous approaches to estimate continuous distributions, combining the strengths of model-based estimates and microsimulation.

Methods

Producing the modelled estimates of BMI distributions involved the following steps. First, we selected appropriate national-level survey data that included a BMI variable. Then we identified social and demographic variables that existed in both the health survey and the Census microdata 2011 (comprising every 20th entry from the UK Census 2011, available for all local authorities in England).(14) We then fit a model relating these variables with BMI on the health survey data and applied the model to each respondent in the Census microdata, to generate predictions of BMI at the individual level. These estimates were validated using established approaches.(12,15)

Data

For the national-level survey data on BMI, the most recent wave of HSE (2018)(5) was chosen as the BMI dataset on the basis that the alternative of the NDNS 2014-16 has a much smaller sample size than the HSE(6) and the ALS 2017 has only a self-reported categorical BMI variable.(7)

The local authority-level data were taken from the Census microdata 2011, which is a 5% sample of anonymised individual-level Census records, with the lowest geographic level at the lower tier or unitary local authority (UK local government, mostly 150,000-500,000 persons, of which there are 315 in England, plus the Isles of Scilly and City of London), or small groups of these termed “local authority Census groups” where populations are small.

‘Candidate variables’ for the model were social or demographic markers that were available in the same or translatable forms in the HSE 2018 and the Census microdata. These were age, sex, ethnicity, self-rated health (from 1 (good) to 5 (poor)), number of cars in the household, socioeconomic status (SES, using the National Statistics Socioeconomic Classification), NUTS-1 region (the nine regions of England), tenure status (ie. living arrangements, such as renters), and dummy variables for being unemployed, being a student, having no formal qualifications, and having a degree. Index of Multiple Deprivation (IMD) is given in the HSE at the postcode level, but there is no IMD variable given in the Census microdata, so it had to be merged at the local authority level from an external dataset.(16) Appendix table 1 provides a full description of these candidate variables and adaptations to make them equivalent. Participants in the HSE with missing data either for BMI or any of the candidate variables were dropped, detailed in Appendix tables 2 and 3.

Generalised Linear Models

For peer review only

The estimation process involved building a Generalised Linear Model (GLM) regression model in HSE 2018 with BMI as the dependent variable and the candidate variables as independent variables. This model was then used to predict a BMI for each individual in the UK Census 2011 microdata. To specify the GLM model, the data were first examined. The variance function (or family) was chosen on the basis of examination of the BMI variable, which identified a continuous skewed bell-shaped distribution, consistent with either a gamma or a gaussian (including log-normal) mean-variance relationship. Formal tests for family choice such as the modified Park test(17) could not be used as they rely on squared error and are not valid with clustered sampling. The Cullen-Frey plot and QQ-plot in Appendix figures 1 and 2, respectively, indicate the BMI variable conformed more closely with a lognormal than gamma distribution. Therefore, the GLM was built specifying a gaussian family with log link, as has become an accepted practice(17,18) for log-normally distributed dependent variables. A frequency weight for the nurse visit was applied that was developed by the HSE team to account for non-response to the nurse visit, allowing representativeness to the general population. Cluster robust standard errors were specified to account for clustered sampling in HSE.(19)

The model was built by regressing each variable against BMI for individuals aged 16 and over and including it in the model if Bayesian Inference Criterion (BIC) indicated an improved model (with an arbitrary cutoff of -2 to indicate superiority). BIC was used over the Akaike Information Criterion as BIC prefers more parsimonious models. Likelihood Ratio tests were not used as they are invalid with robust standard errors.(16) Ordinal variables were assessed as continuous before assessing if the levels contributed beyond linearity. If the final model output Wald Test did not support a significant association (at p<= 0.05), the variable's contribution was re-assessed by BIC.

Outputs were used to impute BMI in the Census microdata for each respondent aged 16 and over. GLM log-link variables are given in the log scale and interpreted multiplicatively as:

$$E(Y) = \prod_{i=0}^{n} \exp\left(\beta_i X_i\right)$$

Where E(Y) is the estimate of the independent variable (BMI), X_i are variable values (where $X_0 = 1$ for the constant) and β_i are coefficients. The process was as follows:

- Each individual's variable values were multiplied by their coefficients, then these were exponentiated, then multiplied together to give their final imputed BMI value.
- Each individual's coefficient values had random variation added to represent subgroup heterogeneity. This was done by adding random variation to these values on a normal distribution with standard deviation equal to the standard error of the coefficient from the GLM.

Calibration of the results was required to broaden the spread of data points to meet that in the natural population.

- For this, the SD of log-BMI was calculated for each age-sex group in the HSE data and these values were smoothed out by using a linear regression model to generate predicted values, using age and sex as independent variables.
- Uncalibrated individual estimates of log-BMI were disaggregated by age group and sex, then the mean of that group subtracted, results divided through by that group's SD to give a z-score.
- This was then multiplied up by the smoothed modelled SD from HSE and the group mean added back on, before exponentiating log-BMI back to BMI.

For peer review only

Validation steps

Three validation steps were used, based on previous approaches.(12,15)

1. Estimates were assessed for heteroskedasticity by examining residual plots of log-BMI. Formal tests for heteroskedasticity (eg. Breush-Pagan test) are not valid with robust standard errors.
2. Internal validity was tested by comparing age-standardised distributions of estimated and measured BMI (from HSE 2018) by sex.
3. External validity was tested in three ways.
   - Comparing age-standardised distributions of estimated and measured BMI from NDNS 2014-16 by sex
   - For each local authority, the estimated obesity rate was plotted against the obesity rate according to self-reported BMI from the ALS 2017.
   - Results were compared with Moon *et al* 2007,(4) in terms of the areas in the top decile for obesity rates and for overweight rates.

Public and patient involvement statement

Patients or the public were not involved in the design, or conduct, or reporting, or dissemination plans of our research.

Ethics

The terms of the Central University Research Ethics Committees were reviewed. The study did not require ethics approval as only pre-collected data on non-identifiable individuals was used.

Results

BMI was estimated for adult males and females across 16 age groups in 249 local authority census areas of England, making 7,968 subgroups. In 94% of these subgroups the mean BMI was in the overweight range (97% for males, 90% for females). In no area was mean BMI in the normal range between the ages of 50 and 85 years for either sex. Mean BMI, mean log-BMI and SD of log-BMI by age group, sex and local area are available from Oxford Research Archive. Appendix table 4 shows that people with raised BMI in HSE 2018 tended to be older and more deprived and had worse health. There were 6174 complete cases in HSE and Appendix table 2 shows details of missing data, identifying 18% missing for the BMI variable and Appendix table 3 shows that older people are overrepresented in these missing data.

For peer review only

Model results:

Table 1 shows the coefficients for each of the variables fitted in the final model, with their 95% confidence intervals.

Table 1: Model variables, coefficients and coefficient 95% confidence intervals given to 3 significant figures (* = interaction between stated variables). IMD = Index of Multiple Deprivation, SES = socioeconomic status.

Variable	Coefficient (log scale)	95% confidence intervals Lower	Upper
Age (years)	0.0114	0.00932	0.0136
Age (years) squared	-9.81×10^{-05}	-0.000119	-7.70×10^{-5}
Female (dummy)	0.108	0.0288	0.188
Female*age	-0.00398	-0.00709	-0.000874
Female*age squared	3.22×10^{-5}	3.66×10^{-6}	6.08×10^{-5}
Ethnicity			
White/ mixed (base)			
Black	0.0483	0.0147	0.0819
Asian	-0.0219	-0.0435	-0.000323
Other	-0.0423	-0.0901	0.00538
Self-employed (dummy)	-0.0159	-0.0337	0.00200
Retired (dummy)	0.00450	-0.0154	0.0244
Long-term sick (dummy)	-0.0221	-0.0653	0.0210
No qualifications (dummy)	-0.00621	-0.0209	0.00844
Degree holder (dummy)	-0.0286	-0.0407	-0.0164
IMD			
Quintile 1 (base, lowest deprivation)			
Quintile 2	0.00732	-0.00890	0.0235
Quintile 3	0.0130	-0.00446	0.0305
Quintile 4	0.0176	0.000301	0.0349
Quintile 5	0.0315	0.0116	0.0514
Health status			
Very good (base)			
Good	0.0574	0.0467	0.0682
Fair	0.108	0.09207	0.124
Bad	0.109	0.0794	0.138
Very bad	0.142	0.0838	0.200
Tenure status			
Own outright (base)			
On a mortgage	-0.00130	-0.0178	0.0152
Shared ownership	0.0275	-0.0525	0.107
Rental	0.0192	0.00304	0.0354
Living rent-free	0.0684	-0.000499	0.137
Region			
North East (base)			
North West	-0.00122	-0.0249	0.0225
Yorks and Humber	0.0209	-0.00582	0.0477

For peer review only

East Midlands	0.00591	-0.0194	0.0312
West Midlands	0.0202	-0.00633	0.0467
East of England	0.0122	-0.0140	0.0384
London	-0.0289	-0.0540	-0.00373
South East	0.0162	-0.00975	0.0423
South West	-0.00181	-0.0259	0.0222
SES			
AB – managerial and professional (base)			
C1 – intermediate occupations	0.00200	-0.0177	0.0217
C2 – Small employers and own account workers	0.0141	-0.0113	0.0395
DE – lower supervisory, technical, and semi-routine occupations	-0.0116	-0.0280	0.00480
Other/ no response	-0.0123	-0.0366	0.0120
Constant	2.97	2.91	3.03

After standardising for age, mean BMI was 27.7kg/m^2 for females and 27.9kg/m^2 for males, though the distribution was broader for females, with higher proportions in low and high categories. Figure 1 shows the orders of magnitude of difference between local authorities across different quintiles of deprivation with the England average for reference. Even for the lowest deprivation area, almost all age-sex groups have mean BMI in the overweight range.

Validation

Heteroskedasticity

Residual plots (Appendix figure 3) demonstrated homoskedasticity. Trend lines show no overall bias across measured log-BMI and residuals' are shown to be normally distributed in Appendix figures 4a and 4b.

Internal validity

The age-standardised England-level distributions of estimated BMI for females and males are compared with the equivalent distributions of measured BMI from the original HSE 2018 data, shown in Appendix figures 5a and 5b, showing close concordance with the base data.

Figure 2 compares the 25th, 50th and 75th centiles of the calibrated and uncalibrated estimates with the HSE data across the age range across England, standardising the populations to represent females and males equally. These show that calibration mainly affected younger age groups and produced distributions closely in alignment with those in the HSE.

External validity

Estimated BMI distributions for females and males were compared with the equivalent distributions from the independently-collected NDNS 2014-16 data in figures 3a and 3b (standardised to

For peer review only

represent each age group equally). Figure 4 plots the reported proportion of people with BMI in the obese range for each local authority in the ALS against the same proportions on the new estimates. This shows a close relationship, though the proportion in the obese range is approximately 5 percentage points higher in the estimates than ALS across the distribution. Appendix figures 6a-6d show graphs for the other BMI categories.

Comparison of model results with those by Moon *et al* (2007)(4) shown in figure 5. These maps would not be expected to be the same as the data they are estimated on are 20 years apart. However, where specific areas are not the same, adjacent and similar types of areas are highlighted, namely mainly Yorkshire and the Humber, the West Midlands, coastal and isolated rural areas. Coefficients used in their model were similar to this paper: individual-level age, sex, Black ethnicity, Asian ethnicity, and area-level percentages of low social grade, high social grade, households with dependent children, in local authority housing, and adults married. Likewise, their greatest coefficients were with age and sex, but Black and Asian ethnicities were more important in their paper while health status more.

For peer review only

Discussion

This paper introduces a method that combined the individual-level associations of BMI with social and demographic features to estimate the BMI of each individual in the Census microdata 2011, allowing a mean BMI and associated distribution to be estimated for each five-year age and sex group for each local authority. It builds on the established methods estimating local BMI(1,4,9,20) while adding further detail by estimating continuous distributions of BMI. These will provide policymakers and academics much greater detail on BMI-related risk, allowing policy impact on health inequalities to be better understood and enabling resources to be better targeted.

The distributions of age- and sex-level estimates were calibrated to replicate the national distribution of BMI. Figure 2 shows that this process produced highly consistent distributions with the underlying data and calibration affected younger ages groups more. Females had higher proportions with low and high BMI. BMIs were higher in older and more deprived areas.

Examination of residuals identified homoskedasticity (Appendix Figure 2), in the context of the GLM implying that the link function was appropriately specified and that the model was well-fitted. Internal validation against HSE showed good concordance. External validation at the local authority level against the ALS in Figure 4 showed a close linear relationship between the proportion of the population in the obese range, though with a distribution approximately 5 percentage points higher in the estimates. This could be due to the reporting bias of the self-reported height and weight items in the ALS or response bias of people with lower BMI into the survey. The other internal and external validation exercises indicate that this is unlikely to be a consequence of bias in the estimates.

Comparison of the new estimates against those from Moon *et al* 2007(4) show close alignment. Many of the same local areas are identified, such as Birmingham, Humberside, Lincolnshire and the south coast. In others, if the same area was not identified then neighbouring areas usually were, such as around Norwich.

Strengths and limitations

This novel approach of estimating BMI at the individual level has allowed estimates to be drawn with a greater degree of detail than previously, namely estimating the whole BMI distribution and drawing estimates for many subgroups. The HSE and Census microdata are both highly granular and representative of the population and data quality of the HSE is generally good, being a large and representative sample. One relevant limitation to the HSE 2018 is missing data, namely for the BMI variable, especially from the late 70s (Appendix table 3). One potential reason that older people are underrepresented may be poor health. While it is not possible to account for systematic differences in non-responders, health is captured in the model, while the Census should provide a representative estimate of health in each age group, helping to avoid bias from these missing data.

The range of variables that can be aligned between datasets is also a limitation, in common with previous local area estimation methods.(11) First, the levels of geography do not align apart from at the NUTS-1 level. This means that multilevel modelling previously used(4,11,21,22) is not feasible, as the NUTS-1 areas are far too large and internally heterogeneous to pick up local authority-sized variation. Therefore, this method assumes that the national-level associations between the predictors and BMI do not systematically vary between local areas. Second, deprivation is measured at the postcode level in the HSE 2018 but is merged at the local authority level into the Census data. This means that deprivation is not heterogeneous within local authorities in the Census as it is in the real world, so resulting in slightly narrower modelled BMI. A calibration step was used to allow estimated distributions to closely reflect the real world, relying on an assumption that the order of age-sex-area-level estimates produced by the model was correct.

The Census data is, of writing, ten years old. Ageing populations are not directly important as age is accounted for, but local changes (such as increasing education levels) may lead to bias in estimates. As the model was built using recent HSE and IMD data, change in association between variables and BMI over time should not be a source of bias. The final limitation is that estimating the parameter uncertainty to a variable's estimated mean in small area estimation methods is difficult (8,12) and not attempted here.

The use of BMI as a predictor of health outcomes has been critiqued on the basis of heterogeneity in the population, particularly around ethnic differences. Different BMI cutoffs for disease risk have been described, for example identifying that for the same risk of developing T2DM as white people with a BMI of 30kg/m^2, South Asians only needed a BMI of 23.9kg/m^2 and black people a BMI of 28.1 kg/m^2.(23) While it remains unclear why this pattern has arisen,(23) this correction could make a meaningful difference to disease risk in some areas with greater proportions of non-white people. Therefore, these results may need to be interpreted with such variation in mind and this topic remains an important focus for future research.

Conclusion

This study used HSE 2018 to estimate the BMI of respondents in the Census microdata 2011, providing the most recent set of disaggregated small area estimates for BMI at the local authority level in England since 2007.(4) Granular social and demographic information provided by the Census microdata 2011 captured local variation. Results showed that over 50% of people in almost every demographic group and area had above-normal BMI.

Validation showed there was a low risk of heteroskedasticity and there were approximately normally-distributed residuals, providing confidence that models were well-fitted. Comparison of estimates against external datasets at the national and regional levels identified close concordance

For peer review only

with previous findings, but comparison at the local authority level with self-reported BMI implied that many areas have higher rates of raised BMI than previously(24) estimated.

These new estimates go further than previous studies by two dimensions - by estimating BMI by age-sex groups and by modelling the distribution of BMI rather than percentages in BMI categories. As such, this study has produced the most demographically granular estimates for BMI in local areas of England to date.

Contributorship statement

BAC conceived of the study, contributed to study design, undertook the main analysis and completed the first draft of the manuscript.

JW conceived of the study, contributed to design, supervised the analysis and commented on drafts of the manuscript.

LC conceived of the study, contributed to design, supervised the analysis and commented on drafts of the manuscript.

PS conceived of the study, contributed to design, supervised the analysis and commented on drafts of the manuscript.

Competing interest statement

BAC is supported by the MRC, JW by the NIHR and CRUK, and PS by the BHF and NIHR Oxford Biomedical Research Centre.

Funders had no involvement in the study design; in the collection, analysis and interpretation of the data; in the writing of the report; or in the decision to submit the paper for publication.

Funding statement

This work was funded by the Medical Research Council grant number MR/N013468/1.

Data sharing statement

Results are available at Oxford Research Archive https://doi.org/10.5287/bodleian:JbamnGwNG, provided as mean BMI, mean and SD of log-BMI for each age and sex group for each local authority area.

For peer review only

For peer review only

References

1. Scholes S, Bajekal M. Synthetic estimation of healthy lifestyles indicators: User guide [Internet]. Available from: https://www.researchgate.net/publication/265658543

2. Marshall A, Economic and Social Data Service. Small area estimation using ESDS government surveys – An introductory guide. 2010.

3. Rahman A. Estimating small area health-related characteristics of populations: A methodological review. Geospatial Health. 2017;12(1).

4. Moon G, Quarendon G, Barnard S, Twigg L, Blyth B. Fat nation: Deciphering the distinctive geographies of obesity in England. Social Science and Medicine. 2007 Jul;65(1):20–31.

5. [Dataset] National Centre for Social Research (NatCen), University College London, Department of Epidemiology and Public Health. (2020) [data collection]. Health Survey for England, 2018. UK Data Service. SN: 8649, http://doi.org/10.5255/UKDA-SN-8649-1.

6. [Dataset] University of Cambridge, MRC Epidemiology Unit, NatCen Social Research. (2021). National Diet and Nutrition Survey Years 1-11, 2008-2019 [data collection].19th Edition. UK Data Service. SN: 6533, http://doi.org/10.5255/UKDA-SN-6533-19.

7. [Dataset] Sport England. (2020). Active Lives Survey, 2017-2018 [data collection]. UK Data Service. SN: 8651, http://doi.org/10.5255/UKDA-SN-8651-1.

8. Rahman A, Harding A. Small area estimation and microsimulation modeling. Boca Raton; 2019.

9. Charlton J. Use of the Census Samples of Anonymised Records (SARs) and survey data in combination to obtain estimates at local authority level. Vol. 30, Environment and Planning A. 1998.

10. Macintyre S, Ellaway A, Cummins S. Place effects on health: how can we conceptualise, operationalise and measure them? Vol. 55, Social Science & Medicine. 2002.

11. Twigg L, Moon G, Jones K. Predicting small-area health-related behaviour: a comparison of smoking and drinking indicators [Internet]. 2000. Available from: www.elsevier.com/locate/socscimed

12. Edwards KL, Clarke GP, Thomas J, Forman D. Internal and External Validation of Spatial Microsimulation Models: Small Area Estimates of Adult Obesity. Applied Spatial Analysis and Policy. 2011 Nov;4(4):281–300.

13. Whitworth A, Aitken G, Mclennan D, Taylor J. Evaluations and improvements in small area estimation methodologies, National Centre for Research Methods Methodological Review paper. 2013.

14. [Dataset] Office for National Statistics. (2014). 2011 Census Microdata Individual Safeguarded Sample (Regional): England and Wales [data collection]. UK Data Service. SN: 7605, DOI:10.5255/UKDA-SN-7605-1.

For peer review only

15. Scarborough P, Allender S, Rayner M, Goldacre M. Validation of model-based estimates (synthetic estimates) of the prevalence of risk factors for coronary heart disease for wards in England. Health and Place. 2009;15(2):596–605.

16. English Indices of Deprivation 2019, Department for Communities and Local Government, Crown Copyright 2019, https://www.gov.uk/government/statistics/english-indices-of-deprivation-2019.

17. Partha D, Norton. Edward, Manning W. Health Econometrics Using Stata. College Station, Texas, 2017.

18. Mohammad N, Mcgivern L. Comparing Gamma and Log-Normal GLMs in R Using Simulation and Real Data Set.

19. Mansournia MA, Nazemipour M, Naimi AI, Collins GS, Campbell MJ. Reflection on modern methods: Demystifying robust standard errors for epidemiologists. International Journal of Epidemiology. 2021 Feb 1;50(1):346–51.

20. Edwards KL, Clarke GP. The design and validation of a spatial microsimulation model of obesogenic environments for children in Leeds, UK: SimObesity. Social Science and Medicine. 2009 Oct;69(7):1127–34.

21. Moon G, Twigg L, Jones K, Aitken G, Taylor J. The utility of geodemographic indicators in small area estimates of limiting long-term illness. Social Science and Medicine. 2019 Apr 1;227:47–55.

22. Whitworth A, Carter E, Ballas D, Moon G. Estimating uncertainty in spatial microsimulation approaches to small area estimation: A new approach to solving an old problem. Computers, Environment and Urban Systems. 2017 May 1;63:50–7.

23. Caleyachetty R, Barber TM, Mohammed NI, Cappuccio FP, Hardy R, Mathur R, et al. Ethnicity-specific BMI cutoffs for obesity based on type 2 diabetes risk in England: a population-based cohort study. The Lancet Diabetes and Endocrinology. 2021 Jul 1;9(7):419–26.

24. Obesity Profile, Public Health England Public Health Profiles 2021, https://fingertips.phe.org.uk/profile/national-child-measurement-programme, Crown copyright 2021.

1. Scholes S, Bajekal M. Synthetic estimation of healthy lifestyles indicators: User guide [Internet]. Available from: https://www.researchgate.net/publication/265658543

2. Marshall A, Economic and Social Data Service. Small area estimation using ESDS government surveys – An introductory guide. 2010.

3. Rahman A. Estimating small area health-related characteristics of populations: A methodological review. Geospatial Health. 2017;12(1).

4. Moon G, Quarendon G, Barnard S, Twigg L, Blyth B. Fat nation: Deciphering the distinctive geographies of obesity in England. Social Science and Medicine. 2007 Jul;65(1):20–31.

5. [Dataset] National Centre for Social Research (NatCen), University College London, Department of Epidemiology and Public Health. (2020) [data collection]. Health Survey for England, 2018. UK Data Service. SN: 8649, http://doi.org/10.5255/UKDA-SN-8649-1.

For peer review only

6. [Dataset] University of Cambridge, MRC Epidemiology Unit, NatCen Social Research. (2021). National Diet and Nutrition Survey Years 1-11, 2008-2019 [data collection].19th Edition. UK Data Service. SN: 6533, http://doi.org/10.5255/UKDA-SN-6533-19.

7. [Dataset] Sport England. (2020). Active Lives Survey, 2017-2018 [data collection]. UK Data Service. SN: 8651, http://doi.org/10.5255/UKDA-SN-8651-1.

8. Rahman A, Harding A. Small area estimation and microsimulation modeling. Boca Raton; 2019.

9. Charlton J. Use of the Census Samples of Anonymised Records (SARs) and survey data in combination to obtain estimates at local authority level. Vol. 30, Environment and Planning A. 1998.

10. Macintyre S, Ellaway A, Cummins S. Place effects on health: how can we conceptualise, operationalise and measure them? Vol. 55, Social Science & Medicine. 2002.

11. Twigg L, Moon G, Jones K. Predicting small-area health-related behaviour: a comparison of smoking and drinking indicators [Internet]. 2000. Available from: www.elsevier.com/locate/socscimed

12. Edwards KL, Clarke GP, Thomas J, Forman D. Internal and External Validation of Spatial Microsimulation Models: Small Area Estimates of Adult Obesity. Applied Spatial Analysis and Policy. 2011 Nov;4(4):281–300.

13. Whitworth A, Aitken G, Mclennan D, Taylor J. Evaluations and improvements in small area estimation methodologies, National Centre for Research Methods Methodological Review paper. 2013.

14. [Dataset] Office for National Statistics. (2014). 2011 Census Microdata Individual Safeguarded Sample (Regional): England and Wales [data collection]. UK Data Service. SN: 7605, DOI:10.5255/UKDA-SN-7605-1.

15. Scarborough P, Allender S, Rayner M, Goldacre M. Validation of model-based estimates (synthetic estimates) of the prevalence of risk factors for coronary heart disease for wards in England. Health and Place. 2009;15(2):596–605.

16. English Indices of Deprivation 2019, Department for Communities and Local Government, Crown Copyright 2019, https://www.gov.uk/government/statistics/english-indices-of-deprivation-2019.

17. Partha D, Norton. Edward, Manning W. Health Econometrics Using Stata. College Station, Texas, 2017.

18. Mohammad N, Mcgivern L. Comparing Gamma and Log-Normal GLMs in R Using Simulation and Real Data Set.

19. Mansournia MA, Nazemipour M, Naimi AI, Collins GS, Campbell MJ. Reflection on modern methods: Demystifying robust standard errors for epidemiologists. International Journal of Epidemiology. 2021 Feb 1;50(1):346–51.

20. Edwards KL, Clarke GP. The design and validation of a spatial microsimulation model of obesogenic environments for children in Leeds, UK: SimObesity. Social Science and Medicine. 2009 Oct;69(7):1127–34.

For peer review only

21. Moon G, Twigg L, Jones K, Aitken G, Taylor J. The utility of geodemographic indicators in small area estimates of limiting long-term illness. Social Science and Medicine. 2019 Apr 1;227:47–55.

22. Whitworth A, Carter E, Ballas D, Moon G. Estimating uncertainty in spatial microsimulation approaches to small area estimation: A new approach to solving an old problem. Computers, Environment and Urban Systems. 2017 May 1;63:50–7.

23. Caleyachetty R, Barber TM, Mohammed NI, Cappuccio FP, Hardy R, Mathur R, et al. Ethnicity-specific BMI cutoffs for obesity based on type 2 diabetes risk in England: a population-based cohort study. The Lancet Diabetes and Endocrinology. 2021 Jul 1;9(7):419–26.

24. Obesity Profile, Public Health England Public Health Profiles 2021, https://fingertips.phe.org.uk/profile/national-child-measurement-programme, Crown copyright 2021.

Figure legend

Figure 1a/b: Distribution of BMI across the age range for three local authorities for females (1a, above) and males (1b, below): Manchester (IMD quintile 5 – highest deprivation), Oxford (IMD quintile 3) and South Gloucestershire (IMD quintile 1)

Figure 2: Scatter plot of mean measured BMI, uncalibrated estimate and calibrated estimate, with their associated 25th-75th centile range across the age range (for both sexes combined).

Figure 3a/b: Histogram of age-standardised BMI estimates for England for females (3a, above) and males (3b, below), including the equivalent histogram for measured BMI from NDNS 2014-16 for comparison.

Figure 4: Scatter plot of local areas' age-standardised percent of population with estimated BMI in the obese range against self-reported BMI in obese range in ALS 2017, with best fit line.

Figure 5: Comparison of results from Moon et al 2007(4) (left) with new estimates (right), showing the top decile of areas with the highest rates of obesity (above) and overweight (below). Left images reprinted from Moon G et al, Fat nation: Deciphering the distinctive geographies of obesity in England. Social Science and Medicine. 2007 Jul;65(1):20–31. Copyright (2007), with permission from Elsevier.

For peer review only

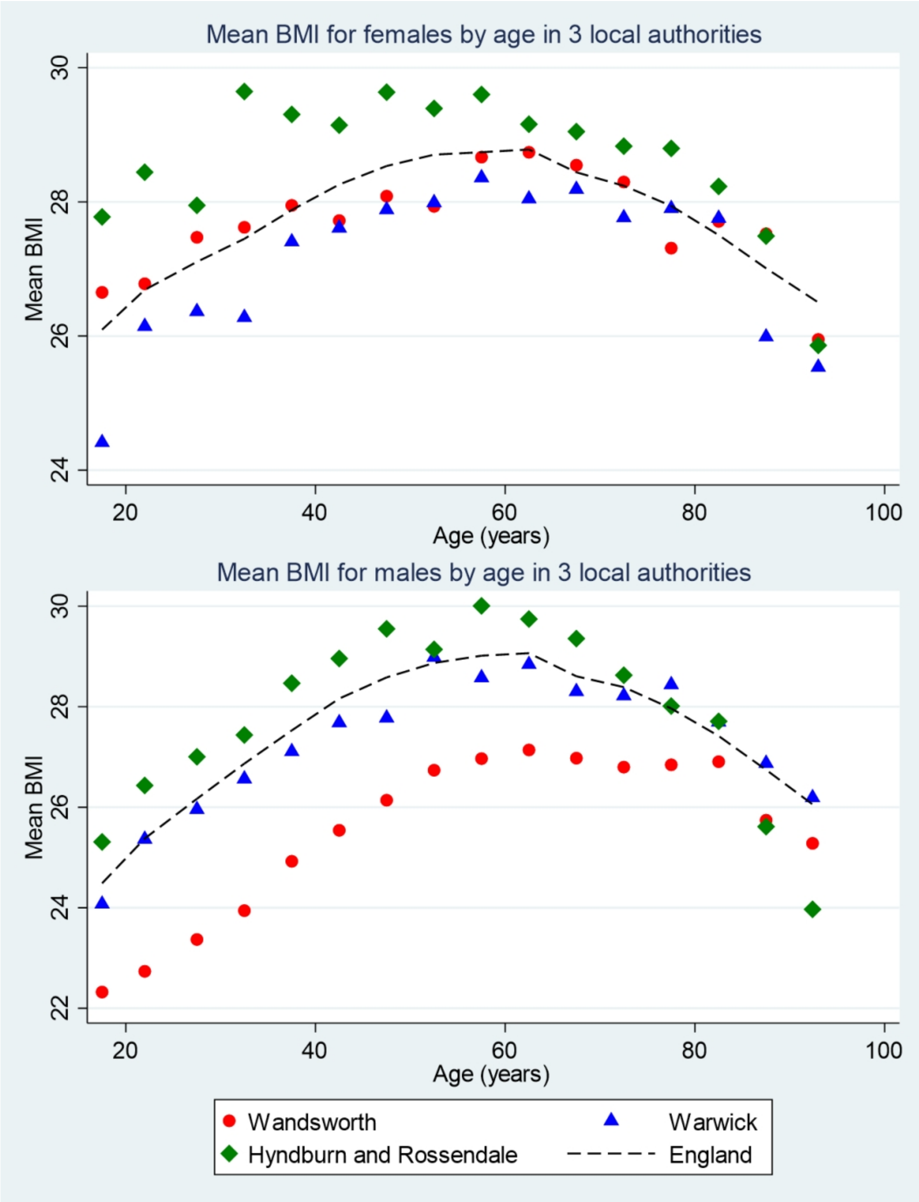

Figure 1a/b: Distribution of BMI across the age range for three local authorities for females (1a, above) and males (1b, below): Manchester (IMD quintile 5 – highest deprivation), Oxford (IMD quintile 3) and South Gloucestershire (IMD quintile 1)

387x506mm (85 x 85 DPI)

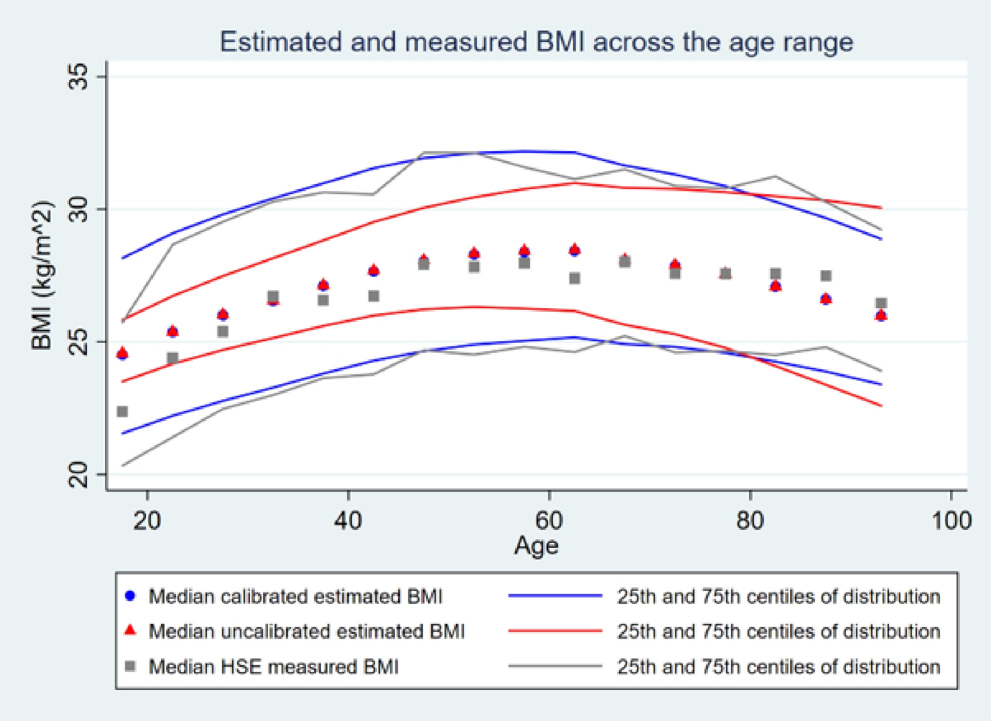

Figure 2: Scatter plot of mean measured BMI, uncalibrated estimate and calibrated estimate, with their associated 25th-75th centile range across the age range (for both sexes combined).

181x131mm (85 x 85 DPI)

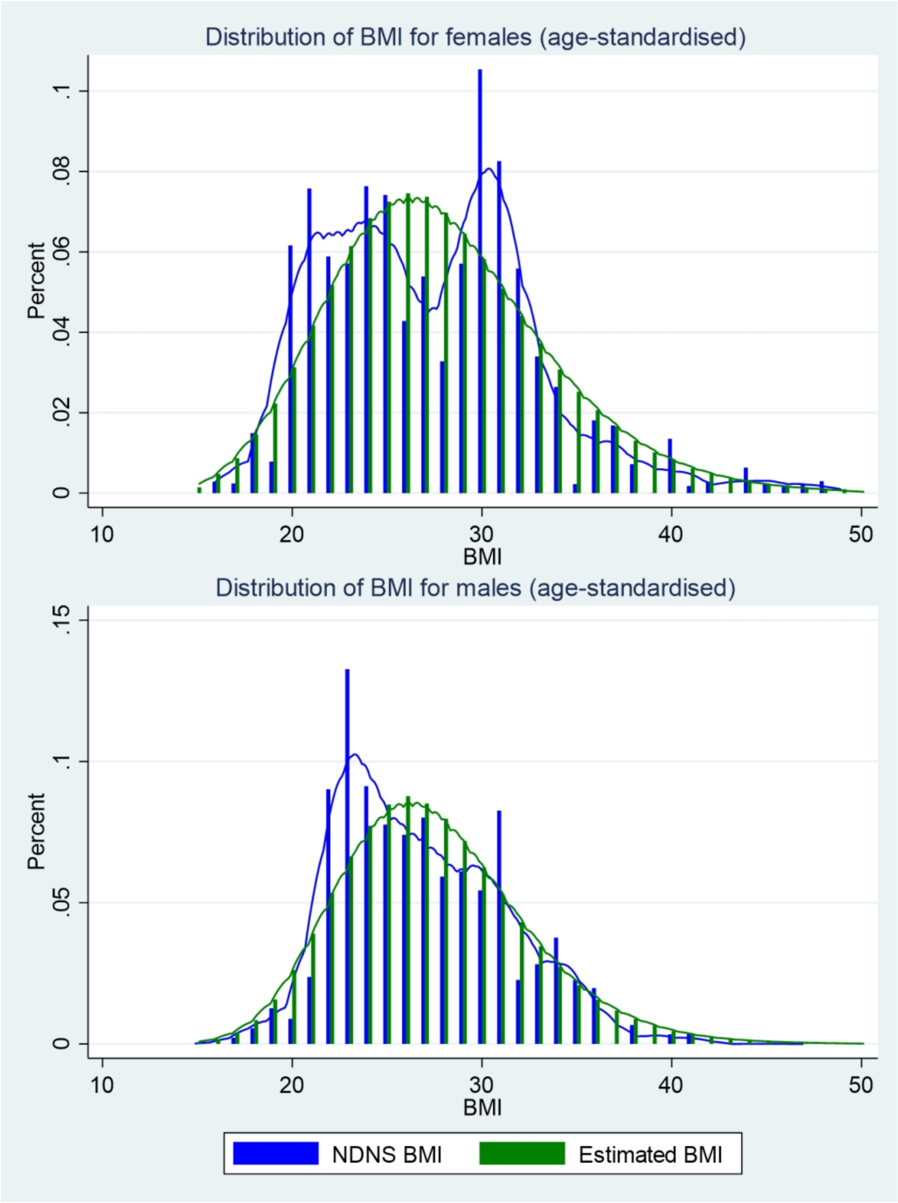

Figure 3a/b: Histogram of age-standardised BMI estimates for England for females (3a, above) and males (3b, below), including the equivalent histogram for measured BMI from NDNS 2014-16 for comparison.

388x519mm (85 x 85 DPI)

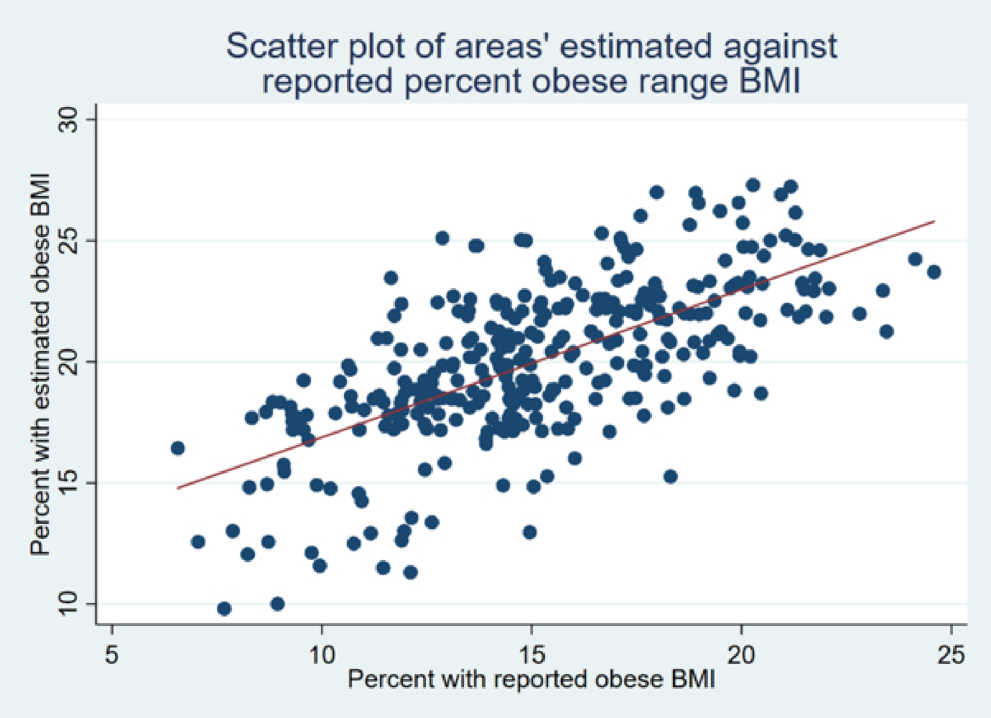

Figure 4: Scatter plot of local areas' age-standardised percent of population with estimated BMI in the obese range against self-reported BMI in obese range in ALS 2017, with best fit line.

181x130mm (85 x 85 DPI)

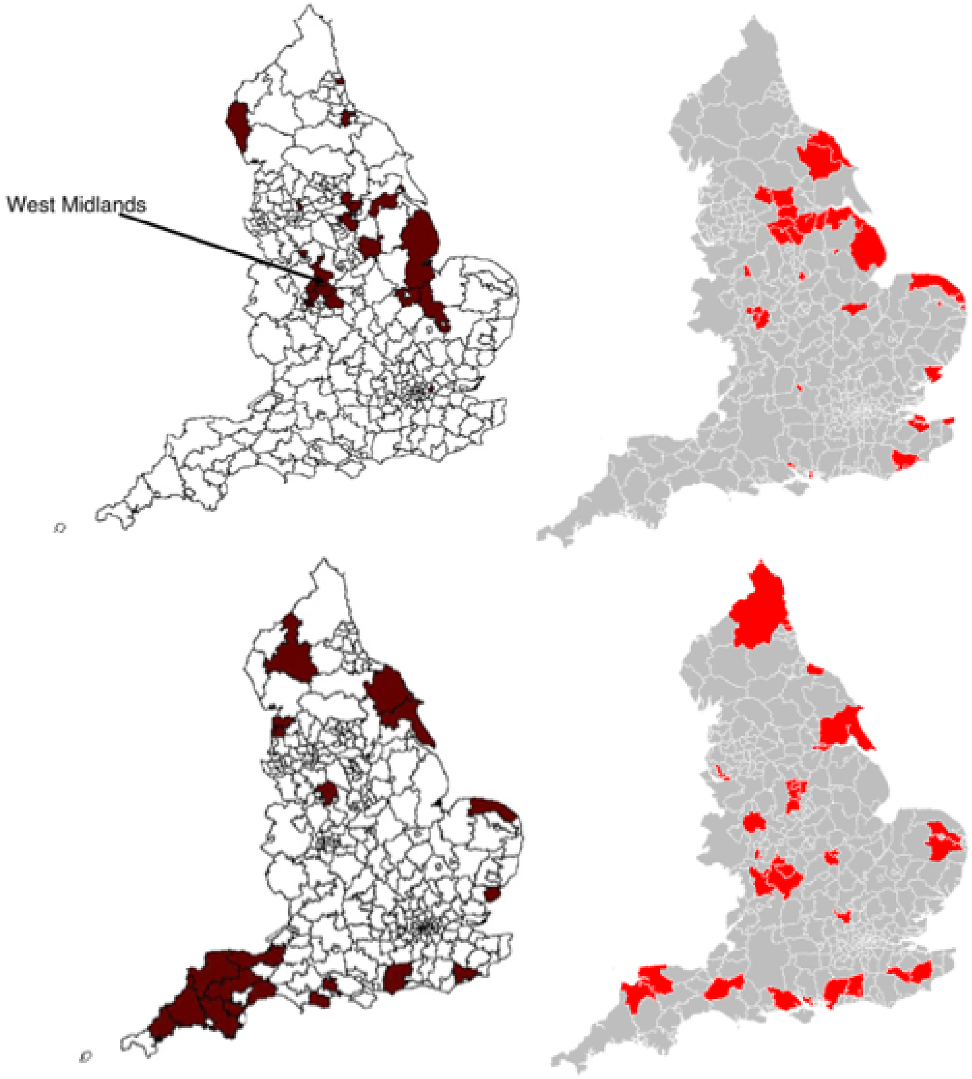

Figure 5: Comparison of results from Moon et al 2007(4) (left) with new estimates (right), showing the top decile of areas with the highest rates of obesity (above) and overweight (below). Left images reprinted from Moon G et al, Fat nation: Deciphering the distinctive geographies of obesity in England. Social Science and Medicine. 2007 Jul;65(1):20–31. Copyright (2007), with permission from Elsevier.

154x169mm (85 x 85 DPI)

Appendix tables

Appendix table 1: Variable mapping showing matched variables in HSE 2018 and the Census microdata and any adaptations to variables necessary to make them equivalent.

Characteristic	Census 2011 microdata<br>Variable name	Census value	Census label	New value	New Label
Age	AGEC	1	0-4 year olds	(Missing)	
		2	5-9 year olds	(Missing)	
		3	10-15 year olds	(Missing)	
		4	16-18 year olds	17.5	16-18 year olds
		5	19-24 year olds	22	19-24 year olds
		6	25-29 year olds	27.5	25-29 year olds
		7	30-34 year olds	32.5	30-34 year olds
		8	35-39 year olds	37.5	35-39 year olds
		9	40-44 year olds	42.5	40-44 year olds
		10	45- 49 year olds	47.5	45- 49 year olds
		11	50- 54 year olds	52.5	50- 54 year olds
		12	55-59 year olds	57.5	55-59 year olds
		13	60-64 year olds	62.5	60-64 year olds
		14	65-69 year olds	67.5	65-69 year olds
		15	70-74 year olds	72.5	70-74 year olds
		16	75-79 year olds	77.5	75-79 year olds
		17	80-84 year olds	82.5	80-84 year olds
		18	85-89 year olds	87.5	85-89 year olds
		19	90+	F=93.0<br>M=92.4(1)	90+ year olds
Sex	SEX	1	male	0	male
		2	female	1	female
Ethnicity	AGGETHPUK113	1	White	1	White
		2	Mixed/multiple ethnic groups	4	Mixed/multiple
		3	Asian/Asian British: Indian	3	Asian
		4	Asian/Asian British: Pakistani	3	Asian
		5	Asian/Asian British: Bangladeshi	3	Asian
		6	Asian/Asian British: Chinese	3	Asian
		7	Asian/Asian British: Other Asian	3	Asian
		8	Black/African/Caribbean/Black British: African	2	Black

		9	Black/African/Caribbean/Black British: Black Caribbean	2	Black
		10	Black/African/Caribbean/Black British: Other Black	2	Black
		11	Other ethnic group	5	Any other group
**Unemployed**	**ECOPUK1**	1	Econ. Active (excl. Students), Employee, Part-time	0	Not unemployed
		2	Econ. Active (excl. Students), Employee, Full-time	0	Not unemployed
		3	Econ. Active (excl. Students), Self-employed with employees, Part-time	0	Not unemployed
		4	Econ. Active (excl. Students), Self-employed with employees, Full-time	0	Not unemployed
		5	Econ. Active (excl. Students), Self-employed no employees, Part-time	0	Not unemployed
		6	Econ. Active (excl. Students), Self-employed no employees, Full-time	0	Not unemployed
		7	Econ. Active (excl. Students), Unemployed(2)	1	Unemployed
		8	Economically Active Full-time Students, In Employment	0	Not unemployed
		9	Economically Active Full-time Students, Unemployed(2)	1	Unemployed
		10	Economically Inactive, Retired	0	Not unemployed
		11	Economically Inactive, Student	0	Not unemployed
		12	Economically Inactive, Looking after home/family	0	Not unemployed
		13	Economically Inactive, Permanently sick/disabled	0	Not unemployed
		14	Economically Inactive, Other	0	Not unemployed
**Student**	**ECOPUK1**	1	Econ. Active (excl. Students), Employee, Part-time	0	Not a student
		2	Econ. Active (excl. Students), Employee, Full-time	0	Not a student
		3	Econ. Active (excl. Students), Self-employed with employees, Part-time	0	Not a student
		4	Econ. Active (excl. Students), Self-employed with employees, Full-time	0	Not a student

		5	Econ. Active (excl. Students), Self-employed no employees, Part-time	0	Not a student
		6	Econ. Active (excl. Students), Self-employed no employees, Full-time	0	Not a student
		7	Econ. Active (excl. Students), Unemployed(2)	0	Not a student
		8	Economically Active Full-time Students, In Employment	1	Student
		9	Economically Active Full-time Students, Unemployed(2)	1	Student
		10	Economically Inactive, Retired	0	Not a student
		11	Economically Inactive, Student	1	Student
		12	Economically Inactive, Looking after home/family	0	Not a student
		13	Economically Inactive, Permanently sick/disabled	0	Not a student
		14	Economically Inactive, Other	0	Not a student
**Has a degree or equivalent**	**hlqupuk11**	10	No academic or professional qualifications	0	
		11	Level 1 (0-4 GCSE, O level, or equivalents)	0	
		12	Level 2 (5+ GCSE, O level, 1 A level, or equivalents)	0	
		13	Apprenticeship	0	
		14	Level 3 (2+ A levels, or equivalents)	0	
		15	Level 4+ (degree, postgrad, professional quals)	1	Has a degree or equivalent
		16	Other (vocational/foreign/outside UK quals)	0	
		-9	Not applicable	(Missing)	
**Self-reported general health**	**HEALTH**	1	Very good	1	Very good
		2	Good	2	Good
		3	Fair	3	Fair
		4	Bad	4	Bad
		5	Very bad	5	Very bad
**Deprivation**	**IMDQUINTE**	1	Least deprived	1	Least deprived
		2	Second least	2	Second least
		3	Intermediate	3	Intermediate
		4	Second most	4	Second most
		5	Most deprived	5	Most deprived
		-8	unknown	(Missing)	

	**HSE 2017**				
	**Variable name**	**HSE value**	**HSE label**	**New value**	**New Label**
**Age**	**Age16g10**	1	0-1 year olds	(Missing)	
		2	2-4 year olds	(Missing)	
		3	5-7 year olds	(Missing)	
		4	8-10 year olds	(Missing)	
		5	11-12 year olds	(Missing)	
		6	13-15 year olds	(Missing)	
		7	16-19 year olds	18	16-19 year olds
		8	20-24 year olds	22.5	20-24 year olds
		9	25-29 year olds	27.5	25-29 year olds
		10	30-34 year olds	32.5	30-34 year olds
		11	35-39 year olds	37.5	35-39 year olds
		12	40-44 year olds	42.5	40-44 year olds
		13	45- 49 year olds	47.5	45- 49 year olds
		14	50- 54 year olds	52.5	50- 54 year olds
		15	55-59 year olds	57.5	55-59 year olds
		16	60-64 year olds	62.5	60-64 year olds
		17	65-69 year olds	67.5	65-69 year olds
		18	70-74 year olds	72.5	70-74 year olds
		19	75-79 year olds	77.5	75-79 year olds
		20	80-84 year olds	82.5	80-84 year olds
		21	85-89 year olds	87.5	85-89 year olds
		22	90+	F=93.0, M=92.4(1)	90+ year olds
**Sex**	**Sex**	1	Male	0	male
		2	Female	1	female
		3	Refused	(Missing)	
		4	Don't know	(Missing)	
		5	Not applicable	(Missing)	
**Ethnicity**	**Origin2**	1	White	1	White
		2	Black	2	Black
		3	Asian	3	Asian
		4	Mixed/multiple ethnic background	4	Mixed/multiple
		5	Any other ethnic group	5	Any other group
		6	Refusal	(Missing)	

		7	Don't know	(Missing)	
**Unemployed**	**Activb2**	1	Going to school or college full-time	0	Not unemployed
		2	In paid employment or self-employed	0	Not unemployed
		3	Doing unpaid work for family business	0	Not unemployed
		4	Waiting to take up paid work already obtained	0	Not unemployed
		5	Looking for paid work/ Govt. training scheme	1	Unemployed
		6	Intending to look for work, but temporary sickness	0	Not unemployed
		7	Permanently unable to work, long-term sickness	0	Not unemployed
		8	Retired from paid work	0	Not unemployed
		9	Looking after home or family	0	Not unemployed
		95	Doing something else (specify)	0	Not unemployed
		-9	No answer/refused	(Missing)	
		-8	Don't know	(Missing)	
		-1	Not applicable	(Missing)	
**Student**	**Activb2**	1	Going to school or college full-time	1	Student
		2	In paid employment or self-employed	0	Not a student
		3	Doing unpaid work for family business	0	Not a student
		4	Waiting to take up paid work already obtained	0	Not a student
		5	Looking for paid work/ Govt. training scheme	0	Not a student
		6	Intending to look for work, but temporary sickness	0	Not a student
		7	Permanently unable to work, long-term sickness	0	Not a student
		8	Retired from paid work	0	Not a student
		9	Looking after home or family	0	Not a student
		95	Doing something else (specify)	0	Not a student
		-9	No answer/refused	(Missing)	
		-8	Don't know	(Missing)	
		-1	Not applicable	(Missing)	
**Has a degree or equivalent**	**topqual3**	1	NVQ4/NVQ5/Degree or equiv	1	Has a degree or equivalent

		2	Higher ed below degree	0	
		3	NVQ3/GCE A Level equiv	0	
		4	NVQ2/GCE O Level equiv	0	
		5	NVQ1/CSE other grade equiv	0	
		6	Foreign/other	0	
		7	No qualification	0	
		-9	Refused	(Missing)	
		-8	Don't know	(Missing)	
		-1	Not applicable	(Missing)	
**Self-reported general health**	**GenHelf**	1	Very good	1	Very good
		2	Good	2	Good
		3	Fair	3	Fair
		4	Bad	4	Bad
		5	Very bad	5	Very bad
		-9	Refused	(Missing)	
		-8	Don't know	(Missing)	
		-1	Not applicable	(Missing)	
**Deprivation**	**(Merged from external dataset**(15)**)**	1	Least deprived - 0.48 ->8.37	1	Least deprived
		2	Second least - 8.37 -> 13.92	2	Second least
		3	Intermediate - 13.92- >21.43	3	Intermediate
		4	Second most - 21.43 -> 33.88	4	Second most
		5	Most deprived - 33.88 -> 92.60	5	Most deprived

(1) Average age for over-90s in most recent ONS mid-year estimates of the England population is 93.0 years for females and 92.4 years for males.

(2) International Labour Organisation definition: seeking work and ready to start in 2 weeks, and Waiting to start a job already obtained and available to start within 2 weeks.

Appendix table 2: Percent of missing data in source datasets by variable used (England only, respondents aged 17 and over). *IMD was merged into Census data at the lower tier local authority level from ONS data.(15)

Dataset: Census microdata		HSE 2018	
Variable	% missing	Variable	% missing
age	0.00	age	0.00
sex	0.00	sex	0.00
ethnicity	1.28	ethnicity	0.39
student	1.28	student	0.21

has degree	1.28	has degree	0.46
health	1.28	health	0.04
No of cars	2.21	No of cars	0.06
IMD*	-	IMD	0.00
		BMI	18.33

Appendix table 3: Percent of missing data for the BMI variable used in HSE 2018 (BMIval2) by demographic group

Breakdown variable	Total records	Of which percent missing
Age		
18	281	17.8
22.5	366	19.4
27.5	487	17.5
32.5	581	18.1
37.5	647	14.2
42.5	684	15.8
47.5	668	15.3
52.5	686	17.9
57.5	733	16.2
62.5	634	17.5
67.5	614	14.3
72.5	625	20.0
77.5	444	22.3
82.5	298	30.5
87.5	177	36.2
90+	72	45.9
Sex		
Female	4461	18.7
Male	3536	17.8
IMD quintile		
1 (lowest deprivation)	1653	15.5
2	1707	18.3
3	1554	19.0
4	1557	19.1
5	1526	20.1

Appendix table 4: Baseline population characteristics. Age given as mean (median, interquartile range). Remaining variables given as number (percent of total).

Number	BMI up to 25 N= 2,198	BMI over 25 N= 4,333	Total N= 6,531
Age (years)	42.5 (32.5,62.5)	52.5 (42.5,67.5)	52.5 (37.5,67.5)
Males	1,337 (60.8%)	2,289 (52.8%)	3,626 (55.5%)
Ethnicity			
White	1,916 (87.2%)	3,828 (88.5%)	5,744 (88.1%)
Black	47 (2.1%)	131 (3.0%)	178 (2.7%)
Asian	184 (8.4%)	288 (6.7%)	472 (7.2%)
Mixed	34 (1.5%)	47 (1.1%)	81 (1.2%)
Any other	15 (0.7%)	33 (0.8%)	48 (0.7%)
IMD quintile			
1 (Least deprived)	501 (22.8%)	850 (19.6%)	1,351 (20.7%)
2	512 (23.3%)	931 (21.5%)	1,443 (22.1%)
3	433 (19.7%)	825 (19.0%)	1,258 (19.3%)
4	393 (17.9%)	867 (20.0%)	1,260 (19.3%)
5 (Most deprived)	359 (16.3%)	860 (19.8%)	1,219 (18.7%)
Self-rated health			
Very good	971 (44.2%)	1,238 (28.6%)	2,209 (33.8%)
Good	856 (39.0%)	1,880 (43.4%)	2,736 (41.9%)
Fair	259 (11.8%)	876 (20.2%)	1,135 (17.4%)
Bad	82 (3.7%)	253 (5.8%)	335 (5.1%)
Very bad	29 (1.3%)	86 (2.0%)	115 (1.8%)

For peer review only

Appendix figures

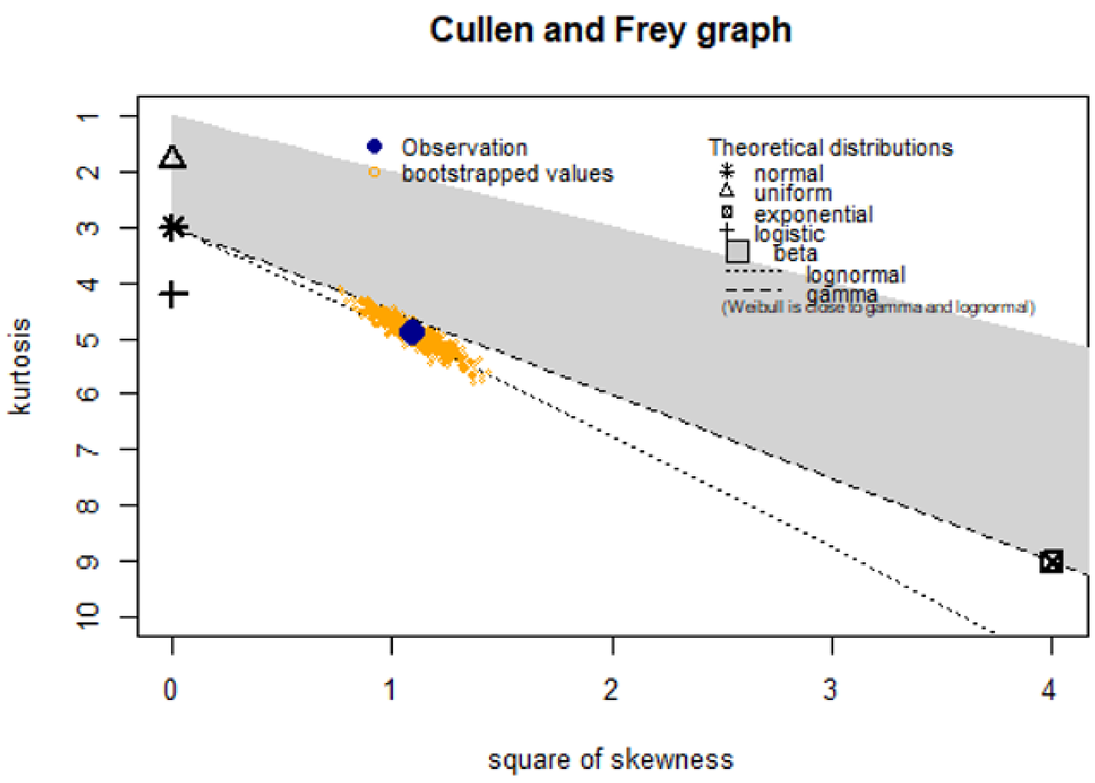

Appendix figure 1: Cullen-Frey plot exploring the mean-variance relationship of the model dependent BMI variable from HSE 2018, showing a marginally closer conformity with a lognormal than gamma distribution.

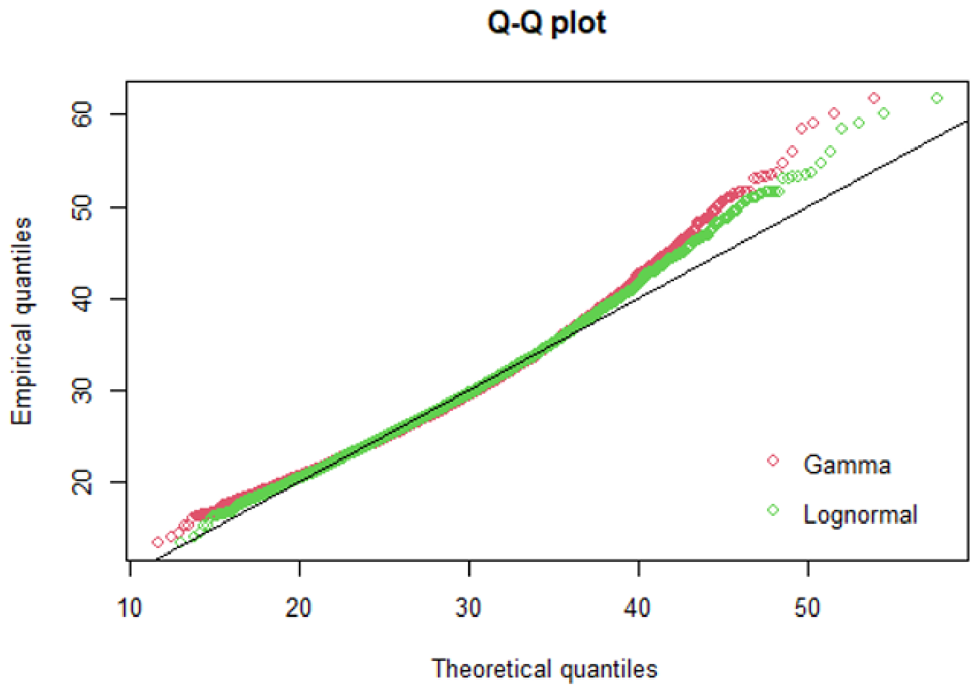

Appendix figure 2: Q-Q plot exploring the mean-variance relationship of the model dependent BMI variable from HSE 2018, detailing the deviation from gamma and lognormal distributions, showing a marginally closer conformity with a lognormal than gamma distribution.

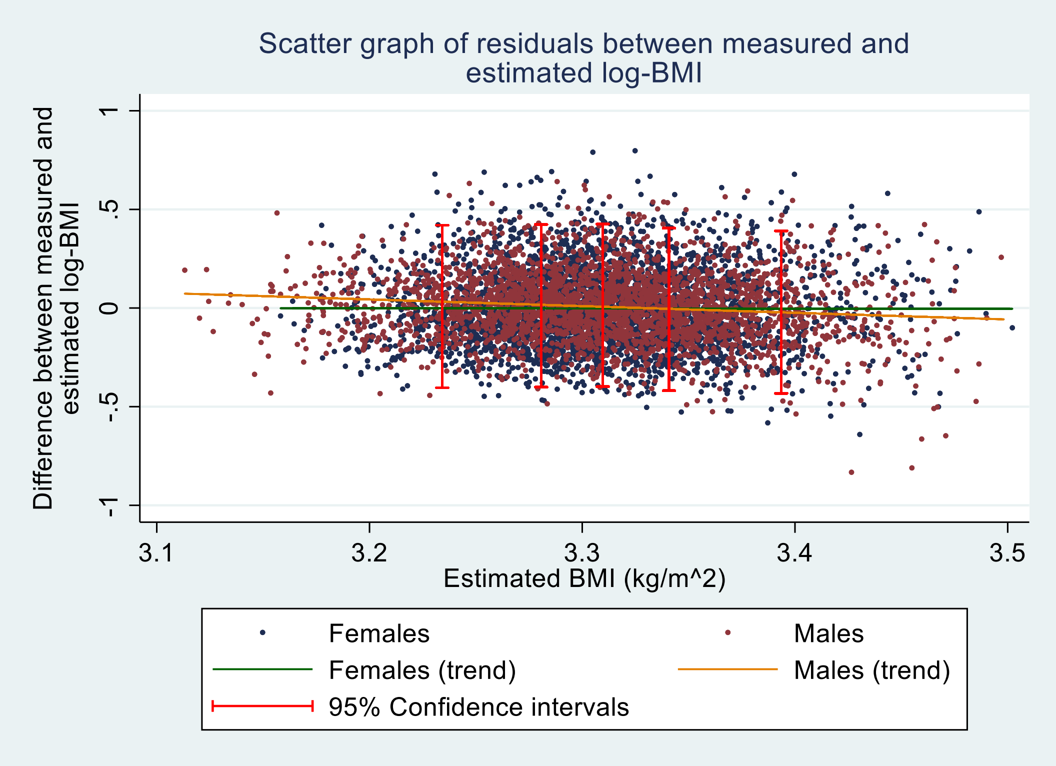

Appendix figure 3: Plot of residuals between measured log-BMI in the HSE 2018 and model-based estimates.

For peer review only

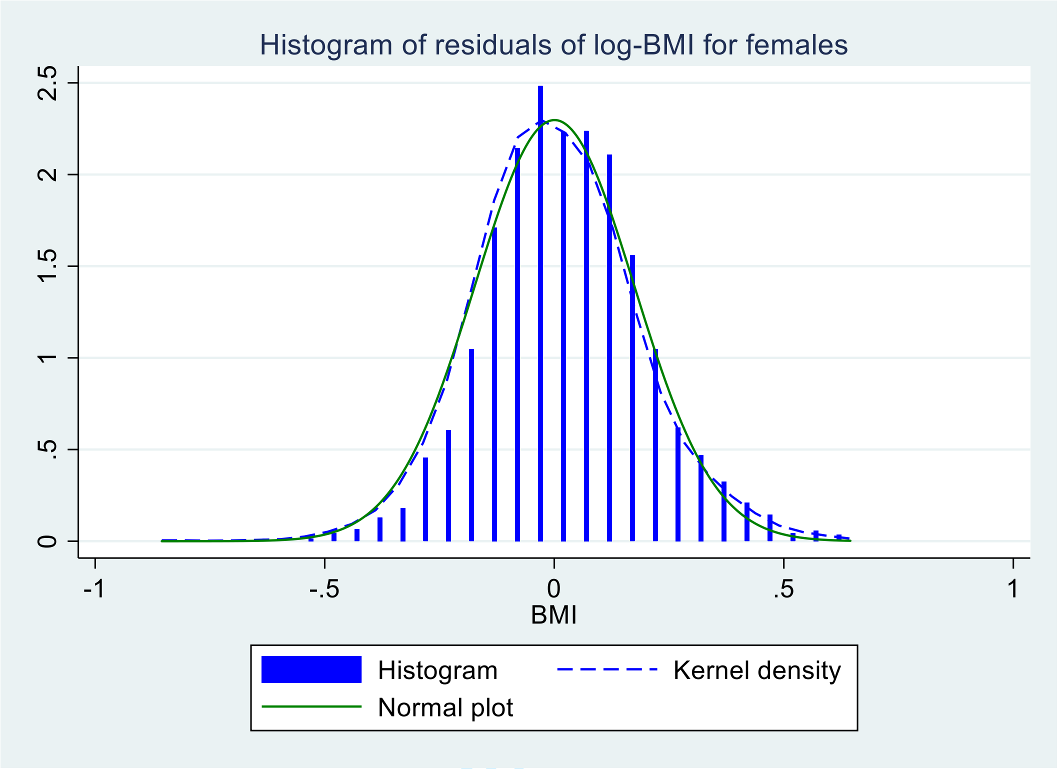

Appendix figure 4a: Histograms of residuals of between measured and estimated log-BMI for females to with kernel density and normal plot to examine for deviation from normality.

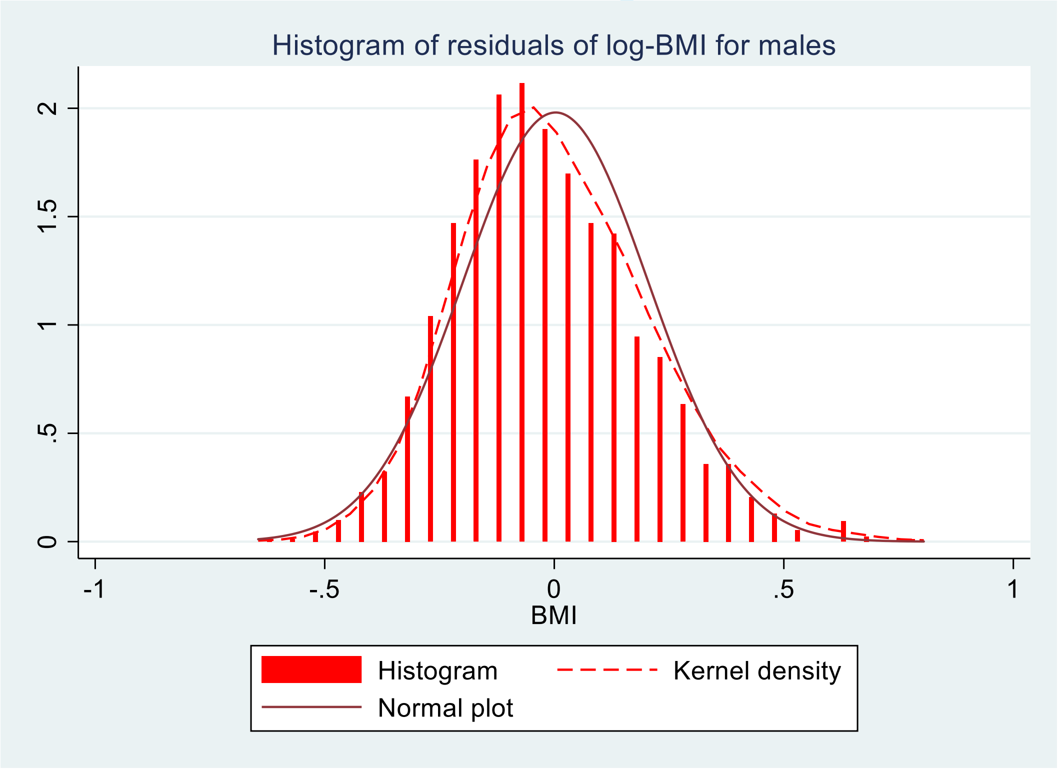

Appendix figure 4b: Histograms of residuals of between measured and estimated log-BMI for males to with kernel density and normal plot to examine for deviation from normality.

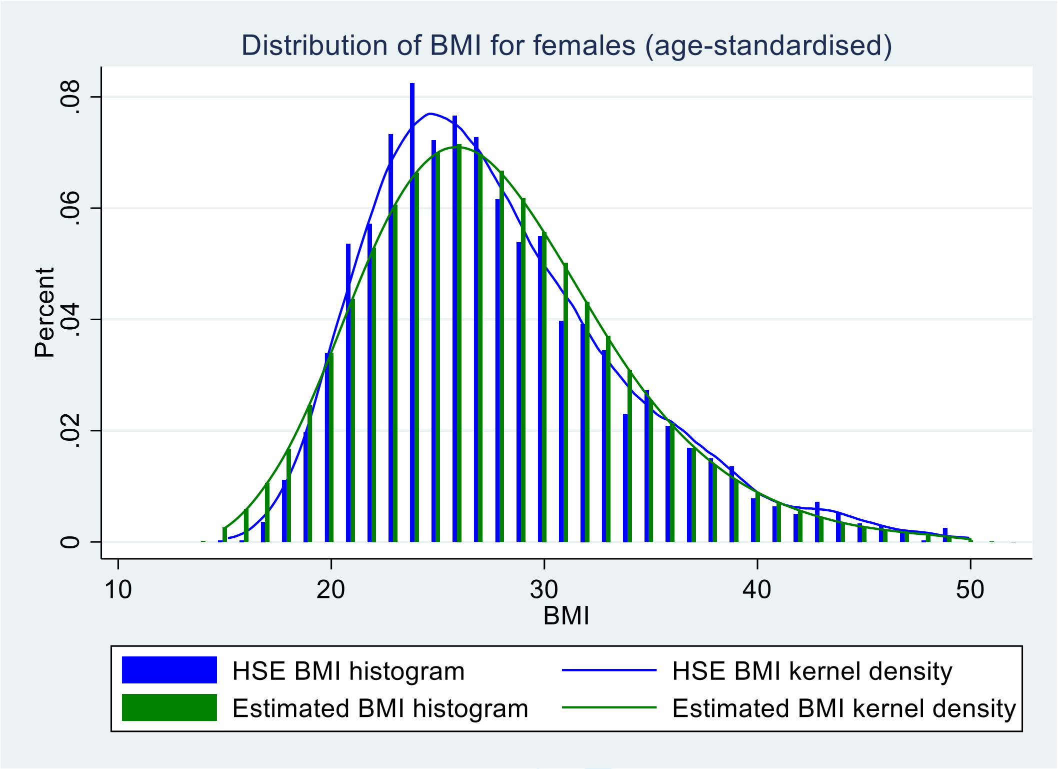

Appendix figure 5a: Histogram of age- and sex-standardised BMI estimates for England for females, including the equivalent histogram for the HSE 2018 BMI data for comparison.

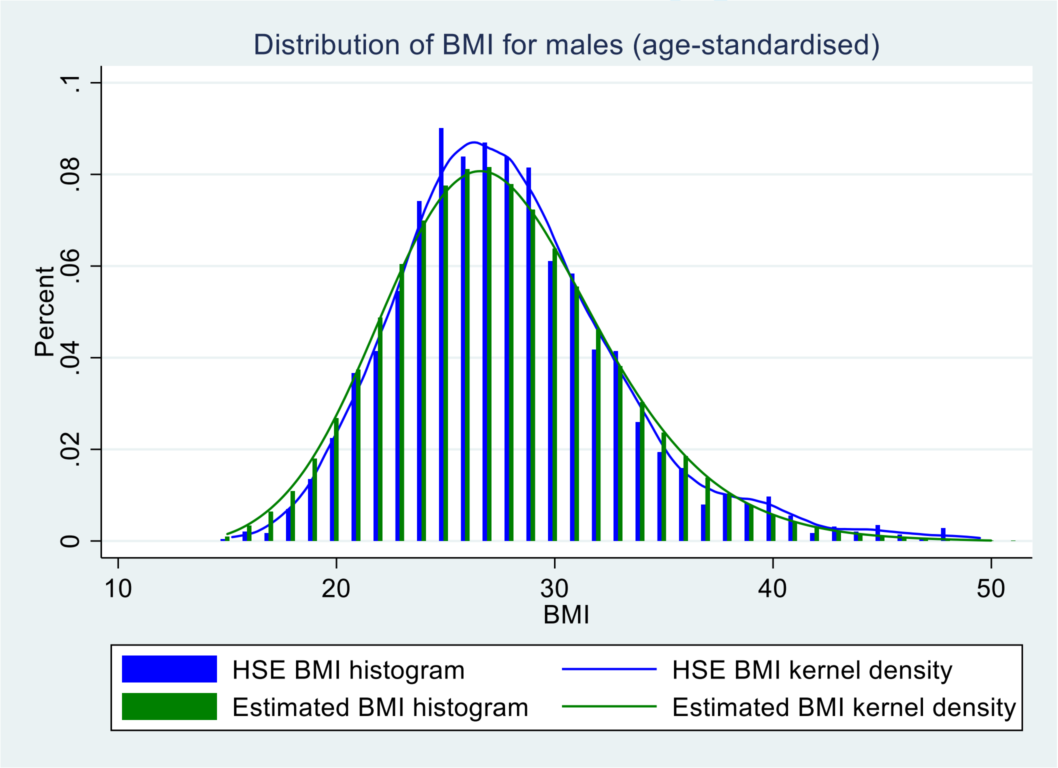

Appendix figure 5b: Histograms of age- and sex-standardised BMI estimates for England for males, including the equivalent histogram for the HSE 2018 BMI data for comparison.

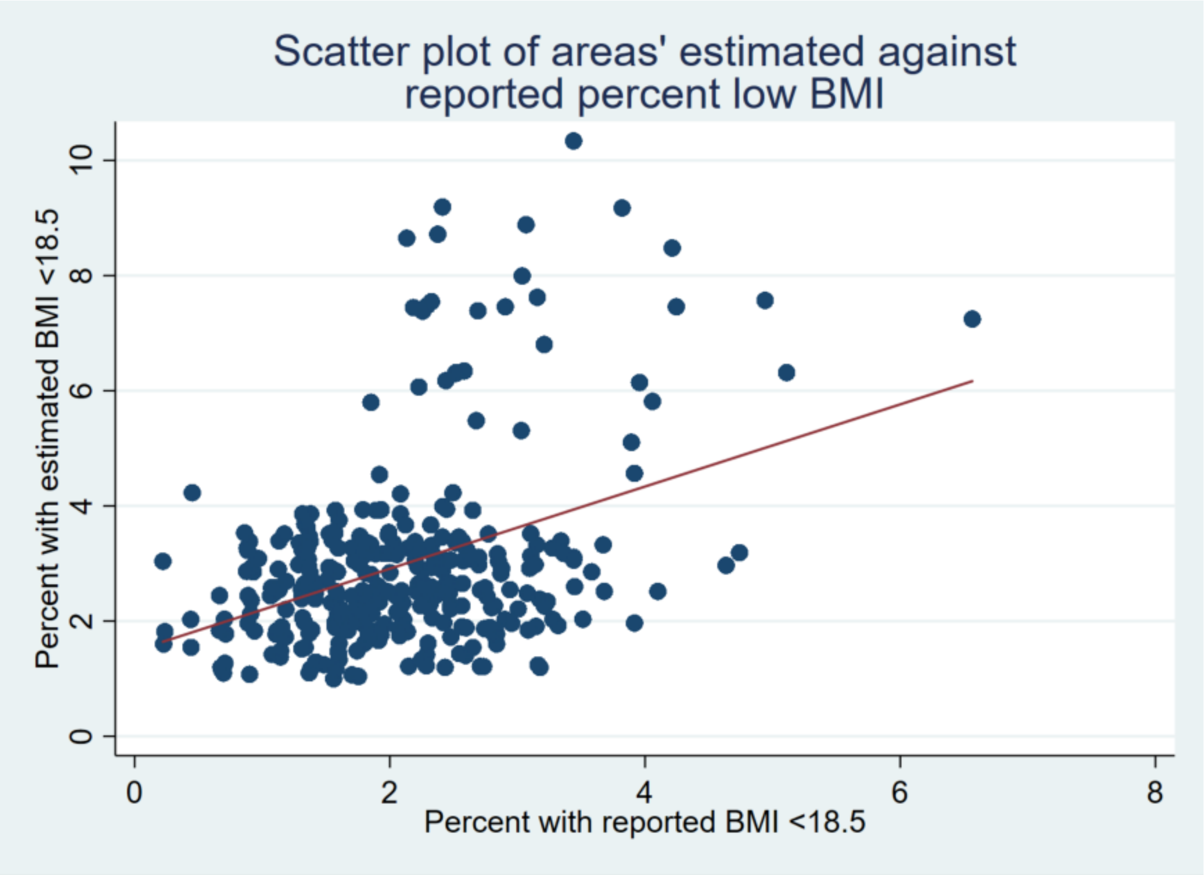

Appendix figure 6a: Scatter plot of the relationship between local areas' estimated versus reported percent of people with BMI <18.5.

For peer review only

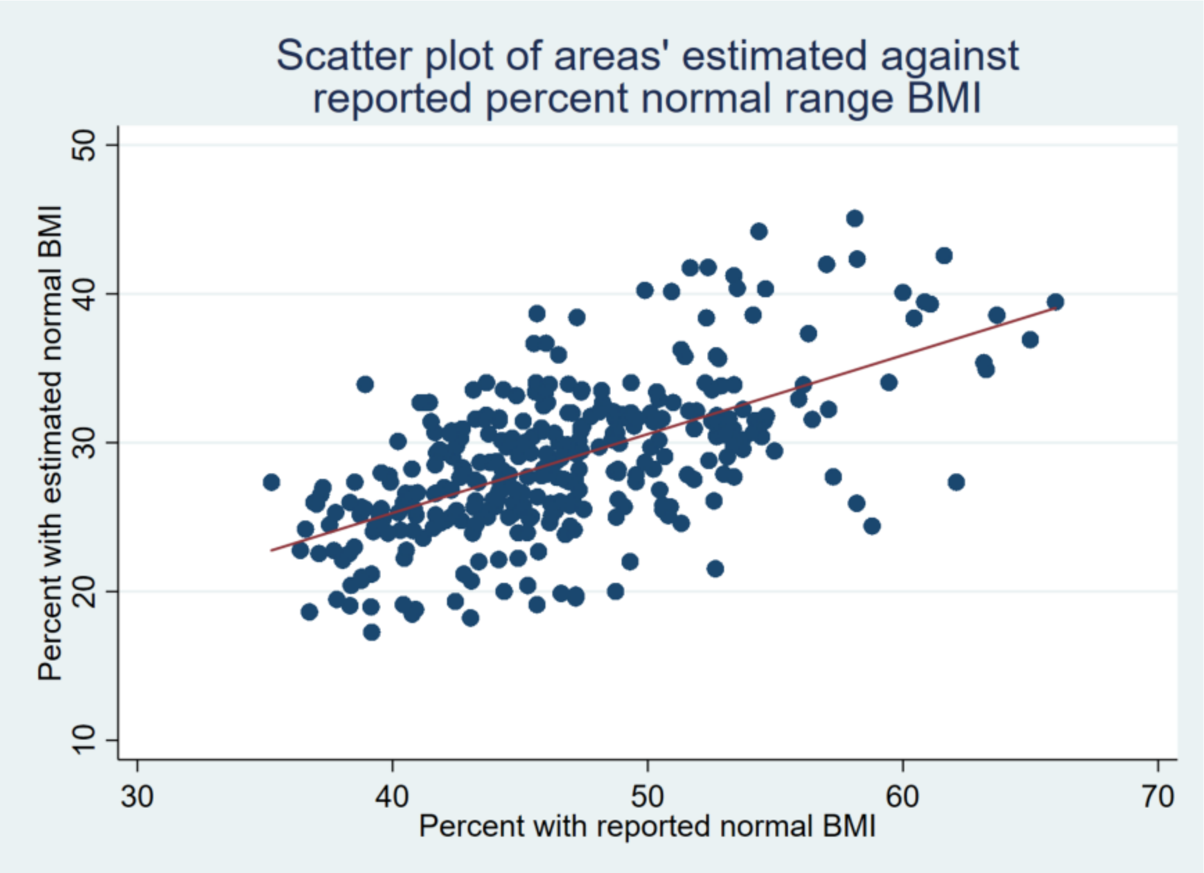

Appendix figure 6b: Scatter plot of the relationship between local areas' estimated versus reported percent of people with BMI in the normal range.

For peer review only

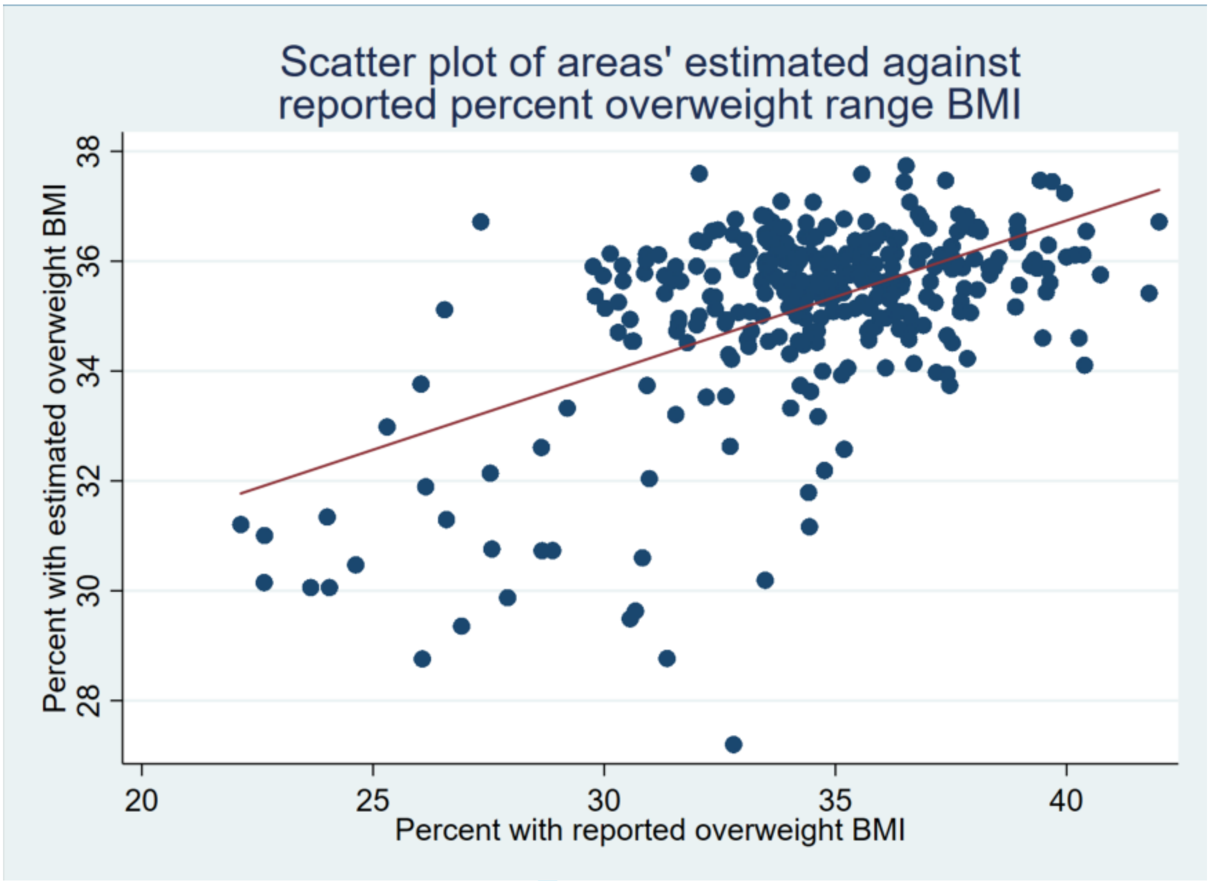

Appendix figure 6c: Scatter plot of the relationship between local areas' estimated versus reported percent of people with BMI in the overweight range.

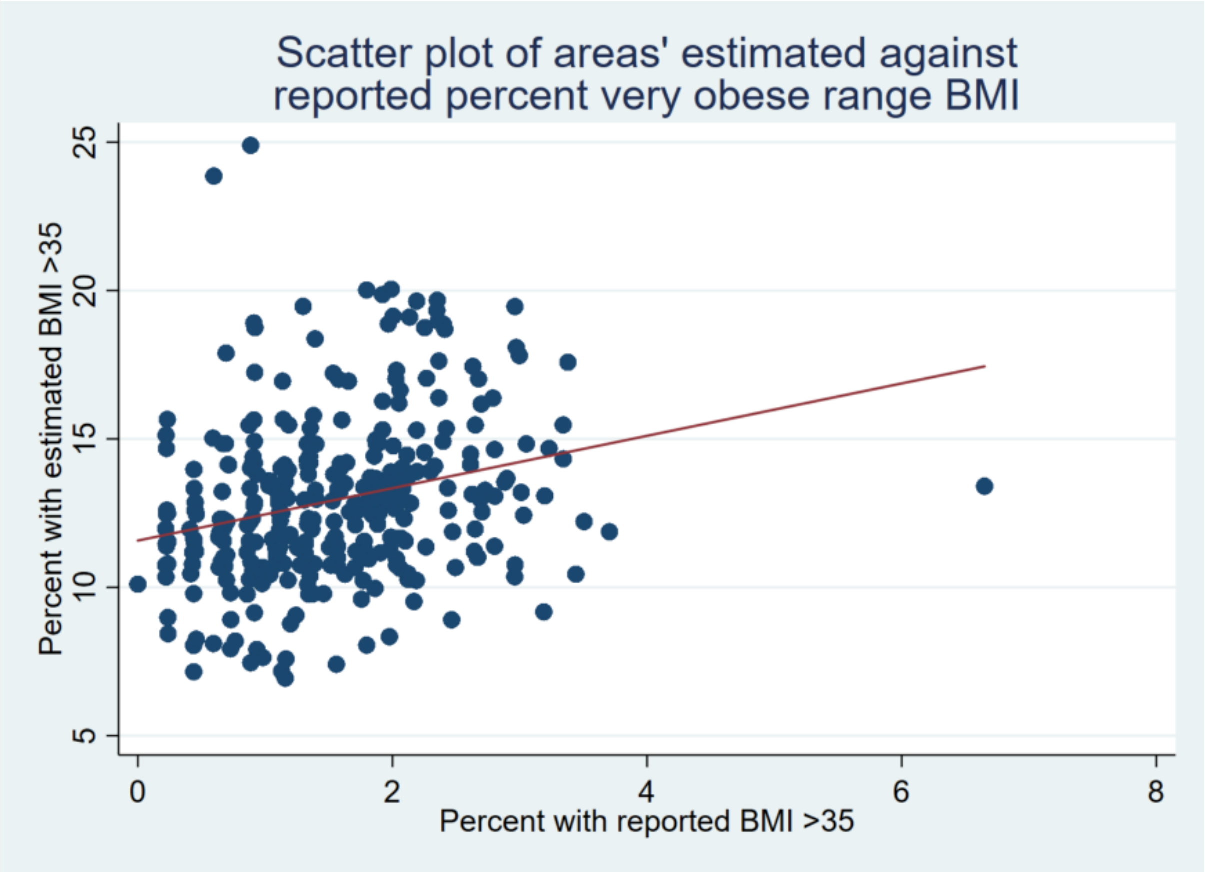

Appendix figure 6d: Scatter plot of the relationship between local areas' estimated versus reported percent of people with BMI >35.

For peer review only
